# Herb-target virtual screening and network pharmacology for prediction of molecular mechanism of Danggui Beimu Kushen Wan for prostate cancer

**DOI:** 10.1038/s41598-021-86141-1

**Published:** 2021-03-23

**Authors:** Hong Li, Andrew Hung, Angela Wei Hong Yang

**Affiliations:** 1 Discipline of Chinese Medicine, School of Health and Biomedical Sciences, RMIT University, PO Box 71, Bundoora, VIC 3083 Australia; 2 School of Science, RMIT University, Melbourne, VIC 3000 Australia

**Keywords:** Cancer therapy, Urological cancer, Drug screening, Prostate, Urogenital diseases, Drug development, Molecular medicine, Cancer

## Abstract

Prostate cancer (PCa) is a cancer that occurs in the prostate with high morbidity and mortality. Danggui Beimu Kushen Wan (DBKW) is a classic formula for patients with difficult urination including PCa. This study aimed to investigate the molecular mechanisms of DBKW for PCa. We obtained DBKW compounds from our previous reviews. We identified potential targets for PCa from literature search, currently approved drugs and Open Targets database and filtered them by protein–protein interaction network analysis. We selected 26 targets to predict three cancer-related pathways. A total of 621 compounds were screened via molecular docking using PyRx and AutoDock Vina against 21 targets for PCa, producing 13041 docking results. The binding patterns and positions showed that a relatively small number of tight-binding compounds from DBKW were predicted to interact strongly and selectively with three targets. The top five high-binding-affinity compounds were selected to generate a network, indicating that compounds from all three herbs had high binding affinity against the 21 targets and may have potential biological activities with the targets. DBKW contains multi-targeting agents that could act on more than one pathway of PCa simultaneously. Further studies could focus on validating the computational results via experimental studies.

## Introduction

Prostate cancer (PCa) is a cancer that occurs in the prostate, with more than one million new cases diagnosed in 2018 across the world^1^. In developed countries, the morbidity of PCa is high, which may be because prostate-specific antigen screening tests are undertaken as part of routine physical examinations^1^. PCa is a significant health problem globally, which brings a huge economic burden to society^2^. Males from certain racial/ethnic groups are more likely to be affected by PCa than others, as are people in lower socio-economic groups^3^. PCa patients present dysuria, frequent micturition, urgency, haematuria, urinary incontinence or acute urinary retention^4^. Recent studies pointed out that chronic prostatitis is highly associated with PCa, as it is one of the possible risk factors of PCa^5,6^ and may result in increasing cell proliferation and even potential carcinogenesis^7–9^. Furthermore, there is evidence that males suffering from chronic prostatitis had a 30% higher probability of developing PCa^10^ while there are no direct links between benign prostatic hyperplasia and PCa^11^. As the understanding of PCa continues to deepen, a set of systematic and individualised routine treatments have been formed and recommended in clinical practice guidelines, such as active surveillance and observation, radiotherapy, surgery, androgen deprivation therapy, chemotherapy and immunotherapy^12^. However, they are associated with many adverse events, such as fatigue, neuropathy, stomatitis, diarrhoea, nausea, vomiting and headache^12^.

Due to limited therapeutic effects and adverse events associated with routine treatments^13,14^, an increasing number of PCa patients are seeking complementary and alternative medicine including Chinese herbal medicine (CHM) for the management and/or support of androgen deprivation therapy^15–17^. CHM potentially provides a wealth of bioactive natural compounds and has been used for the management of urination-related disorders for a long time period^18,19^. A recent systematic review involving 1224 patients reported that CHMs might delay the development of PCa, extend survival time and improve patients’ physical performance, without any adverse events^20^.

Danggui Beimu Kushen Wan (DBKW; Chinese Angelica, Fritillaria and Flavescent Sophora Pill), also known as Guimu Kushen Wan or Kushen Wan, is a classical herbal formula that was initially recorded in the Jin Gui Yao Lue (Synopsis of Prescriptions of the Golden Chamber; ZHANG, Zhongjing; 205 AD), containing Angelicae Sinensis Radix (ASR; Dang gui), Fritillariae Thunbergii Bulbus (FTB; Zhe bei mu) and Sophorae Flavescentis Radix (SFR; Ku shen)^21^. Our previously published reviews have revealed that this formula has been used for managing urinary-related disorders for thousands of years^22,23^ and it has been extended to manage a wide range of malignant tumours in clinical practice, such as PCa^24,25^, cervical cancer^24^, bladder cancer^24^, liver cancer^26^ and vulvar basal cell carcinoma^27^. Nowadays, it has still been widely used and shown to be effective in clincial practice at treating a number of urinary system diseases involving PCa^24^. However, the mechanisms of action of DBKW for the management of PCa have not been investigated. This study is the first time using herb-target virtual screening to identify potential inhibitors and attempt to explain the molecular mechanisms of a Chinese herbal formula for PCa management.

## Results and discussion

### Compounds identified from DBKW’s ingredients for molecular docking

A total of 818 constituents were retrieved from the published literature which utilised different methods (such as LC–MS, HPLC and UPLC-CAD) to identify chemical compounds from individual herbs of DBKW, including 408 compounds from ASR, 133 compounds from FTB, and 277 compounds from SFR^28^. After removal of duplicates, 764 components were identified. Among them, the structures of 113 compounds are unknown. Since elements could not be accurately docked with the current docking strategy employed, they were not selected. Therefore, 621 compounds were selected for molecular docking (Supplementary Figs. S1 to S5 and Tables S1 online).

### Potential targets for PCa

#### Candidate targets from literature search

Fourteen pharmacological studies were included in our published thesis^28^. Within the 14 included studies, none of the studies targeted PCa (Group A). There are nine studies focused on the treatment effects of DBKW on specific drug targets, including four studies on cancers except for PCa (Group B)^29–31^ and five studies on chronic prostatitis (Group C)^32–36^. Subsequently, no drug targets were in Group A because no studies were identified in literature search. Nonetheless, seven targets were identified in Group B (B-cell lymphoma/leukemia-2-associated X (BAX)^29,30^, B-cell lymphoma/leukemia-2 (BCL2)^30^, caspase 3 (CASP3), hypoxia inducible factor-1α (HIF1A)^30,31^, phosphatase and tensin homolog (PTEN), prostaglandin-endoperoxide synthase 2 (PTGS2)^29^, and tumour protein 53 (TP53)^30^) and seven targets in Group C (intercellular cell adhesion molecule-1 (ICAM1)^36^, interleukin 1β (IL1B)^37^, interleukin 2 (IL2)^33^, interleukin 8^33^, malondialdehyde^34^, superoxide dismutase^34^, and tumour necrosis factor-α^35^).

#### Candidate targets from currently approved drugs for PCa

Eighteen currently approved drugs for PCa in four treatment groups (androgen deprivation therapy, chemotherapy, immunotherapy and bone health) were identified and 21 drug targets for them were retrieved from the DrugBank database (Group D), including acid phosphatase prostate (ACPP), aryl hydrocarbon receptor (AHR), androgen receptor (AR), BCL2, cytochrome P450 family 17 subfamily A member 1 (CYP17A1), cytochrome P450 family 21 subfamily A member 2 (CYP21A2), cytochrome P450 family 19 subfamily A member 1 (CYP19A1), farnesyl diphosphate synthase (FDPS), geranylgeranyl diphosphate synthase 1 (GGPS1), gonadotropin releasing hormone receptor (GNRHR), potassium voltage-gated channel subfamily H member 2 (KCNH2), luteinizing hormone/choriogonadotropin receptor (LHCGR), microtubule associated protein 2, microtubule associated protein 4 (MAP4), microtubule associated protein tau (MAPT), nuclear receptor subfamily 1 group I member 2 (NR1I2), nuclear receptor subfamily 1 group I member 3, programmed cell death 1 (PDCD1), tumour necrosis factor superfamily member 11 (TNFSF11), tubulin alpha 4a (TUBA4A) and tubulin beta 1 class VI (TUBB1) (Supplementary Table S2 online).

#### Candidate targets after cross-comparison

**Figure 1:**
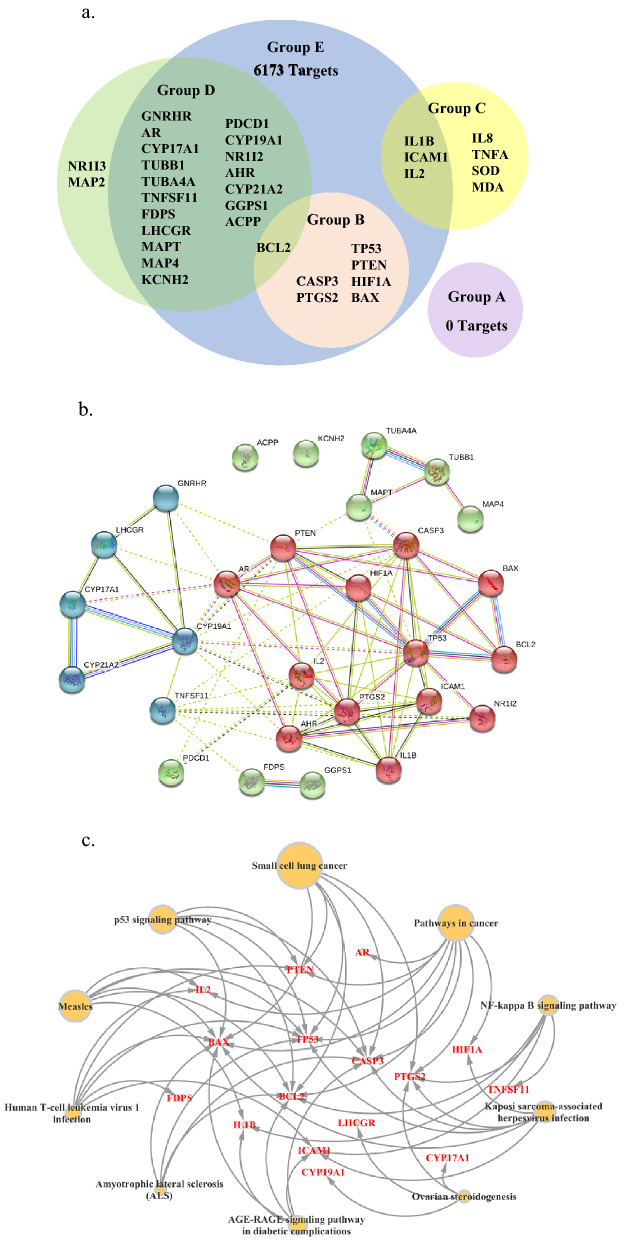
Potential target proteins and their network analyses. (**a**) Venn diagram of candidate drug targets for prostate cancer. Group A: Targets from studies of prostate cancer; Group B: Targets from studies of cancers except prostate cancer; Group C: Targets from studies of chronic prostatitis; Group D: Targets from currently approved drugs for prostate cancer; Group E: Targets under category of ‘prostate carcinoma’ in Open Targets database. (**b**) Protein–protein interaction network of drug targets for prostate cancer. This figure was generated by the STRING database. (**c**) Network of top 10 Kyoto Encyclopedia of Genes and Genomes pathways. *AR* androgen receptor, *ACPP* acid phosphatase prostate, *BAX* B-cell lymphoma-2 associated X, *BCL2* B-cell lymphoma-2, *CASP3* Caspase 3, *CYP17A1* Cytochrome P450 family 17 subfamily A member 1, *CYP21A2* Cytochrome P450 family 21 subfamily A member 2, *CYP19A1* Cytochrome P450 family 19 subfamily A member 1, *FDPS* farnesyl diphosphate synthase, *GGPS1* geranylgeranyl diphosphate synthase1, *GNRHR* gonadotropin releasing hormone receptor, *HIF1A* hypoxia inducible factor-1α, *ICAM1* intercellular cell adhesion molecule 1, *IL1B* interleukin 1β, *IL2* interleukin 2, *IL8* interleukin 8, *KCHN2* potassium voltage-gated channel subfamily H member 2, *LHCGR* luteinizing hormone/choriogonadotropin receptor, *MAP2* microtubule associated protein 2, *MAP4* microtubule associated protein 4, *MAPT* microtubule associated protein tau, *MDA* malondialdehyde, *NR1I2* nuclear receptor subfamily 1 group I member 2, *NR1I3* nuclear receptor subfamily 1 group I member 3, *PDCD1* programmed cell death 1, *PTEN* phosphatase and tensin homolog, *PTGS2* prostaglandin-endoperoxide synthase 2, *SOD* superoxide dismutase, *TNFA* tumour necrosis factor-α, *TNFSF11* tumour necrosis factor superfamily member 11, *TP53* tumour protein 53, *TUBA4A* tubulin alpha 4a, *TUBB1* tubulin beta 1 class VI.

A total of 7692 targets were listed in the category of ‘prostate carcinoma’ in the Open Targets database (Group E). All the targets identified from literature and the approved drugs (Groups A, B, C and D) were compared to the targets from the Open Targets database (Group E), respectively. After cross-comparison, 28 candidate drug targets were identified (Fig. 1a).

#### Protein–protein interaction (PPI) network of the targets

The PPI network of the 28 candidate targets is illustrated in Fig. 1b. Twenty-eight nodes and 79 edges are present, with a 5.64 average node degree and 0.604 average local clustering coefficient. Among the 28 drug targets, 26 of them have been clustered into one group, indicating that they may interact with each other. However, two of them (ACPP and KCNH2) did not have any interactions with others. Thus, only 26 of them were selected to perform subsequent computational analyses. A node in Fig. 1b stands for a target. The thickness of the edge between two proteins is proportional to the strength of evidence supports for the interaction of the two targets^38^.

#### Kyoto Encyclopedia of Genes and Genomes (KEGG) enrichment

There are 93 KEGG pathways which were identified, including 21 cancer-related pathways, 21 infectious disease-related pathways, 7 signal transduction-related pathways, 6 immune system-related pathways, 5 cell growth and death-related pathways, 5 endocrine system-related pathways and 28 other pathways. Cancer-related pathways and infectious disease-related pathways account for the highest proportion of all pathways (22.58% respectively) (Supplementary Table S3 online). Then, we selected the top 10 KEGG pathways based on their *p*-values to generate a network (Fig. 1c).

In Fig. 1c, the sizes of the nodes represent the *p*-values of the pathways. A larger node size indicates that it is more important in the network. When a target was identified in a specific KEGG pathway, an arrow was used from the nodes towards the target. Within the top 10 KEGG pathways, the first and second pathways were cancer-related pathways. Others included three infectious disease-related pathways, one cell growth and death-related pathway, one signal transduction-related pathway, one endocrine and metabolic disease-related pathway, one endocrine system-related pathway and one neurodegenerative disease-related pathway.

### Further selection of the 21 targets for molecular docking

After the PPI network analyses of the targets for PCa, 26 targets were identified. Characteristics of the 26 drug targets are listed in Table 1. Within the 26 targets, except for TNFSF11 which is a mouse and human chimera, 25 targets were from homo sapiens. There are ten targets with the highest associating score (1.00) in the Open Targets database, indicating that these targets had the strongest evidence from the 20 data sources for an association with PCa based on the evidence^39^. The structures of nine targets were obtained by homology modelling using the online SWISS-MODEL server, due to missing loop segments^40^. In addition, the only known structure of the tau peptide in PDB is of the fibrillar form, and not the likely native fold. Therefore, targets with this structure (MAPT and MAP4) were excluded from docking analyses. Further work is needed to predict or determine the native form of this protein for accurate docking studies. Also, three of the targets, including FDPS, GGPS1 and TNFSF11, are related to the bone health of PCa patients but not for original PCa nidus. Thus, these targets were excluded from docking studies as well. Future studies could focus on the investigation of the mechanisms of action of DBKW for these targets. Consequently, a total of 21 targets were selected for molecular docking, namely TP53 (T01), PTEN (T02), PTGS2 (T03), HIF1A (T04), BCL2 (T05), BAX (T06), CASP3 (T07), ICAM1 (T08), IL1B (T09), IL2 (T10), GNRHR (T11), AR (T12), CYP17A1 (T13), TUBB1 (T14), TUBA4A (T15), LHCGR (T18), PDCD1 (T19), CYP19A1 (T20), NR1I2 (T21), AHR (T22) and CYP21A2 (T23).

**Table 1:** Characteristics of 26 identified targets for prostate cancer.

No	Target	Open targets score	UniPortKB ID	PDB ID	PDB organism	SWISS-MODEL	Source
T01	TP53	1.00	P04637	4QO1	Human	Yes	Targets from literature
T02	PTEN	1.00	Q07820	1D5R	Human	Yes	Targets from literature
T03	PTGS2	1.00	P35354	5F19	Human	Yes	Targets from literature
T04	HIF1A	0.51	Q16665	4H6J	Human	No	Targets from literature
T05	BCL2	0.45	P10415	6FS0	Human	No	Targets from literature and drugs
T06	BAX	0.39	Q07812	4S0P	Human	Yes	Targets from literature
T07	CASP3	0.16	P42574	5IBC	Human	Yes	Targets from literature
T08	ICAM1	0.15	P05362	1MQ8	Human	Yes	Targets from literature
T09	IL1B	0.10	P01584	5MVZ	Human	No	Targets from literature
T10	IL2	0.09	Q0GK43	1PY2	Human	Yes	Targets from literature
T11	GNRHR	1.00	P30968	5VBL	Human	No	Targets from drugs
T12	AR	1.00	P10275	5CJ6	Human	No	Targets from drugs
T13	CYP17A1	1.00	P05093	3RUK	Human	No	Targets from drugs
T14	TUBB1	1.00	Q9H4B7	5IJ0	Human	Yes	Targets from drugs
T15	TUBA4A	1.00	P68366	5KMG	Human	Yes	Targets from drugs
T16	TNFSF11	1.00	O14788	5BNQ	Human (Chain A)	No	Targets from drugs
T17	FDPS	1.00	P14324	1YQ7	Human	No	Targets from drugs
T18	LHCGR	0.37	P22888	4AY9	Human	No	Targets from drugs
T19	PDCD1	0.33	Q15116	5WT9	Human	No	Targets from drugs
T20	CYP19A1	0.32	P11511	3EQM	Human	No	Targets from drugs
T21	NR1I2	0.06	O75469	6BNS	Human	No	Targets from drugs
T22	AHR	0.05	P35869	5NJ8	Human	No	Targets from drugs
T23	CYP21A2	0.03	P08686	5VBU	Human	No	Targets from drugs
T24	GGPS1	0.01	O95749	2Q80	Human	No	Targets from drugs
T25	MAPT	0.05	P10636	6HRE	Human	No	Targets from drugs
T26	MAP4	0.01	P27816	6HRE	Human	No	Targets from drugs

*AHR* aryl hydrocarbon receptor, *AR* androgen receptor, *BAX* B-cell lymphoma-2 associated X, *BCL2* B-cell lymphoma-2, *CASP3* Caspase 3, *CYP17A1* cytochrome P450 family 17 subfamily A member 1, *CYP21A2* cytochrome P450 family 21 subfamily A member 2, *CYP19A1* cytochrome P450 family 19 subfamily A member 1, *FDPS* farnesyl diphosphate synthase, *GGPS1* geranylgeranyl diphosphate synthase1, *GNRHR* gonadotropin releasing hormone receptor, *HIF1A* hypoxia inducible factor-1α, *ICAM1* intercellular cell adhesion molecule 1, *IL1B* interleukin 1β, *IL2* interleukin 2, *LHCGR* luteinizing hormone/choriogonadotropin receptor, *MAP4* microtubule associated protein 4, *MAPT* microtubule associated protein tau, *NR1I2* nuclear receptor subfamily 1 group I member 2, *PDCD1* programmed cell death 1, *PTEN* phosphatase and tensin homolog, *PTGS2* prostaglandin-endoperoxide synthase 2, *TNFSF11* tumour necrosis factor superfamily member 11, *TP53* tumour protein 53, *TUBA4A* tubulin alpha 4a, *TUBB1* tubulin beta 1 class VI.

### Molecular docking predictions

#### Characteristics of the docking results

All of the 621 compounds from DBKW with known or obtained structures were docked with the 21 targets for PCa, in order to predict their binding affinity, binding sites, orientation and molecular ligand conformation. A total of 13041 docking results were produced. In molecular docking, the lower (more negative) the binding energy in a docking result, the higher the binding affinity of the ligand that are predicted to exhibit against the target. The lowest binding scores between all compounds and all targets ranged from − 2.4 to − 13.3 kcal/mol, with an average binding score of − 6.58 kcal/mol. The top 11 high-binding-affinity compounds with multi-targeting activities included KA165, KB033, KB031, KB032, KB030, ZA08, ZA09, ZC12, KA013, KB034 and ZA16. In addition, from the point of view of each target, the total docking scores of all compounds to a target ranged from − 3619.5 to − 4877.5 kcal/mol, with an average score of − 4309.06 kcal/mol. The top 10 targets ranked by their total binding score were T03, T23, T21, T20, T02, T13, T05, T18, T14 and T11. T03 had a − 4877.5 kcal/mol total binding score, which was the lowest total binding score among the 21 targets (Table 2 and Supplementary Table S4 online).

**Table 2:** Details of docking results between 621 natural compounds from Danggui Beimu Kushen Wan and 21 targets for prostate cancer (kcal/mol).

Target ID	Target name	Total binding score	Min	25% percentile	Med	75% percentile	Max	Ave	SD	SEM	95% confidence interval of mean		Top 1 compound
											Lower	Upper	
T01	TP53	− 3773.0	− 9.1	− 6.8	− 5.8	− 4.7	− 2.9	− 5.76	1.29	0.05	− 5.86	− 5.66	KB032
T02	PTEN	− 4704.0	− 11.0	− 8.4	− 7.3	− 5.8	− 3.3	− 7.18	1.59	0.06	− 7.30	− 7.06	KB031
T03	PTGS2	− 4877.5	− 12.0	− 8.7	− 7.5	− 6.0	− 2.9	− 7.45	1.81	0.07	− 7.59	− 7.31	KA090
T04	HIF1A	− 3628.0	− 8.3	− 6.2	− 5.5	− 4.9	− 2.6	− 5.54	1.01	0.04	− 5.62	− 5.46	KB030
T05	BCL2	− 4604.1	− 11.5	− 8.2	− 7.3	− 6.0	− 2.4	− 7.03	1.47	0.06	− 7.14	− 6.92	KB031
T06	BAX	− 4020.8	− 9.3	− 7.0	− 6.3	− 5.2	− 2.9	− 6.14	1.17	0.05	− 6.23	− 6.05	KB034
T07	CASP3	− 4331.2	− 11.4	− 7.7	− 6.8	− 5.4	− 3.2	− 6.61	1.46	0.06	− 6.72	− 6.50	KB032
T08	ICAM1	− 3619.5	− 8.9	− 6.5	− 5.6	− 4.5	− 2.6	− 5.53	1.29	0.05	− 5.62	− 5.43	KA065
T09	IL1B	− 3839.6	− 8.9	− 6.9	− 6.0	− 4.6	− 2.4	− 5.86	1.36	0.05	− 5.97	− 5.76	KA165
T10	IL2	− 3861.2	− 8.7	− 6.7	− 5.9	− 5.1	− 2.6	− 5.89	1.07	0.04	− 5.98	− 5.81	KB033
T11	GNRHR	− 4426.3	− 11.0	− 7.7	− 6.8	− 5.7	− 2.9	− 6.76	1.37	0.05	− 6.86	− 6.65	ZA08
T12	AR	− 4280.4	− 9.5	− 7.5	− 6.7	− 5.7	− 2.6	− 6.53	1.21	0.05	− 6.63	− 6.44	KA013
T13	CYP17A1	− 4643.2	− 12.5	− 8.4	− 7.2	− 5.6	− 2.8	− 7.09	1.72	0.07	− 7.22	− 6.96	KB031
T14	TUBB1	− 4435.2	− 10.7	− 8.0	− 6.9	− 5.5	− 2.7	− 6.77	1.54	0.06	− 6.89	− 6.65	KB032
T15	TUBA4A	− 4330.8	− 9.7	− 7.8	− 6.8	− 5.4	− 2.9	− 6.61	1.4	0.05	− 6.72	− 6.50	KE012
T18	LHCGR	− 4530.2	− 11.4	− 8.2	− 7.1	− 5.5	− 3.0	− 6.92	1.63	0.06	− 7.04	− 6.79	KA165
T19	PDCD1	− 3793.4	− 8.5	− 6.6	− 5.9	− 5.0	− 2.7	− 5.79	1.08	0.04	− 5.87	− 5.71	KA165
T20	CYP19A1	− 4745.1	− 13.3	− 8.2	− 7.4	− 6.2	− 2.8	− 7.24	1.44	0.06	− 7.35	− 7.13	ZB27
T21	NR1I2	− 4844.4	− 12.1	− 8.5	− 7.6	− 6.2	− 2.6	− 7.40	1.6	0.06	− 7.52	− 7.27	KB034
T22	AHR	− 4329.0	− 10.4	− 7.7	− 6.7	− 5.5	− 2.8	− 6.61	1.39	0.05	− 6.72	− 6.50	KB031
T23	CYP21A2	− 4873.3	− 11.7	− 8.6	− 7.7	− 6.1	− 2.8	− 7.44	1.64	0.06	− 7.57	− 7.31	KB030

Corresponding target full names refer to Table 1. Corresponding compound names refer to Supplementary Tables S1 to S3 online.

*Ave* average value, *Max* maximum, *Med* median value, *Min* minimum, *SD* standard deviation, *SEM* standard error of mean.

#### Binding patterns of all compounds against 21 targets

**Figure 2:**
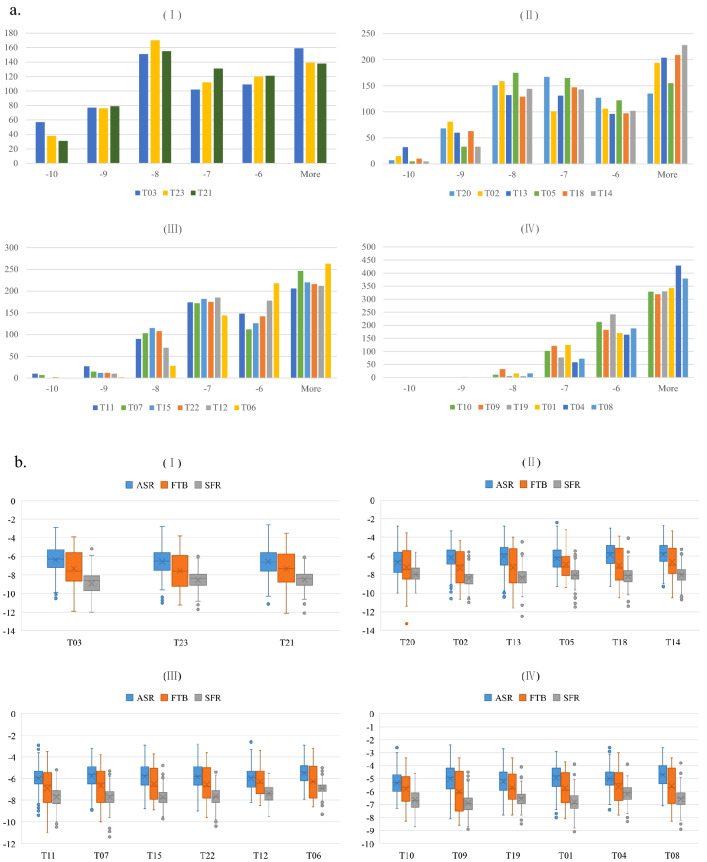
Binding patterns of all compounds against 21 targets. (**a**) Histograms of all compounds against 21 targets; (**b**) boxplots of all compounds from individual herbs of Danggui Beimu Kushen Wan against 21 targets. For Fig. 4a, x-axes stand for the predicted binding affinity values and y-axes stand for total number of ligands. For Fig. 4b, x-axes stand for the target proteins and y-axes represent the binding affinity for the compounds from each herb. *ASR* Angelicae Sinensis Radix, *FTB* Fritillariae Thunbergii Bulbus, *SFR* Sophorae Flavescentis Radix. (I): Top 1–3 targets; (II): Top 4–9 targets; (III): Top 10–15 targets; (IV): Top 16–21 targets; sequence was ranked by total binding scores. Corresponding target names refer to Table 1.

Binding patterns were analysed using histograms and boxplots to obtain an initial broad overview of the manner in which several key targets may interact with the DBKW compounds. The histograms were ranked by the total binding scores of the targets, to show the binding affinity distributions of all compounds for the 21 targets (Fig. 2a). Twenty-one targets were separated into four different groups (I–IV), with each group showing qualitatively different statistical distributions of the number of ligands with respect to binding affinity. Specifically, a relatively small number of tight-binding components from DBKW were predicted to interact strongly and selectively (binding score < − 9 kcal/mol) to the top nine targets (Group I and Group II). Targets in Group I involved more compounds (134, 114 and 110 compounds, respectively) than targets in Group II in this binding energy interval. In addition, most compounds from DBKW bound with intermediate affinity to targets in Groups I to III (− 7 to − 9 kcal/mol). However, for targets in Group IV, including T10, T09, T19, T01, T04, and T08, these targets interacted with a relatively large number of weak binding compounds (> − 6 kcal/mol). It is reasonable to hypothesise that the manner of interactions between compounds from DBKW and specific targets may be different. For targets in Groups I and II, which have the most high-binding-affinity compounds, a few herbal compounds may interact strongly and irreversibly with these three targets at some specific, highly attractive binding positions. Future studies could focus on investigating the mechanisms of action of DBKW for these top nine targets. In contrast, for other targets, especially targets in Group IV, dynamic interaction mechanisms, such as frequently reversible binding, dissociation and ‘ligand swapping’ at different binding positions, may occur between most of the compounds and these targets.

Additionally, boxplots were constructed to display in more detail the distribution of binding affinity values of compounds from the individual herbs of DBKW against the 21 targets, to obtain an idea of the manner in which each herb may differently interact with its proposed targets (Fig. 2b). In the boxplots, the interquartile range is used to identify the dispersion degree of the middle 50% of the data as well as non-normal distribution values. The smaller the interquartile range value, the more concentrated the data is in the middle 50%, while the larger the value, the more dispersed the data is. SFR has the smallest interquartile range for each target, followed by ASR and FTB, and compared to the location of the interquartile ranges of ASR and FTB the interquartile range of SFR is in the lower binding scores interval. This indicates that most compounds from SFR have higher binding affinity compared to the compounds from ASR and FTB. In addition, some non-normal distribution was found. It is interesting to note that, for the targets except T03, T10, T11 and T12, one of the outlier points was the minimum binding score between the compounds and targets. Furthermore, the compounds against each target (except T11 and T20) with the lowest binding scores were all identified from the herb SFR.

### Three-dimensional structures of docked ligand–protein complexes

**Figure 3:**
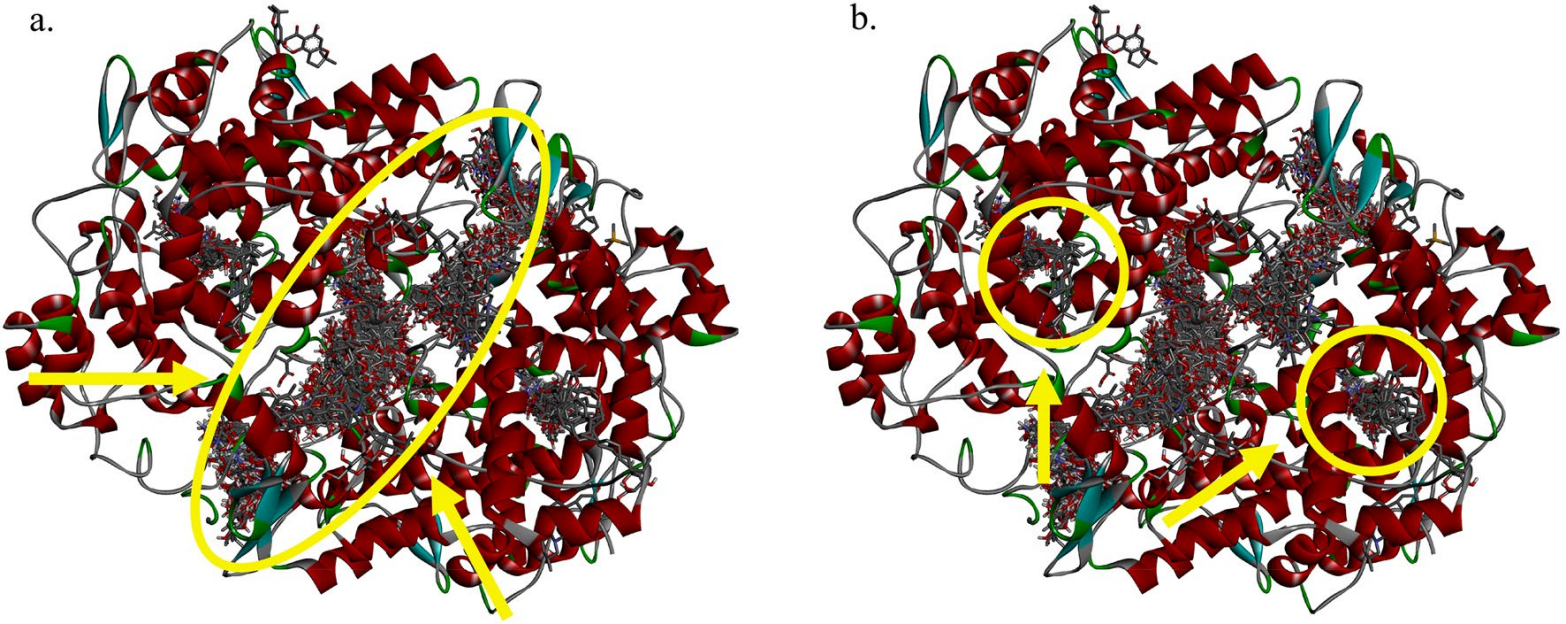
Docking pose interactions between all compounds and prostaglandin-endoperoxide synthase 2 (T03). (**a**) Primary binding sites of prostaglandin-endoperoxide synthase 2. (**b**) Active binding sites of prostaglandin-endoperoxide synthase 2.

T03 (PTGS2), which had the highest total binding affinity amongst all targets, plays an essential role in the process of cell motility, proliferation and anti-apoptosis^41^. It was a likely target for the DBKW herbal ligands examined and was, therefore, selected for analysis of its ligand–target interaction. This analysis enabled identification of T03 residues which play important roles in interactions with herbal ligands, enabling future mutagenesis experiments to verify the binding mechanisms proposed in this study. Figure 3 shows clusters of likely binding positions indicated by ligand-binding poses between all compounds from DBKW and T03.

For T03, 467 compounds were predicted to bind at the inter–monomer interface (Fig. 3a), involving compounds with the top five binding scores (KA090, ZC12, KB031, KA113 and KA091). These compounds are generally large molecules with a variety of structures, accounting for almost 75.2% of all herbal compounds. Nevertheless, these binding sites in T03 have not been investigated. Further studies could focus on the molecular mechanisms of these compounds for T03 via molecular dynamics simulations and experimental studies. These compounds, which come from all of the three herbs, may act to disrupt the function of T03 by preventing the formation of a functional dimer. In addition, clusters located directly at the two known active sites were focused on one at each monomer, since these were more likely to serve as competitive inhibitors of T03. In these active pockets, a total of 68 compounds were predicted to bind (Fig. 3b). It is interesting to find that most of the compounds that formed H-bonds with key active site residues were identified from the herb ASR. However, the boxplot (Fig. 2b) showed that compared to the compounds from FTB and ASR, more compounds from SFR had a higher binding affinity against T03. The reason behind the docking results needs to be further investigated.

Furthermore, direct binding of a ligand to the three key active site residues, including R120 (106), Y355 (341) and Y385 (371), is likely to enable effective inhibition of T03 by preventing these sidechains from performing their normal enzymatic function^41^. Therefore, analyses were focused on compounds predicted to form hydrogen bonding to these key active site residues. There were 21 compounds that formed hydrogen bonds (H-bonds) with one or more catalytic triad residues, including 19 compounds from ASR and 2 compounds from FTB (Table 3).

**Table 3:** Details of compounds from hydrogen bonds with one or more key active site residues.

No	Compound full name	Molecular weight	Key active site residues with H-bonds			Binding affinity
			R120 (106)	Y355 (341)	Y385 (371)	
**1. Compounds form hydrogen bonds with three catalytic triad residues**						
DC012	Azelaic acid	188.22	1	1	1	− 5.6
**2. Compounds form hydrogen bonds with two catalytic triad residues**						
DB019	Senkyunolide F	206.24	1	1	0	− 7.2
DA108	Tetradecanoic acid	228.37	1	1	0	− 6
DA175	5-Acetoxymethylfurfural	168.15	0	1	1	− 5.8
DA164	Trans,trans-2,4-Hexadienyl acetate	140.18	1	1	0	− 5.1
DA114	10-Undecenal	168.28	1	1	0	− 5
ZF04	Tyrosine	181.191	0	1	1	− 4.5
**3. Compounds form hydrogen bonds with one catalytic triad residue**						
DB004	E-Butylidenephthalide	188.22	0	0	1	− 7.4
DB005	Butylphthalide	190.24	0	0	1	− 7.4
DA012	4-Hydroxy-3-butylphthalide	206.24	0	0	1	− 7.4
DB024	3-Butylidenephthalide	188.22	0	0	1	− 7.3
DA053	2,4-Dimethylbenzaldehyde	134.17	0	0	1	− 6.5
DA153	Methyl linolenate	292.5	0	0	1	− 6.3
DA216	P-hydroxyacetophenone	136.15	0	0	1	− 5.9
DA145	E-10-pentadecenol	226.4	1	0	0	− 5.8
DA134	Benzaldehyde	106.12	0	0	1	− 5.3
DA173	2-Nonanone	142.24	0	0	1	− 5.2
DA165	2-Undecanone	170.29	0	0	1	− 5.1
DA196	6-Undecanone	170.29	0	0	1	− 5.1
ZF02	Leucine	131.175	0	0	1	− 4.9
DA172	4-Octanone	128.21	0	0	1	− 4.9

**Figure 4:**
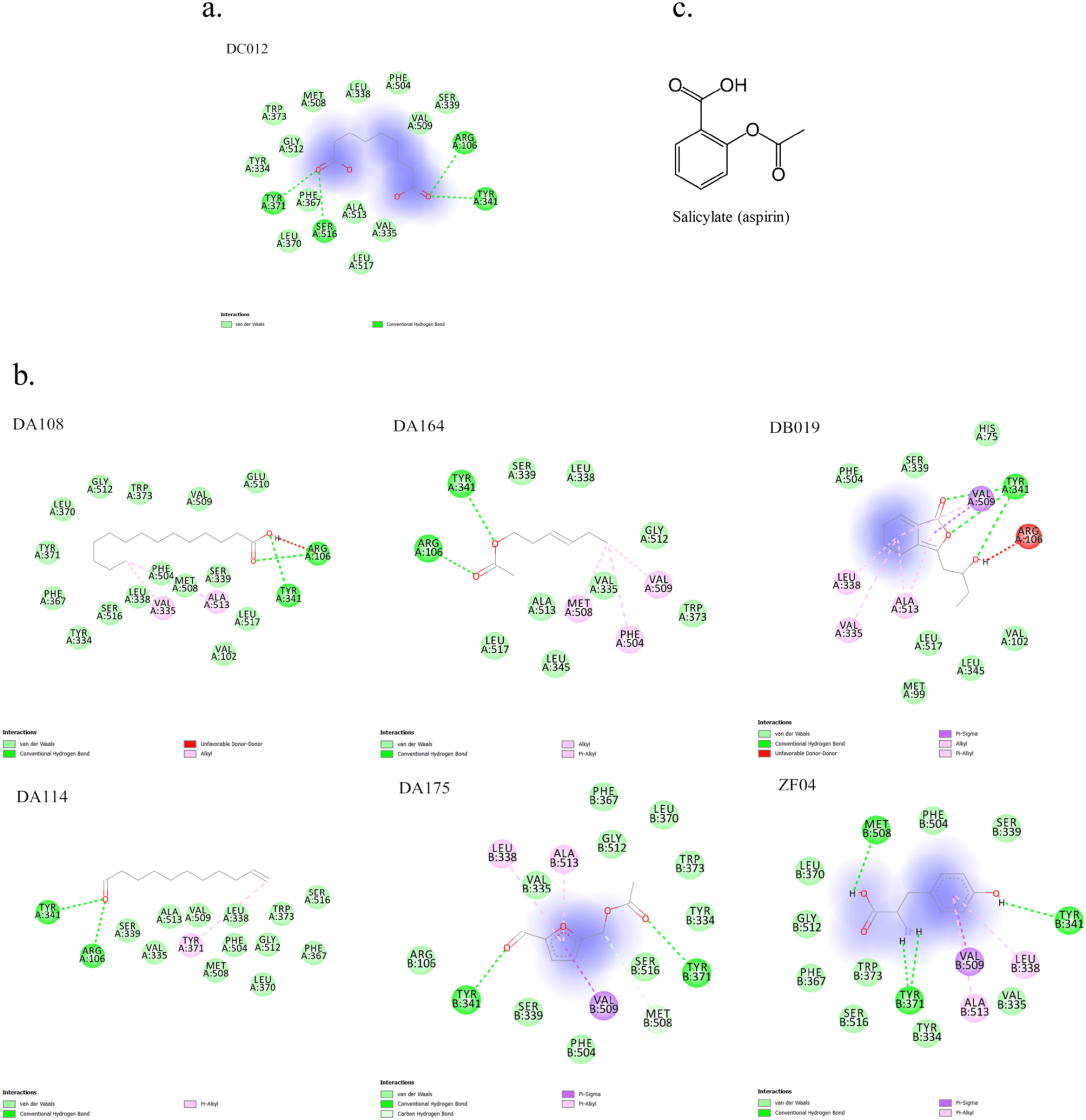
Ligand–target interactions for compounds forming hydrogen bonds with catalytic triad residues of prostaglandin-endoperoxide synthase 2. (**a**) Compounds forming hydrogen bonds with three catalytic triad residues. (**b**) Compounds forming hydrogen bonds with two catalytic triad residues. (**c**) Structure of inhibitor of prostaglandin-endoperoxide synthase 2, salicylate (aspirin). *DA108* tetradecanoic acid, *DA114* 10-undecenal, *DA164* trans,trans-2,4-hexadienyl acetate, *DA175* 5-acetoxymethylfurfural, *DB019* senkyunolide F, *DC012* azelaic acid, *ZF04* tyrosine.

Only 1 compound (azelaic acid (DC012)) was predicted to form H-bonds with all three catalytic triad residues (Fig. 4a), whereas 7 compounds formed H-bonds with 2 residues (Fig. 4b) and 14 compounds formed H-bonds with one of the key active site residues (Supplementary Fig. S6 online). DC012 contains a long hydrophilic chain with two carboxyl groups where the H-bonds were found connecting to the catalytic triad residues. For compounds forming H-bonds with two residues, all compounds were found to form bonds to residue Y355 (341), four compounds to residue R120 (106) and three compounds to residue Y385 (371). For the structures of these seven compounds, three of them contained long carbon chain structures (DA108, DA114 and DA164) and three of them contained aromatic rings (DA175, DB019 and ZF04). Three compounds formed H-bonds between their ester groups and residues (DA164, DA175 and DB019), two compounds formed H-bonds with surroundings via their aldehyde groups (DA114 and DA175), two compounds via hydroxyl groups (DB019 and F04), one compound via carboxyl group (DA108) and one compound via its amidogen group (ZF04). Lastly, 14 compounds formed H-bonds with only one of the key active site residues. Seven of them contained aromatic rings (DA012, DA053, DA134, DA216, DB004, DB005 and DA024) and the others contained long carbon chain structures (DA145, DA153, DA165, DA172, DA173, DA196 and ZF02). Furthermore, 13 compounds were found to form H-bonding to residue Y385 (371), 1 compound to residue R120 (106) and none to residue Y355 (341). In seven compounds, including DA053, DA134, DA165, DA172, DA173, DA196 and DA216, H-bonds were found between aldehyde groups and the surrounding residues. Five compounds, including DA012, DA153, DB004, DB005 and DB024, formed H-bonds with surrounding residues via their ester groups. In addition, H-bonds were identified between hydroxyl groups in DA145 and R120 (106) and between carboxyl groups in ZF02 and Y385 (371).

Comparing the above structures with the known inhibitor of PTGS2, salicylate (aspirin), which has an aromatic ring with a carboxyl and an ester group, some similarities could be identified (Fig. 4c)^42^. The structures of 10 compounds had aromatic rings, including DA012, DA053, DA134, DA175, DA216, DB004, DB005, DB019, DB024 and ZF04. A total of eight compounds had ester groups (DA012, DA153, DA164, DA175, DB004, DB005, DB019 and DB024) and three compounds involved carboxyl (DA108, DC012 and ZF02).

On the other hand, the top five binding affinity compounds of PTGS2 were KA090, ZC12, KB031, KA113 and KA091, whereas in the active binding sites, the top five binding affinity compounds were KA120, DA064, ZC07, DA084 and DA012. These compounds come from all three herbs of the formula, indicating that the effects of the compounds from the herbal formula may be superior to those of the compounds from any single herb. However, binding affinity values alone may not be fully accurate as an indicator of potential biological activity, since they can have errors of up to 2 kcal/mol^43^. Therefore, an inspection of the number of strong non-covalent interactions between ligands and binding sites should also be used to predict potential bio-activities of herbal compounds, a general approach previously employed in analyses of molecular docking results^44,45^. H-bonds are the strongest non-covalent interactions and therefore, the total number of H-bonds formed between ligands and key active site residues can be used to predict the extent to which a ligand may act as an effective inhibitor of PTGS2. Moreover, compared to the structure of the known inhibitor of PTGS2, within the compounds that form H-bonds with key active site residues, these compounds are predicted to be inhibitors of T03, including DC012, DA175, DB019, ZF04, DA012, DB004, DB005 and DB024. Additionally, seven compounds were predicted as inhibitors of PTGS2 with moderate probability, including DA053, DA108, DA134, DA153, DA164, DA175 and ZF02. Although some of the compounds mentioned above were reported to have pharmacological activities, such as azelaic acid (DC012)^46^, butylphthalide (DB005)^47^ and P-hydroxyacetophenone (DA216)^48^, none of these 15 compounds were reported as an inhibitor of T03. Thus, these compounds from DBKW are worthy of further examination for their possible novel inhibitory activity against T03.

### Biological pathways prediction

There are three signalling pathways in the top 10 KEGG pathways (pathways in cancer, p53 signalling pathway and NF-κB signalling pathway) that are highly associated with cancers, incluing PCa^49^ (Fig. 1c). A total of eight target proteins (T01 to T07, and T10) are involved in the pathway relevant to the occurrence and development of cancers^50^. Molecular docking prediction indicated that the total binding score of these targets ranging from − 3628.0 to − 4877.5 kcal/mol. Five of them (T02, T03, T05, T06 and T07) have a high total binding affinity (< − 4000.0 kcal/mol), involving the top total binding affinity protein (PTGS2), implying that DBKW may act on this pathway. In addition, five targets were clustered in to the p53 signalling pathway including T01, T02, T05, T06 and T07 with a range of − 3773.0 to − 4704.0 kcal/mol total binding affinity. In this pathway, TP53, which has a total binding affinity of − 3773.0 kcal/mol, is a transcriptional activator of TP53-regulated targets functioning for the cell cycle arrest, cellular senescence and apoptosis^51–53^. Moreover, other TP53-regulated targets have a close relationship with repairing damaged DNA in human body, as they can strengthen or weaken the activities of TP53^54^. All the four enriched TP53-regulated targets have a high total binding affinity (< − 4000.0 kcal/mol). Thus, this pathway may also be one of the biological pathways that DBKW acts on. Lastly, recent studies reported that NF-κB may be strongly associated with the development of inflammation-induced cancer since it may stimulate tumour cell survival, invasion, metastasis and androgen deprivation therapy drug resistance^55^. Moreover, it has been hypothesised that the carcinogenesis effects induced by chronic inflammation may be reduced if the NF-κB signalling pathway is inhibited^56^. According to the present molecular docking results, the total binding affinities of the four targets in this group including T03, T05, T08 and T09 were from − 3619.5 to − 4877.5 kcal/mol. Furthermore, T03 and T05 have a high total binding affinity (< − 4000.0 kcal/mol), accounting for 50% of all annotated targets in this pathway, indicating that DBKW may target NF-κB signalling pathways, which may be a treatment strategy for inflammation-induced cancer^56^.

### Interaction network of formula, herbs, compounds and targets

**Figure 5:**
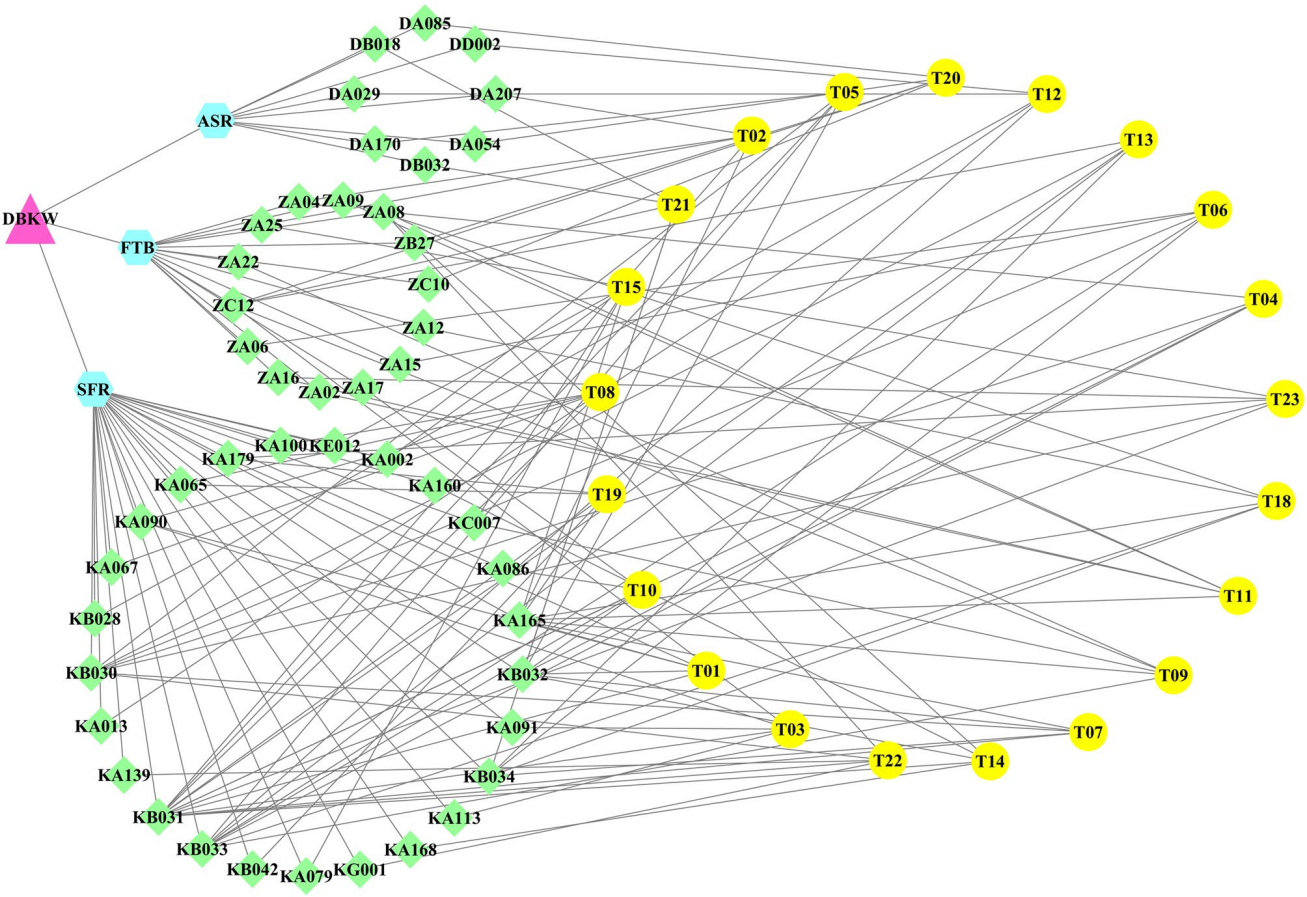
Network of the formula, herbs, chemical compounds and targets. *ASR* Angelicae Sinensis Radix, *DBKW* Danggui Beimu Kushen Wan, *FTB* Fritillariae Thunbergii Bulbus, *SFR* Sophorae Flavescentis Radix. For  corresponding compound names, refer to Supplementary Tables S1 to S3 online; for corresponding target names, refer to Table 1.

A total of 47 compounds were selected, including 8 compounds from ASR, 14 compounds from FTB and 25 compounds from SFR. A network was generated to demonstrate the relationship between the formula, herbs, the top five compounds with the highest binding affinity and the 21 targets (Fig. 5). Figure 5 shows that SFR had the highest number of high binding affinity compounds (25 compounds), which interacted with the highest number of targets (20 targets excluding CYP19A1 (T20)), followed by FTB, which had 14 compounds interacting with 13 targets. For ASR, eight compounds were identified with high binding affinity when binding with five targets. Amongst all of the 47 high binding affinity compounds, 15 compounds, including KB031, KA165, KB033, KB030, KB032, KB034, ZC12, ZA08, KA090, KA179, KA065, KC007, ZA09, ZA16 and ZA25, interacted with more than one target. Flavesine B (KB031) interacted with the most targets (13 targets), including T01, T02, T03, T04, T05, T07, T08, T10, T13, T14, T19, T22 and T23.

The network indicates that high-binding-affinity compounds may have potential biological activities with the targets, although most of the compounds were from SFR. Current research indicates that combining active compounds produces additional or even synergistic effects which are superior to those of a single compound, since concurrent and selective interactions may occur with multiple targets of a disease^57,58^. Although the molecular docking results provided a comprehensive overview of the possible mechanisms of action of DBKW for PCa targets, these docking results should be validated in further experimental studies. Furthermore, there are no existing studies relevant to the mechanisms of action of the top five high-binding-affinity compounds for the corresponding targets. Thus, these compounds identified from DBKW may be novel scaffolds for the management of PCa.

### Strengths and limitations

Although CHM has been widely used in China and Western countries, its clinical applications are always questionable due to a lack of scientific evidence. This study has combined knowledge bases (literature and database searches) and computational approaches to form a more complete picture of the physiological pathways through which the herbal formula may act to treat PCa, thereby, enhancing the chance of producing effective targeted therapy. This is an innovative multidisciplinary approach to the identification of potential key bioactive natural products in a complex mixture of DBKW that will lay the groundwork for future in vitro and pre-clinical studies in cancer animal models. This creative multidisciplinary approach focused on the ‘multi-target, multi-compounds’ approach, which overcomes the weaknesses of the traditional ‘one target, one drug’ approach. The interactions between the herbal formula, its ingredients with multiple compounds and multiple targets can be clearly illustrated through the establishment of their network. Therefore, the scientific evidence developed through this process can be used to explain the mechanisms of action of the herbal formula. This methodology can also be adapted to explore other natural products for other diseases.

Nonetheless, limitations in this study could not be avoided. The successful development of a drug relies critically on understanding its pharmacokinetics and potential toxicity, as well as its propensity to be falsely identified as bioactive. For the compounds, all compounds with known structures were selected for docking and may have included some toxic chemicals. However, the primary purpose of this project was to identify as many potential compounds as possible for the targets first. If the potential compounds are toxic, further analyses need to be performed in future studies to identify safe doses for humans or to manipulate the structures of the compounds in order to reduce their toxicity. Secondly, language barriers can occur during a text-mining approach. In this project, only articles published in English or Chinese were included. However, articles with high-quality and well-designed studies written in other languages, such as Japanese and Korean, may have been ignored. It is recommended that future review studies include an investigator with other Asian language backgrounds in the review team in order to facilitate examination of non-English and non-Chinese literature. Thirdly, the current project did not involve experimental studies (such as in vitro and in vivo) to validate the in silico results. The results from the current project need to be interpreted with caution as a compound may show a strong binding in docking studies and even in in vitro experiments, however, it may not exert biological activities in animal studies. Thus, future studies should perform in vitro and in vivo experiments to validate the computational results. Lastly, there is always a limitation to extrapolate in silico findings to in vivo or clinical situations without consideration of other factors, including doses, pharmacokinetics and patients’ conditions. Those factors should also be investigated and confirmed in further research.

## Conclusions

DBKW is a classical herbal formula developed 1800 years ago and it has been widely used for treating difficulty in urination involved in PCa in modern times. Molecular docking indicated that compounds from DBKW may interact with 21 targets associated with PCa. The binding patterns showed that a relatively small number of tight-binding components from DBKW were predicted to interact strongly and selectively with three targets (T03, T23 and T21), especially for T03 (PTGS2), at some specific, highly attractive binding positions. Fifteen DBKW compounds (DC012, DA175, DB019, ZF04, DA012, DB004, DB005, DB024, DA053, DA108, DA134, DA153, DA164, DA175 and ZF02) were predicted as inhibitors of PTGS2. Three signalling pathways including pathways in cancer, p53 signalling pathway and NF-κB signalling pathway in the top 10 KEGG pathways were identified and that may be highly associated with cancers, involving PCa. Network analysis showed that DBKW contains multi-targeting agents that could act on more than one pathway of PCa at the same time. Although molecular docking provided an initial insight into the possible mechanisms of action of DBKW for PCa, the exact interactions between promising compounds, corresponding targets and diverse pathways need to be thoroughly investigated further. The stability of the ligand–protein poses predicted in the current study could be assessed using molecular dynamics simulations in the future. A multidisciplinary network-based pharmacological study of DBKW for PCa, including in silico, in vitro and in vivo studies, is needed, as this would systematically explore the relationship across the formula, herbs, chemical compounds, targets and pathways involved in PCa. Moreover, pharmacokinetic and toxicity studies, and high-quality and well-designed RCTs, are recommended in the future to comprehensively investigate the effects and safety of DBKW for the management of PCa.

## Methods

### Identification of compounds from DBKW’s ingredients

Chemical compounds identified from DBKW’s ingredients were obtained from the published literature, which provided the phytochemical and pharmacodynamic properties of DBKW from modern experimental studies^28^.

### Acquisition of structures of identified compounds

Each of the identified compound was searched in the PubChem database (https://pubchem.ncbi.nlm.nih.gov) for its PubChem CID/SID number, 3D structures and physicochemical properties. Each molecular structure was obtained in a standard SMILES (SDF file) format. Molecular structures that could not be found in PubChem were drawn manually using the software ChemDraw 18.2. All molecular structures were converted into the conventional protein structure PDB file format using Chem 3D 18.2. Chemical structures were checked and corrected using the software where necessary during the conversion.

### Identification of potential targets for PCa

#### Literature search

We identified potential drug targets of DBKW from the included article in our published thesis, as the thesis has included all pharmacological studies of DBKW in 21 electronic databases^28^. We identified drug targets in studies if the original three-herb DBKW formula was utilised as the intervention and focused on targets for cancers in the study. Considering close relationship between PCa and chronic prostatitis as described before, we also identified targets from the studies relevant to chronic prostatitis.

Then, one researcher (HL) screened the included studies to identify possible drug targets and extracted the data into a predesigned Excel template. The second researcher (AY) double checked the data. When any discrepancies between the two researchers occurred, a discussion with the third party (AH) was conducted. Characteristics of the candidate drug targets of DBKW were descriptively summarised.

#### Approved drugs for PCa

The 2019 National Comprehensive Cancer Network Clinical Practice Guidelines in Oncology-Prostate Cancer was searched to identify currently approved drugs for PCa^12^. The guideline was electronically screened to identify the names of all drugs recommended for PCa. Subsequently, the known drug targets were retrieved from the DrugBank database (www.drugbank.ca) on 18 August 2019, using drug names as keywords. The data was checked by a researcher (AY). Discussion with the third party (AH) was performed if any disagreement between the two researchers occurred. The treatment methods, drug names and their drug targets were descriptively summarised.

#### KEGG enrichment of selected target proteins for PCa

Since it is significant for drug discovery to thoroughly understand the biological functions and possible pathways of multiple targets, KEGG enrichment was performed^59^. KEGG enrichment aimed to investigate potential biological pathways of the candidate proteins^50,60–62^ and we used the Enrichr database (https://amp.pharm.mssm.edu/Enrichr) to perform the enrichment. The Enrichr database is a public database containing more than 180000 gene sets based on 102 public sources and it provides more systematic annotated results than other commonly used databases, such as MSigDB^60^. To confirm the enrichment results were statistically significant, we set ‘*p* < 0.05’ in the database. We also selected the top ten KEGG annotated pathways which were ranked by their corresponding *p*-values to generate a network using Cytoscape (v3.7.2)^63^, as this network could distinctly present the connection between targets and significant pathways.

#### Selection of candidate targets for subsequent computational analyses

We identified candidate targets of DBKW from literature search. There were four groups of identified targets: targets identified from the studies on PCa were categorised as Group A; targets identified from the studies on cancers except for PCa were defined as Group B; targets identified from the studies on chronic prostatitis were classified as Group C; and targets from currently approved drugs for PCa were regarded as Group D. Additionally, targets listed under the category of ‘prostate carcinoma’ in the Open Targets database (www.opentargets.org) were defined as Group E, which were used as a reference target list to compare to the targets from the four groups (Groups A, B, C and D) respectively. The Open Targets database could not only connect drug targets to diseases, but also comprehensively identify and prioritise targets based on multi-year and large-scale human genetics and genomics data from various of public data sources^39^. We selected the overlap targets, which were identified from the cross-comparison approach, for subsequent in silico analyses.

In order to systematically understand the biological functions of multiple candidate targets and their potential interactions^64^, we used the STRING database (https://string-db.org), a publicly available and accessible database, to analyse the PPI networks of the candidate targets^65^. The selected candidate targets were input and searched using the Homo Sapiens program. We set the network edges to ‘Confidence’ to present the strength of data support, and defined the interaction score to ‘above 0.400’ to identify the results with medium confidence. We included targets if they demonstrated PPI for subsequent analyses. For targets which did not interact with each other, we excluded them.

### Acquisition of structures of the selected targets

The Uniprot ID and PDB ID of the 28 proteins were searched and obtained from the Uniprot database (www.uniprot.org). A basic local alignment search tool (BLAST) search was performed using the online BLAST server (https://blast.ncbi.nlm.nih.gov/Blast.cgi) to identify the most appropriate protein sequences^66^. The structures of identified protein sequences were downloaded from the RCSB PDB Protein Data Bank (www.rcsb.org) in PDB file format and then examined and compared using the protein visualisation and analysis software VMD. For proteins from the PDB with missing loop segments, homology modelling was employed using the SWISS-MODEL server (www.expasy.org/swissmodel) to repair the 3D structures of these proteins^67^. Protein structures retrieved from the RCSB database or predicted via homology modelling were pre-processed using PyRx for subsequent computational docking studies.

### Molecular docking between compounds from DBKW and targets for PCa

Molecular docking is a computational technique that tests various orientations and conformations of a ligand to identify possible binding sites of targets and to provide approximate estimates of ligand-binding-affinity values^68^. Interactions between DBKW compounds and targets for PCa were predicted using the automated docking software PyRx (v0.8) and AutoDock Vina (v1.1.2). The docking software AutoDock Vina was utilised in this project to conduct molecular docking^69^. All protein and compound files for molecular docking were prepared by the docking GUI frontend PyRx, which was also employed to produce docking parameter input files. All protein and compound PDBQT files were prepared by PyRx based on their corresponding PDB files. The ‘Maximise’ option in PyRx was used to define the docking boxes around the targets, since it could ensure the availability of the entire protein surface and accessible interior pockets for potential binding of ligands during ‘blind’ docking. The default exhaustiveness value of 8 was set for all molecular dockings. The dockings were performed by specifying fixed structures for protein receptors, whereas the ligands were semi-rigid with full torsional flexibility. The Intel Xeon Sandy Bridge 2.6 GHz Broadwell nodes of the high-performance computing cluster located at the National Computational Infrastructure (Canberra, Australia) was used to conduct Autodock Vina calculations.

### Visualisation of 3D docking positions and 2D ligand–receptor interaction diagrams

Targets with the highest predicted total binding affinity to the herbal compounds were selected for analysis of 3D structures and 2D visualisations of ligand–residue interactions at their respective docking positions. Ligand–residue contact plots were generated using the software Discovery Studio Visualizer 2019. The known active binding sites and the key active binding site residues were searched in the RCSB Protein Data Bank database. Compounds located in the active binding sites were selected to identify the number of hydrogen bonds formed with the key active site residues. The structures of the compounds were descriptively summarised and compared to the known ligand of the target.

### Network model analyses between formula, herbs, chemical compounds and targets

Considering the difference in binding affinity within each compound against various targets, the top five compounds with the highest binding affinity values to each target were selected for further investigation, since these compounds were the most likely to have physiological importance. The top five compounds with highest binding affinity were selected to generate a network using Cytoscape (v3.7.2), to demonstrate the relationships across the formula, herbs, chemical compounds and targets. The target and the herb were considered to possess a strong connection if the chemical components from the herb had an optimal binding affinity beyond a prescribed threshold which was determined based on the range of predicted binding energies obtained for the entire herbal library with the target.

## Supplementary Information

Supplementary information.
