# Supplementary material for: Herb-target virtual screening and network pharmacology for prediction of molecular mechanism of Danggui Beimu Kushen Wan for prostate cancer
Source: Sci Rep. 2021 Mar 23;11:6656. doi: 10.1038/s41598-021-86141-1 (PMC7988104; doi:10.1038/s41598-021-86141-1)
Supplement: Supplementary file 1 — Supplementary information. [file 41598_2021_86141_MOESM1_ESM.pdf]

Supplementary flies for

Herb−target virtual screening and network pharmacology for prediction of molecular mechanism of Danggui Beimu Kushen Wan for prostate cancer

Hong Li[1], Andrew Hung[2], Angela Wei Hong Yang[1]*

[1] School of Health and Biomedical Sciences, RMIT University, Bundoora, Victoria 3083, Australia

[2] School of Science, RMIT University, Melbourne, Victoria 3000, Australia

*Corresponding author:
Dr Angela Wei Hong Yang
Discipline of Chinese Medicine
School of Health and Biomedical Sciences
RMIT University
PO Box 71
Bundoora, Victoria 3083
Australia
Phone: 61-3-9925 7175
Fax: 61-3-9925 7178
Email: angela.yang@rmit.edu.au

Table of Contents

Supplementary Figures 3

Supplementary Figure S1. Molecular structures of 18 compounds that could not be found in PubChem from Angelicae Sinensis Radix. 3

Supplementary Figure S2. Molecular structures of 15 compounds of Fritillariae Thunbergii Bulbus that could not be found in PubChem. 5

Supplementary Figure S3. Molecular Structures of 94 Compounds that could not be found in PubChem from Sophorae Flavescentis Radix. 6

Supplementary Figure S4. Venn diagram summary of chemical compounds identified from Danggui Beimu Kushen Wan. 13

Supplementary Figure S5. Flow chart of selection process for chemical compounds from Danggui Beimu Kushen Wan for molecular docking. 14

Supplementary Figure S6. Ligand–target interactions for compounds forming hydrogen bonds with one catalytic triad residue. 15

Supplementary Tables 22

Supplementary Table S1. Summary of herbal compounds identified from Danggui Beimu KushenWan's ingredients. 22

Supplementary Table S2. Details of identified anti-cancer drugs from 2019 National Comprehensive Cancer Network Clinical Practice Guideline-Prostate Cancer and their drug targets 60

Supplementary Table S3. Summary of Kyoto Encyclopedia of Genes and Genomes enrichment of the included targets. 61

Supplementary Table S4. Details of docking results between 621 natural compounds from Danggui Beimu Kushen Wan and 21 targets for prostate cancer (kcal/mol). 65

References 87

Supplementary Figures

Supplementary Figure S1. Molecular structures of 18 compounds that could not be found in PubChem from Angelicae Sinensis Radix.

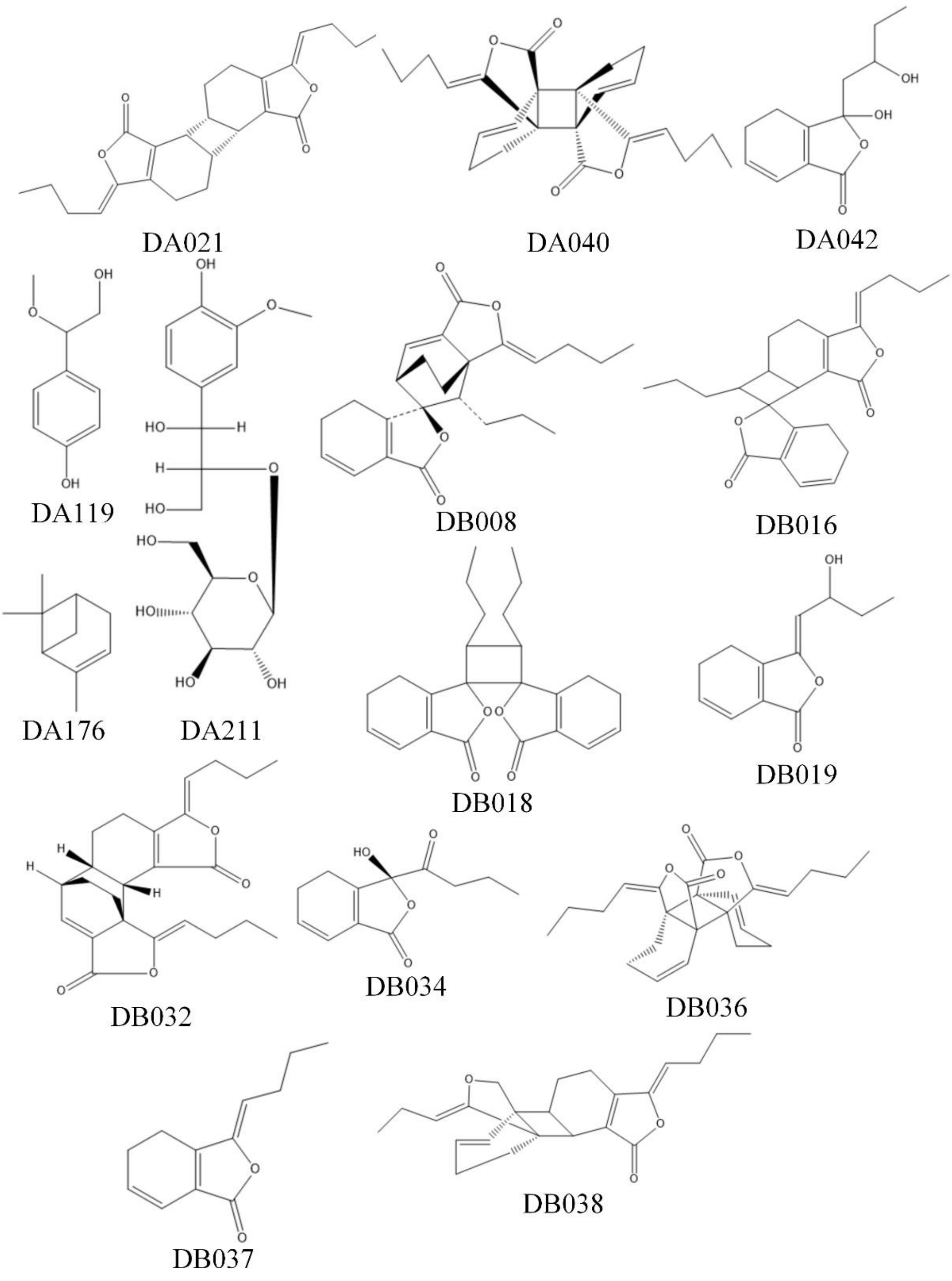

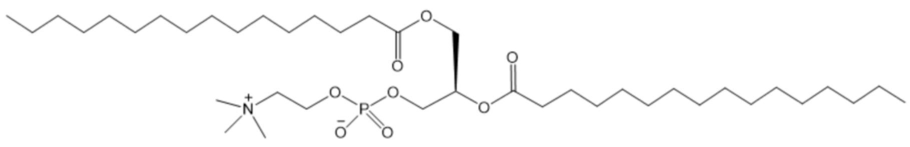

DE001

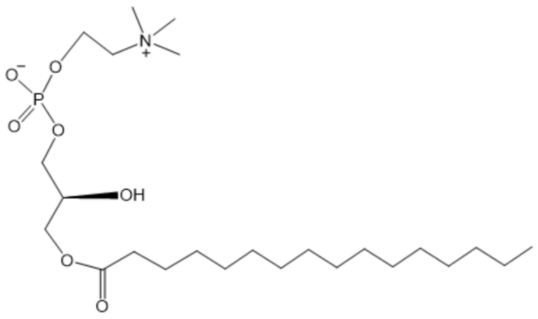

DE002

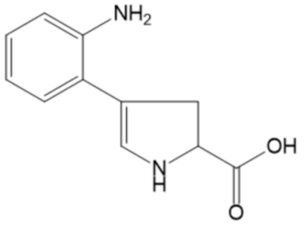

DI001

Supplementary Figure S2. Molecular structures of 15 compounds of Fritillariae Thunbergii Bulbus that could not be found in PubChem.

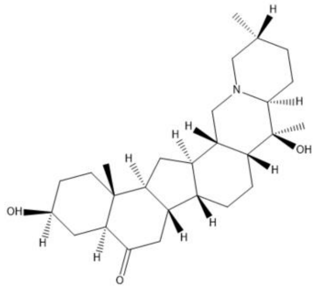

ZA04

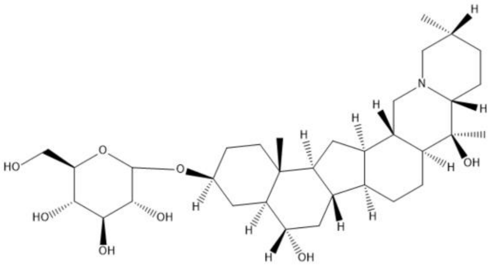

ZA11

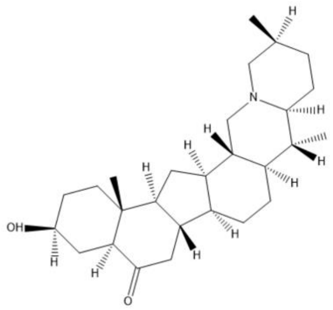

ZA16

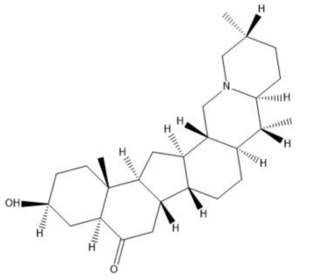

ZA17

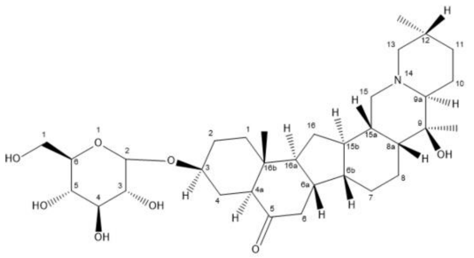

ZA21

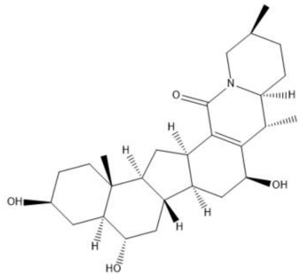

ZA23

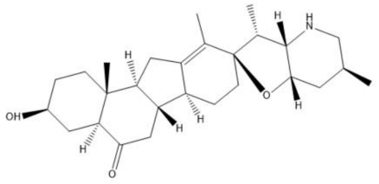

ZA24

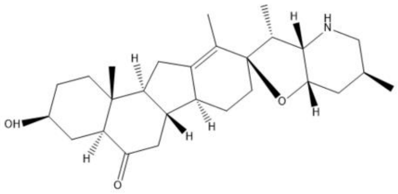

ZA25

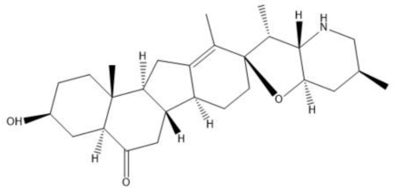

ZA26

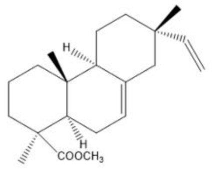

ZC02

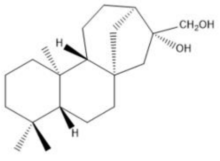

ZC03

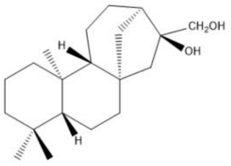

ZC04

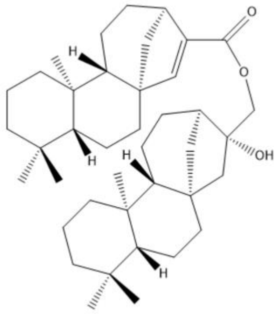

ZC12

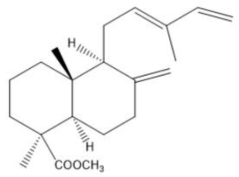

ZC09

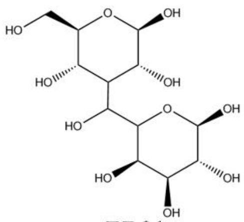

ZD01

Supplementary Figure S3. Molecular Structures of 94 Compounds that could not be found in PubChem from Sophorae Flavescentis Radix.

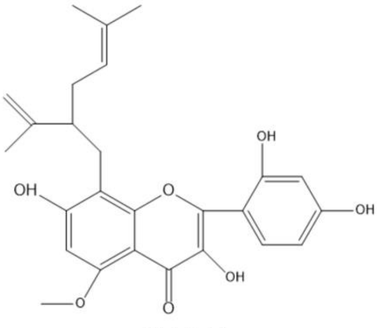

KA004

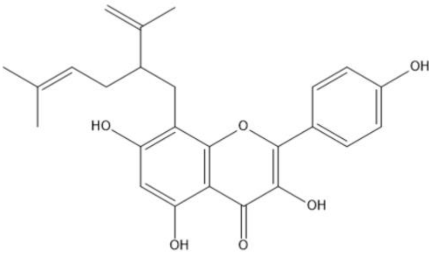

KA011

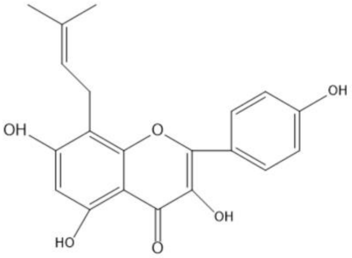

KA018

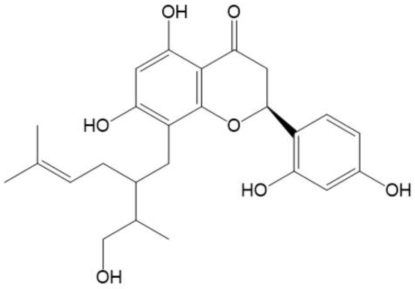

KA025

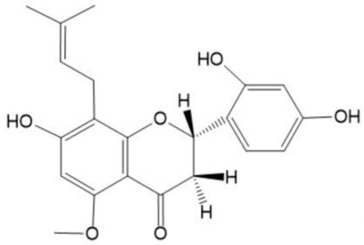

KA049

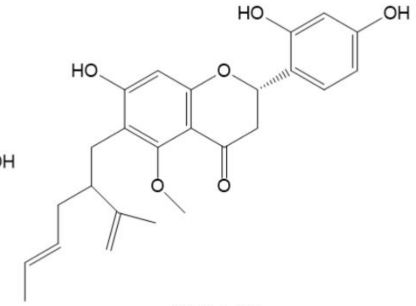

KA050

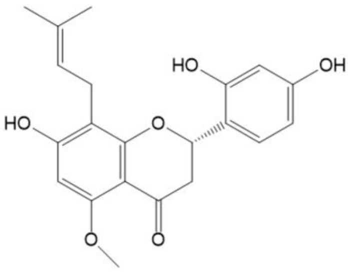

KA051

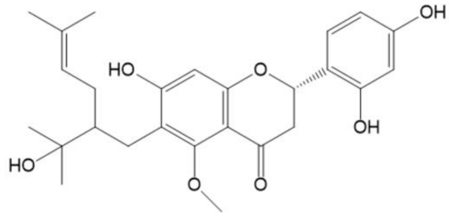

KA052

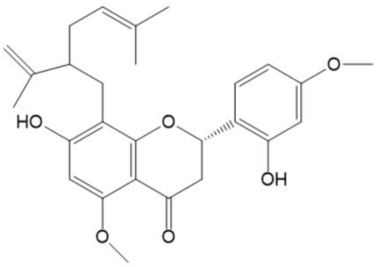

KA053

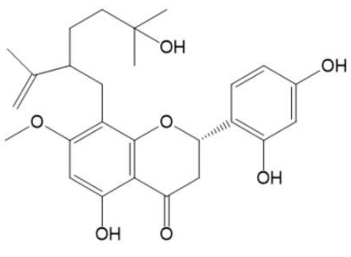

KA054

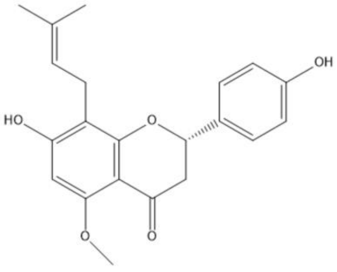

KA055

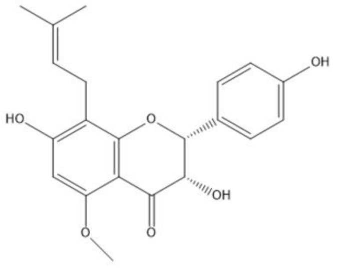

KA056

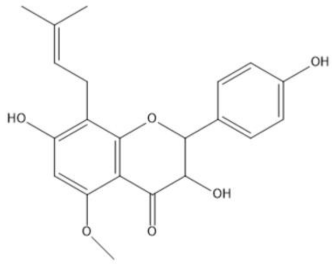

KA057

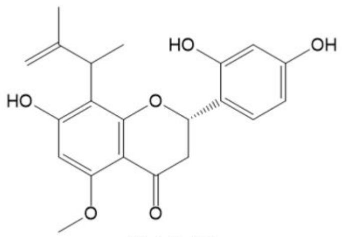

KA058

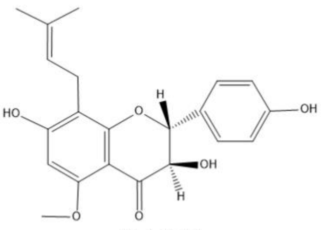

KA059

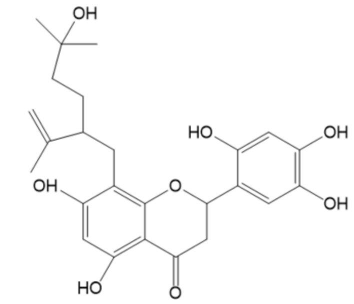

KA061

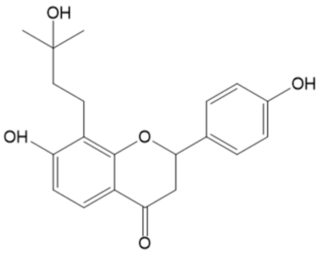

KA062

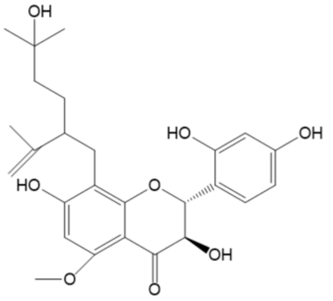

KA068

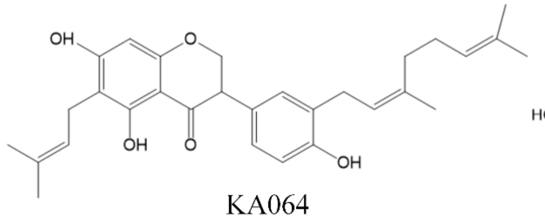

KA064

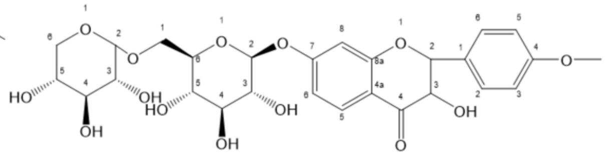

KA065

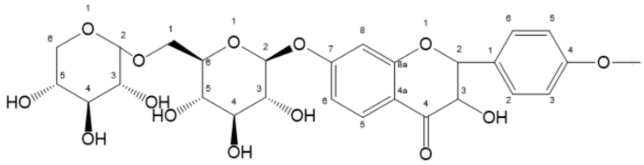

KA067

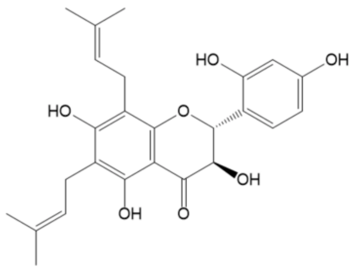

KA069

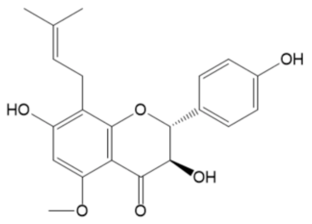

KA076

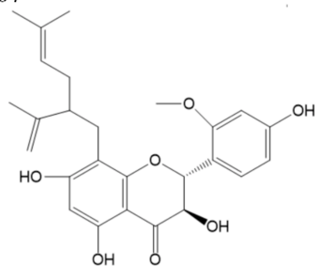

KA077

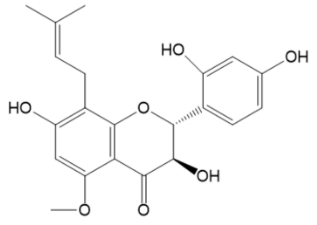

KA078

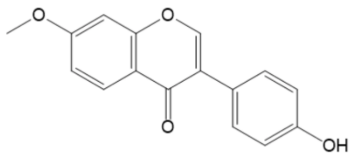

KA083

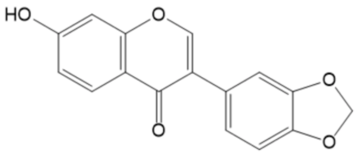

KA089

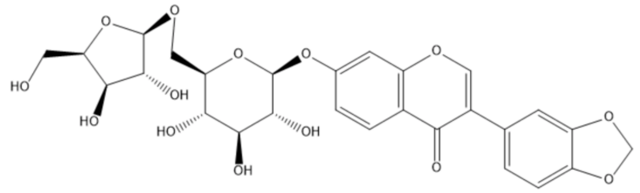

KA090

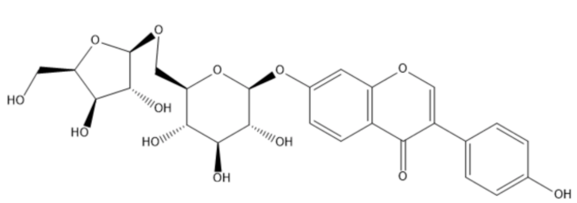

KA091

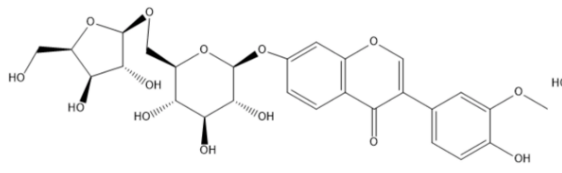

KA092

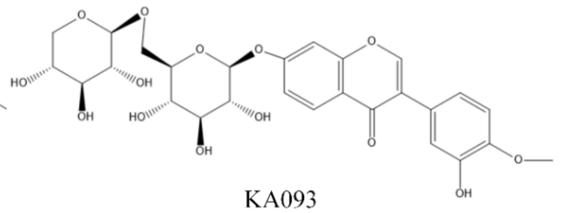

KA093

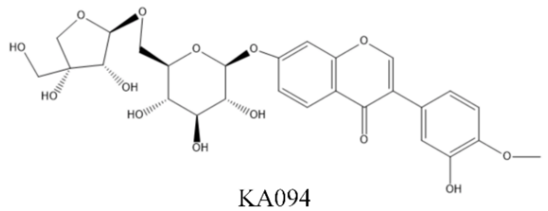

KA094

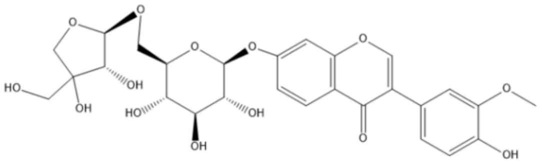

KA095

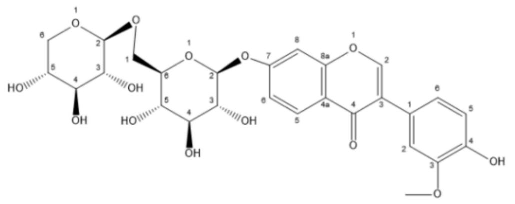

KA096

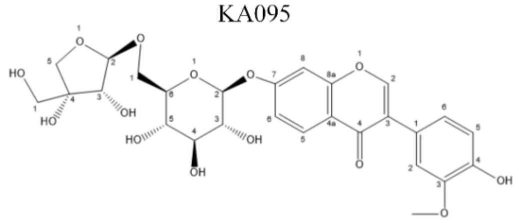

KA097

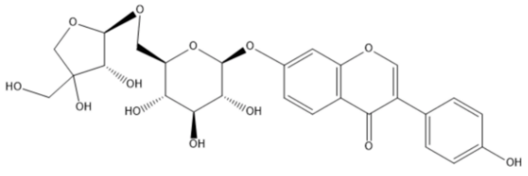

KA098

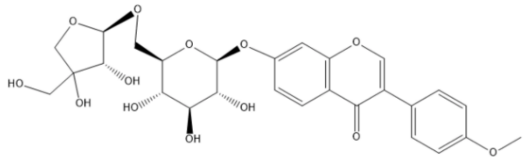

KA099

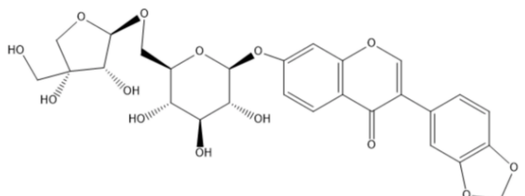

KA100

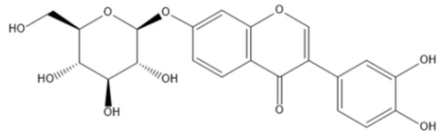

KA101

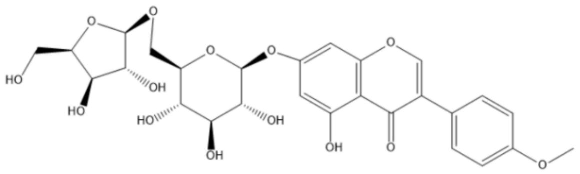

KA102

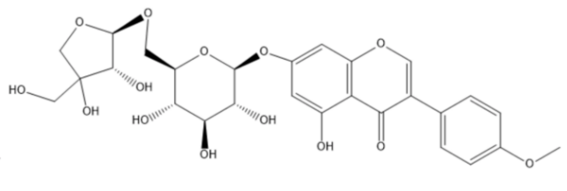

KA103

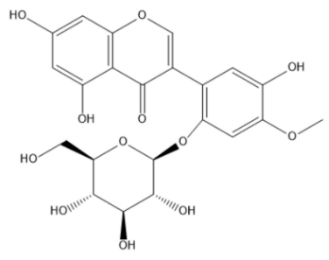

KA104

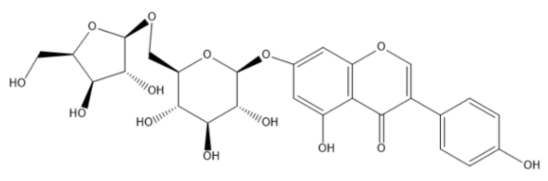

KA105

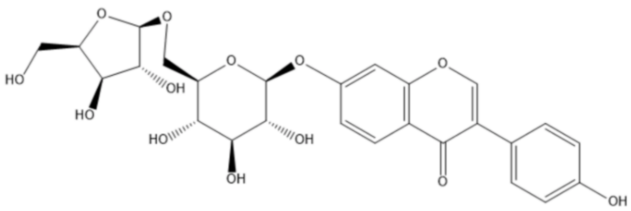

KA107

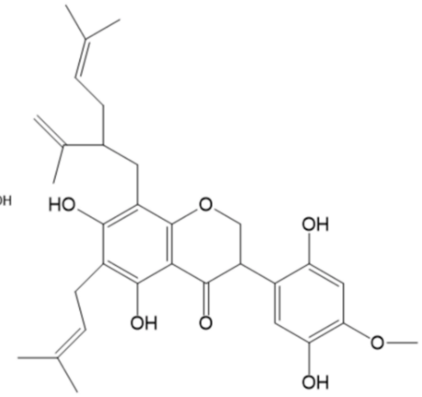

KA108

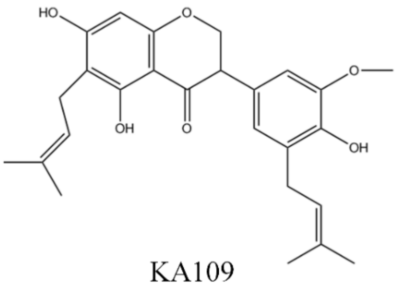

KA109

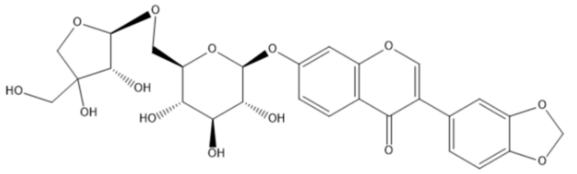

KA115

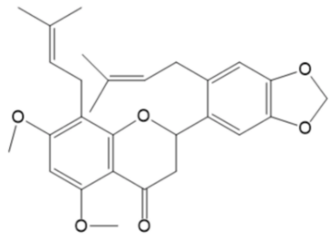

KA110

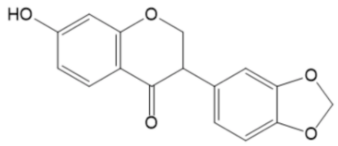

KA116

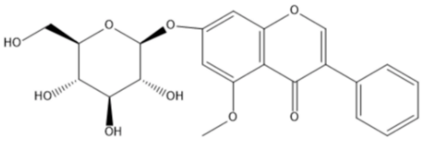

KA121

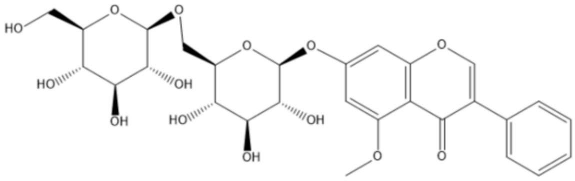

KA122

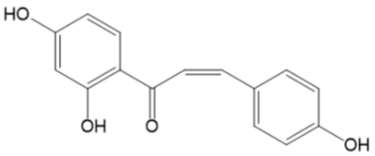

KA132

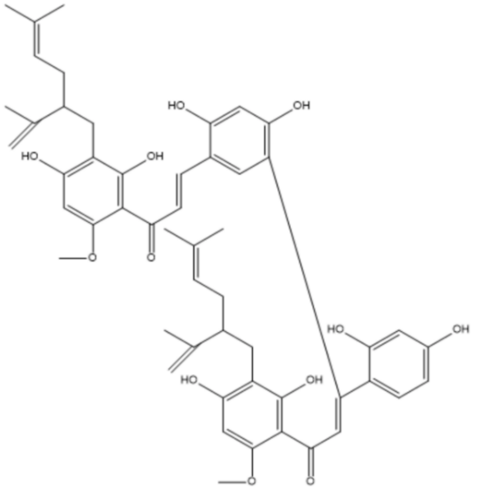

KA133

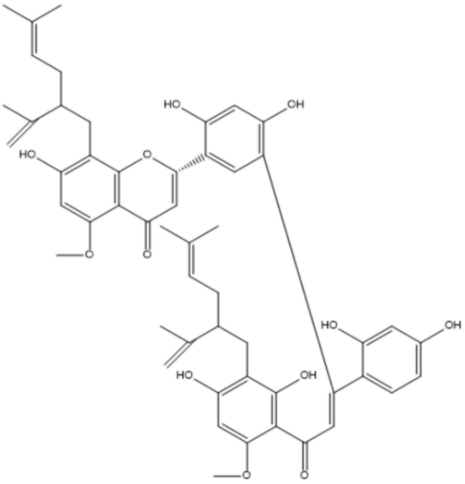

KA134

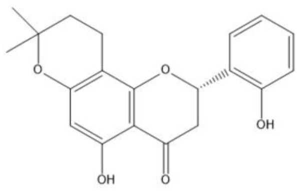

KA148

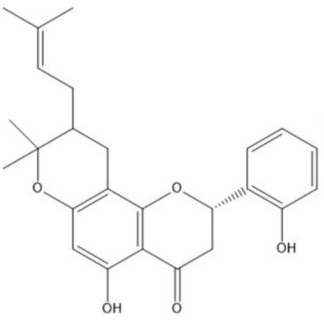

KA149

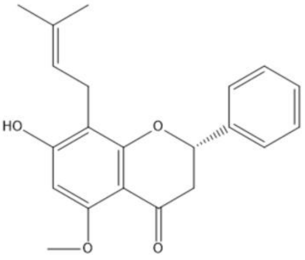

KA151

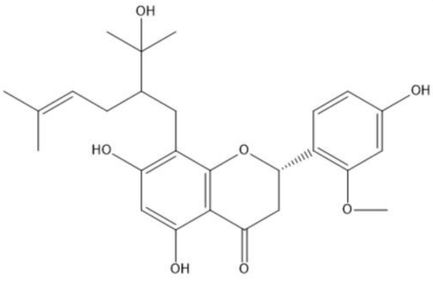

KA152

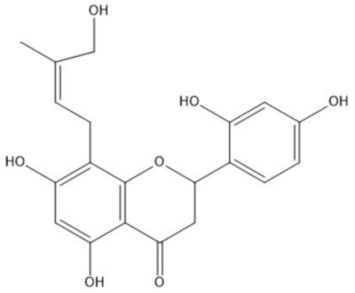

KA155

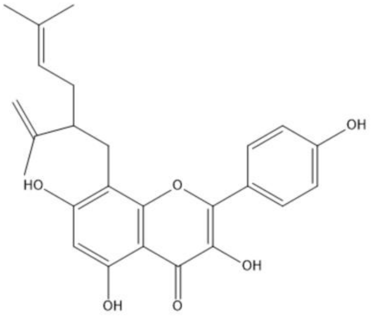

KA156

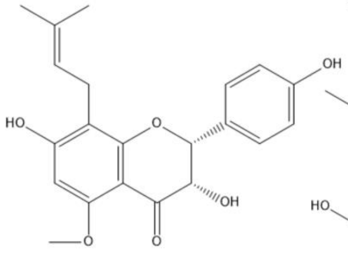

KA163

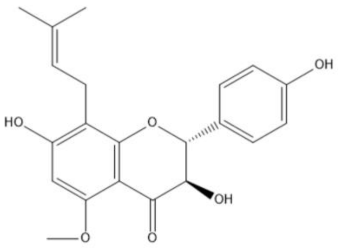

KA164

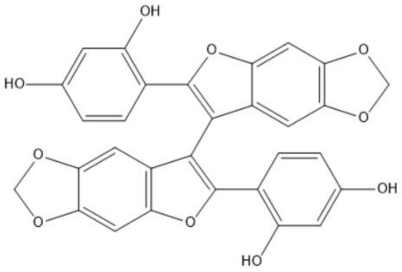

KA165

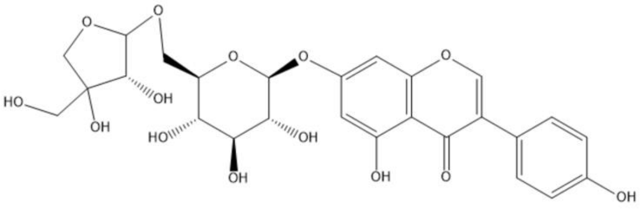

KA173

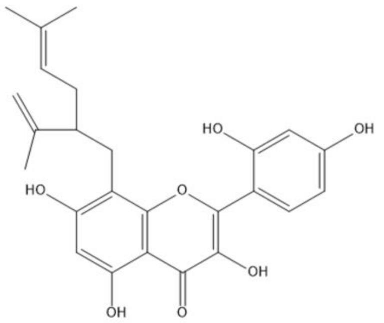

KA174

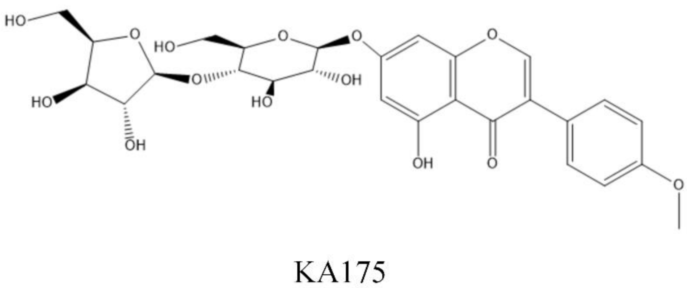

KA175

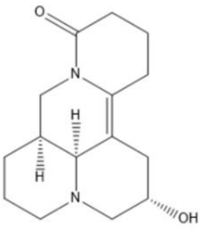
KB006

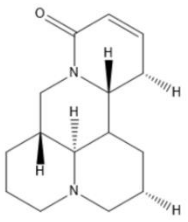
KB013

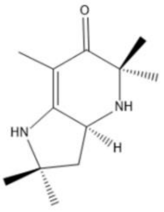
KB027

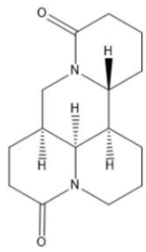
KB028

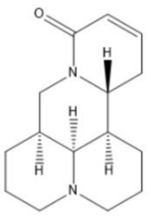
KB029

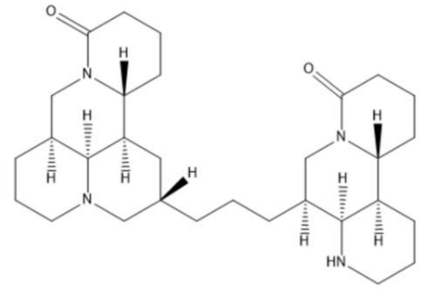
KB030

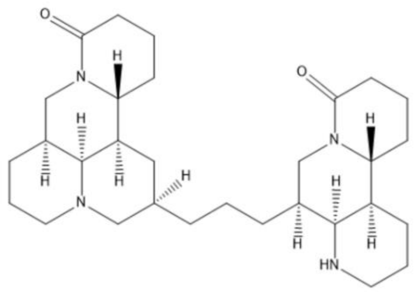
KB031

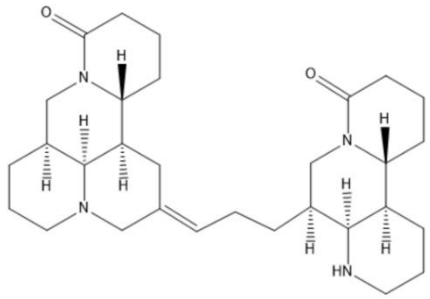
KB032

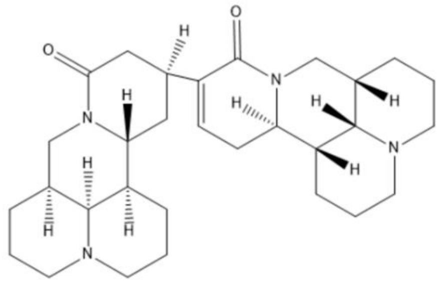
KB033

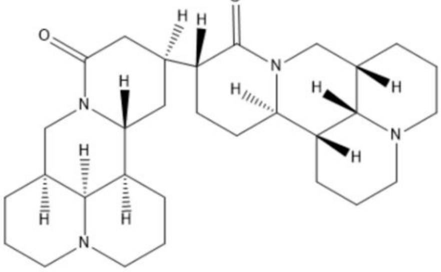
KB034

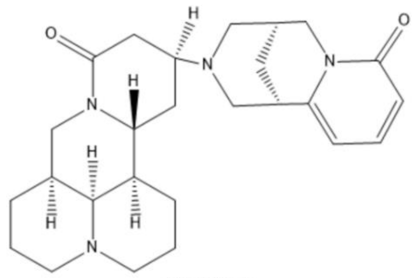
KB035

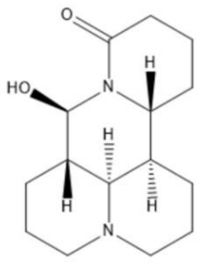
KB038

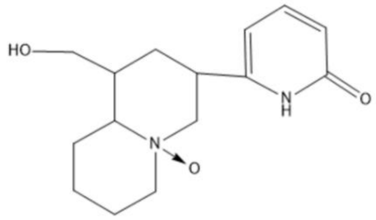
KB042

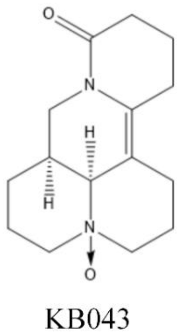
KB043

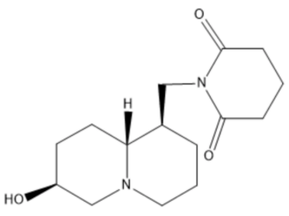

KB045

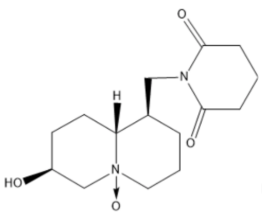

KB046

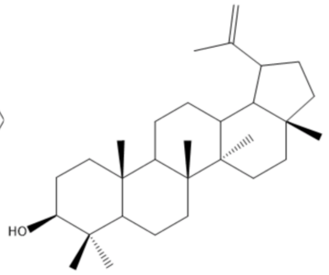

KC003

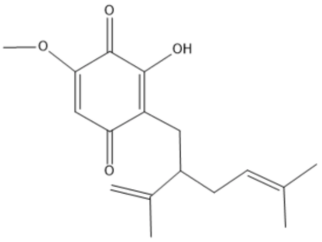

KJ001

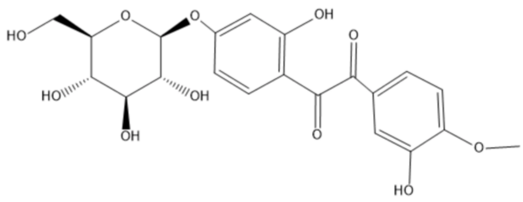

KE003

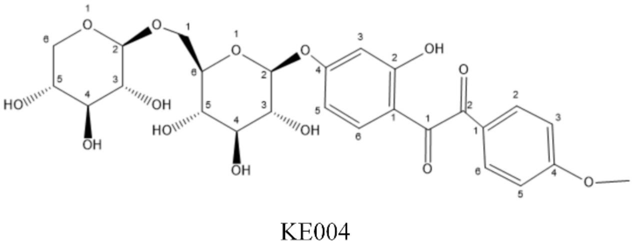

KE004

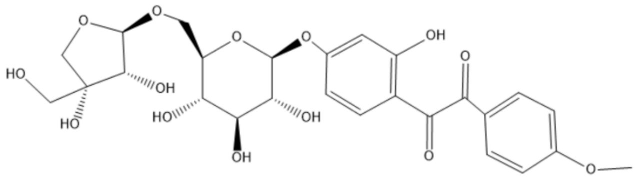

KE005

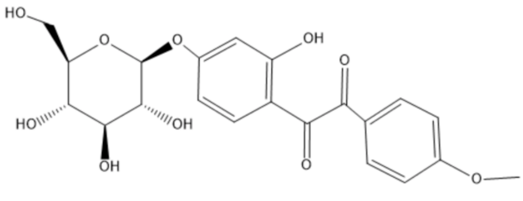

KE006

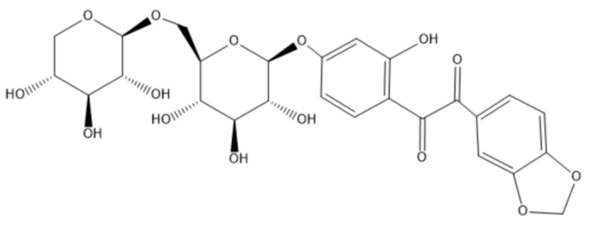

KE007

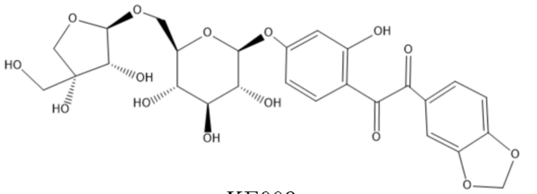

KE008

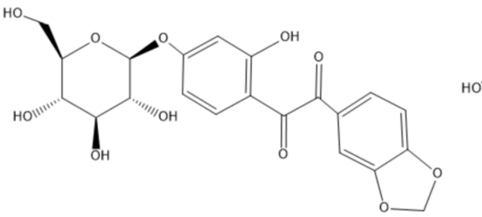

KE009

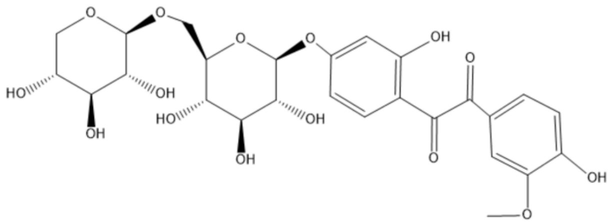

KE010

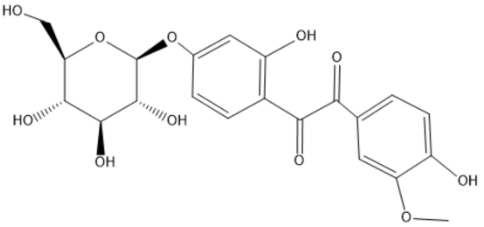

KE011

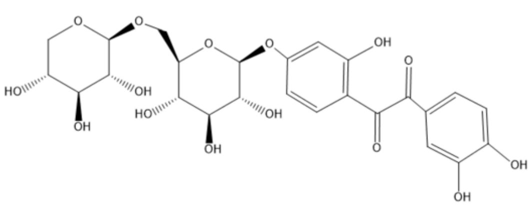

KE012

Supplementary Figure S4. Venn diagram summary of chemical compounds identified from Danggui Beimu Kushen Wan.

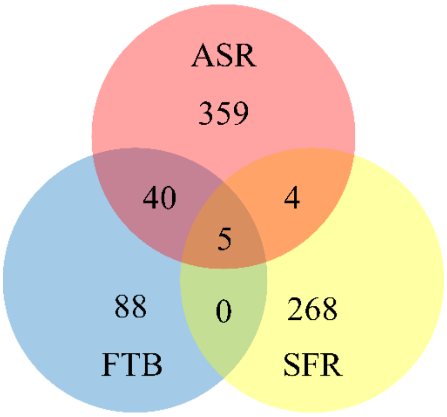

Note: ASR: Angelicae Sinensis Radix; FTB: Fritillariae Thunbergii Bulbus; SFR: Sophorae Flavescentis Radix.

Supplementary Figure S5. Flow chart of selection process for chemical compounds from Danggui Beimu Kushen Wan for molecular docking.

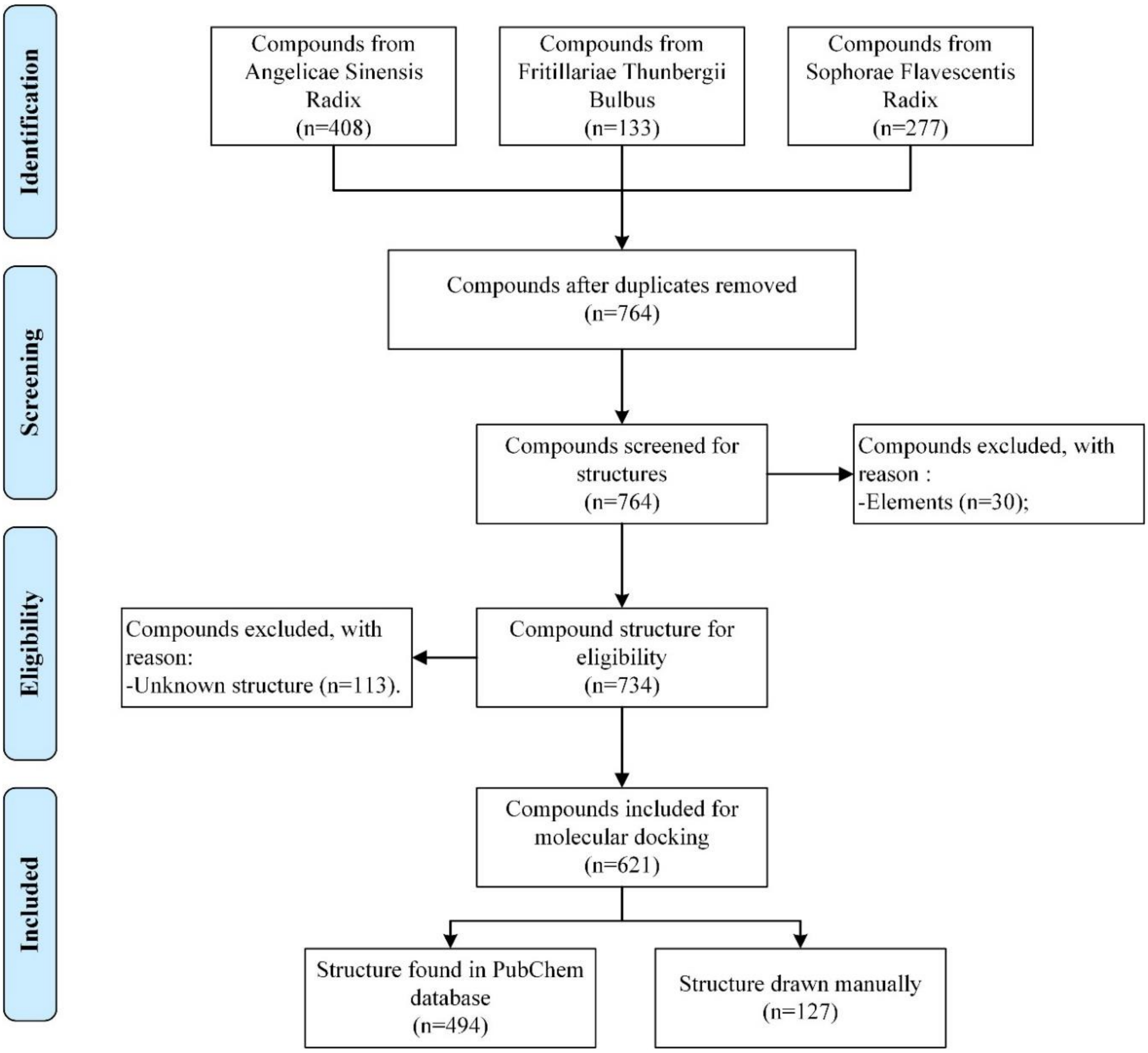

Supplementary Figure S6. Ligand–target interactions for compounds forming hydrogen bonds with one catalytic triad residue.

DA012

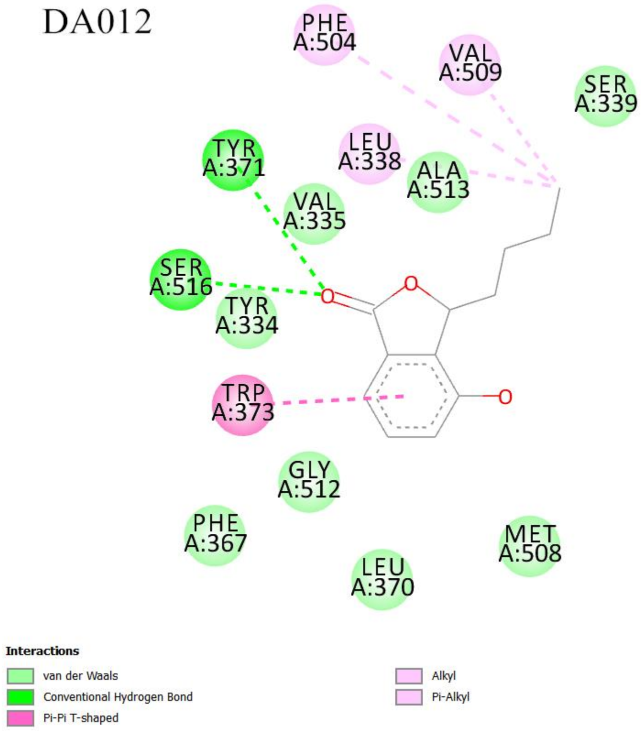

DA053

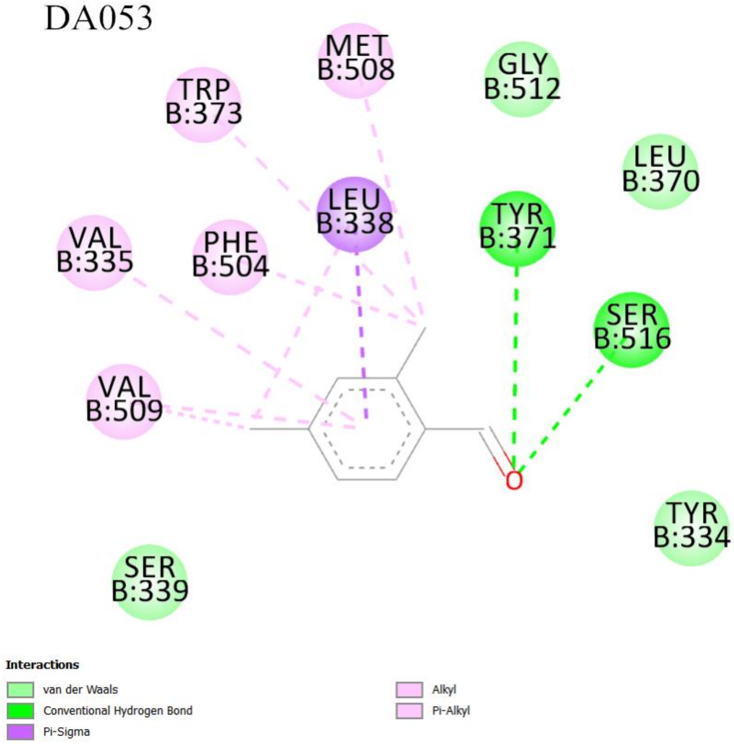

Note: DA012: 4-Hydroxy-3-butylphthalide; DA053: 2,4-Dimethylbenzaldehyde.

DA134

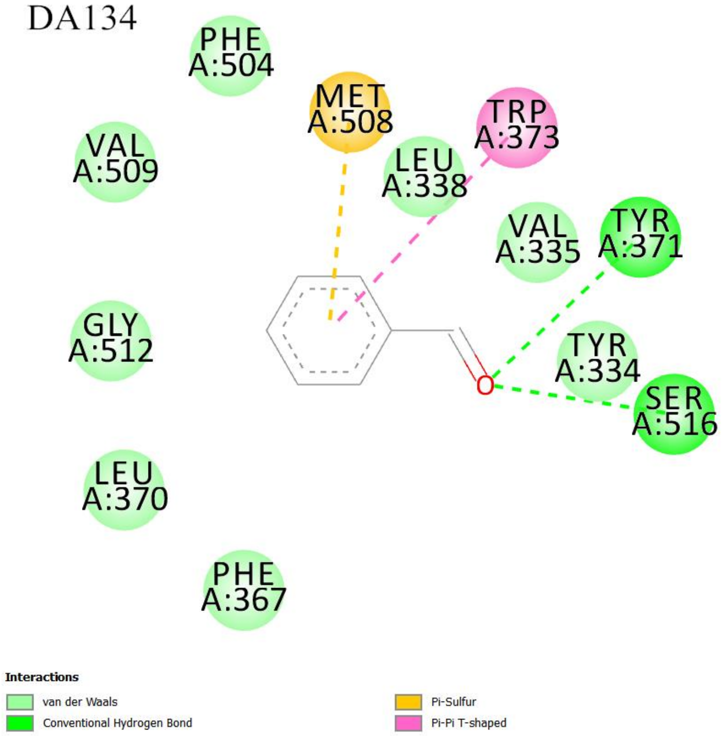

DA216

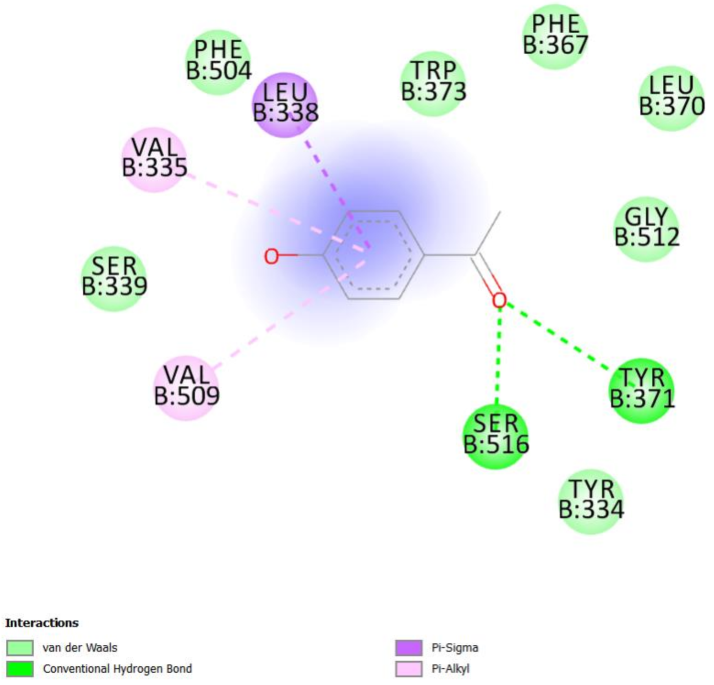

Note: DA134: Benzaldehyde; DA216: P-hydroxyacetophenone.

DB004

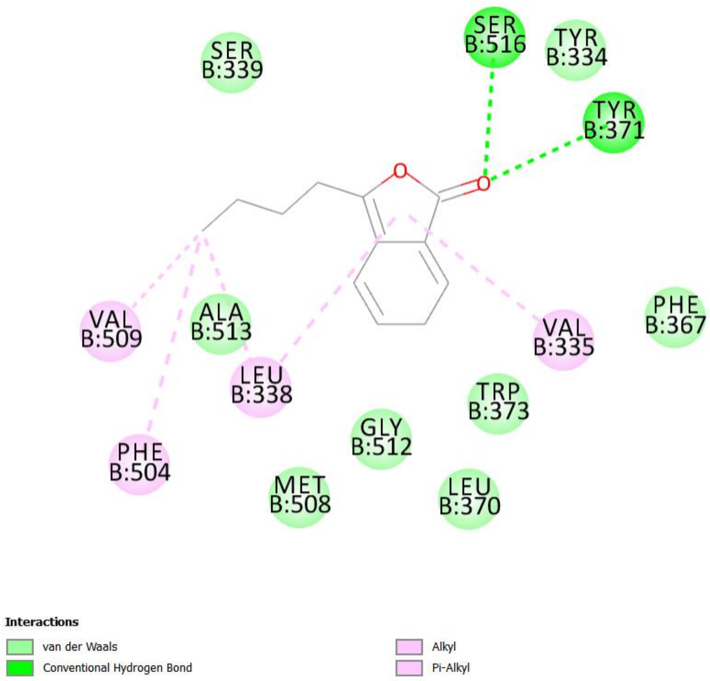

DB005

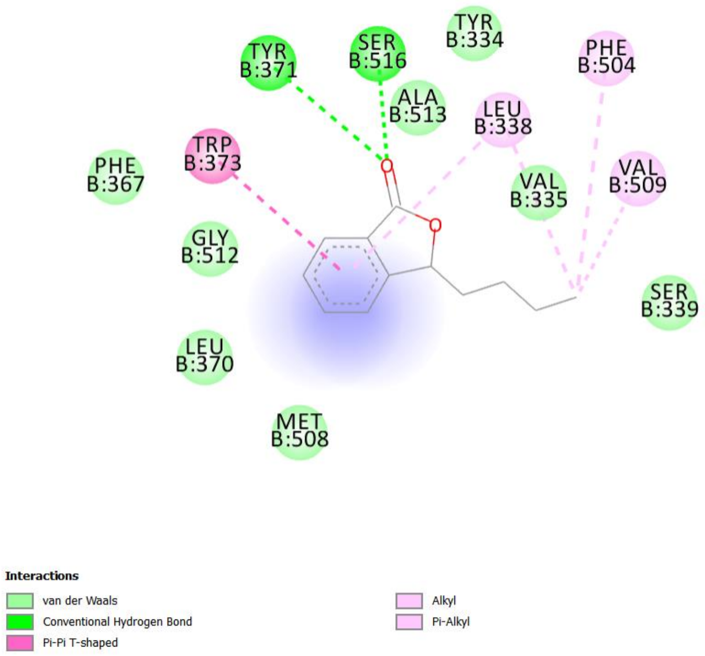

Note: DB004: E-butylidenephthalide; DB005: Butylphthalide.

DB024

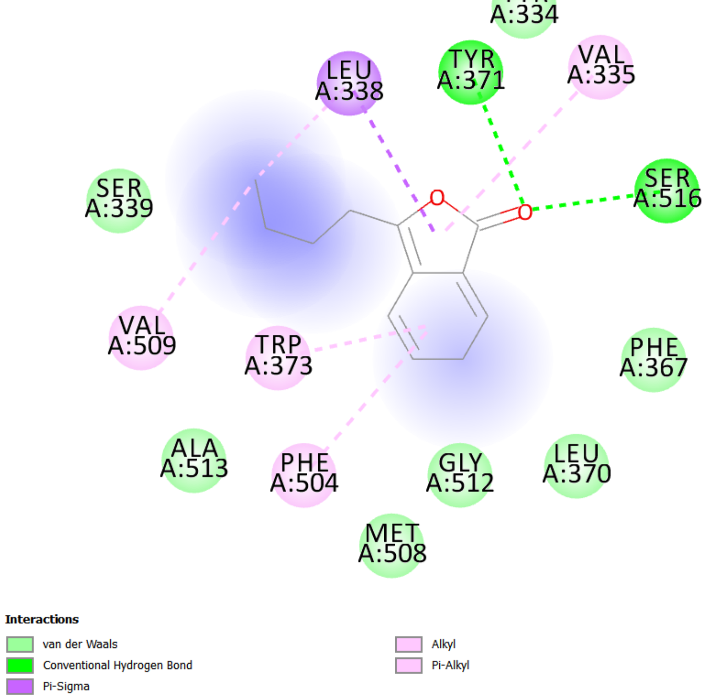

DA145

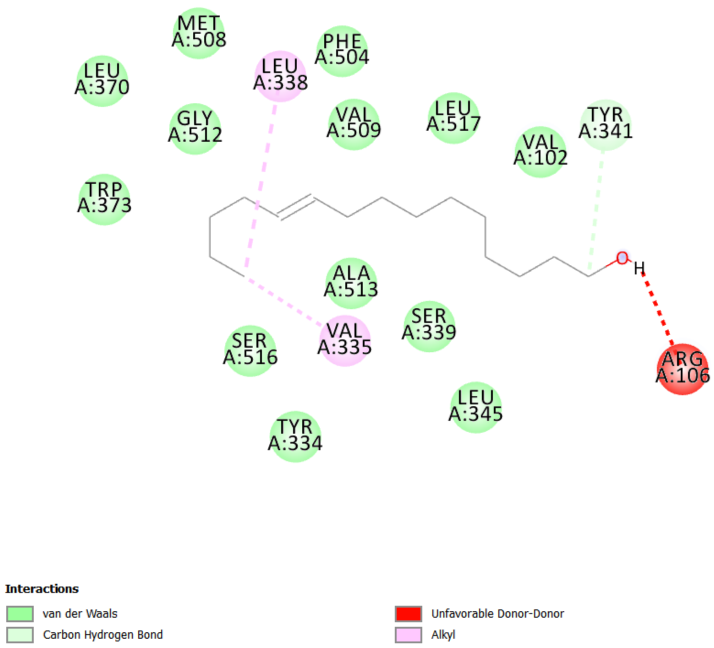

Note: DA145: E-10-pentadecenol; DB024: 3-Butylidenephthalide.

DA153

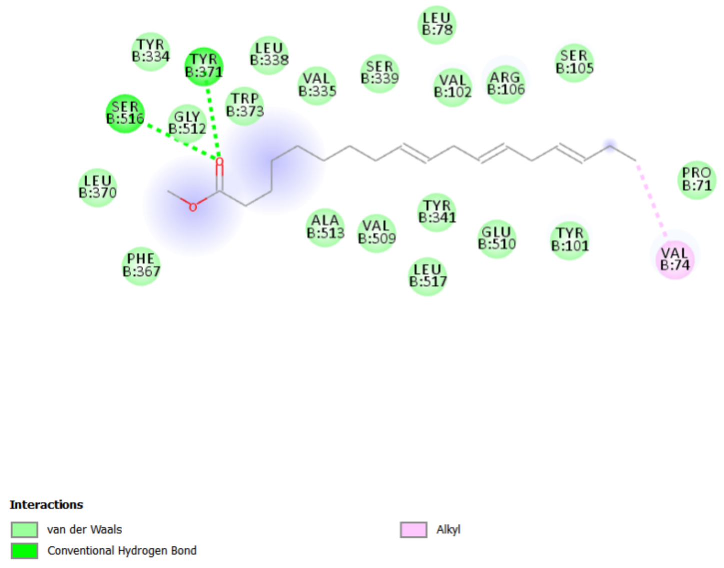

DA165

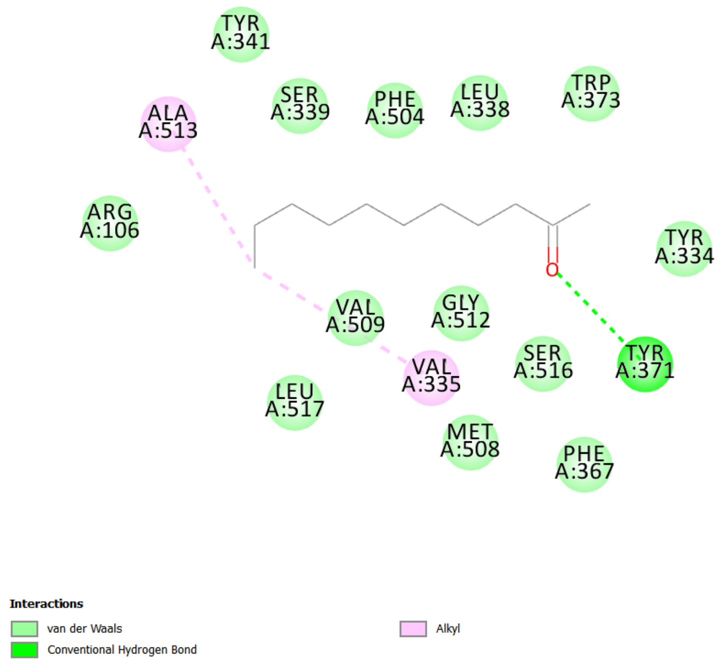

Note: DA153: Methyl linolenate; DA165: 2-Undecanone.

DA172

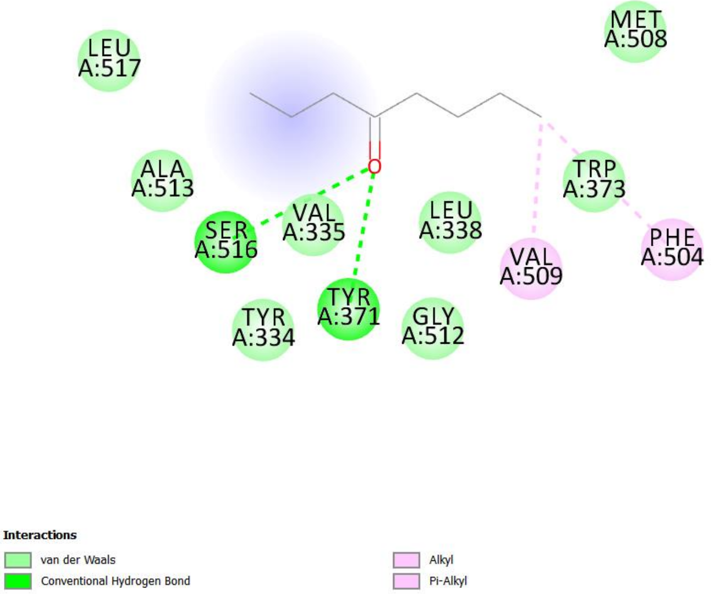

DA173

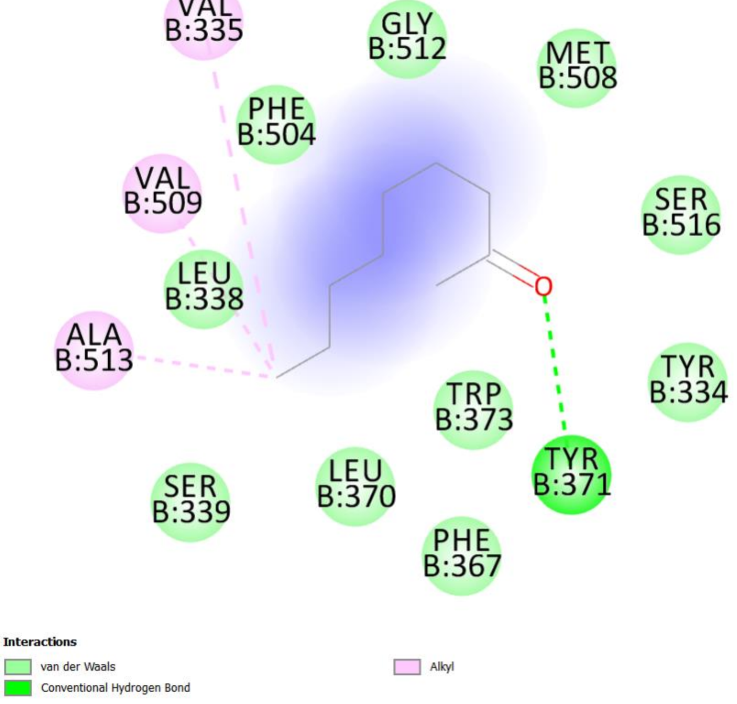

Note: DA172: 4-Octanone; DA173: 2-Nonanone.

DA196

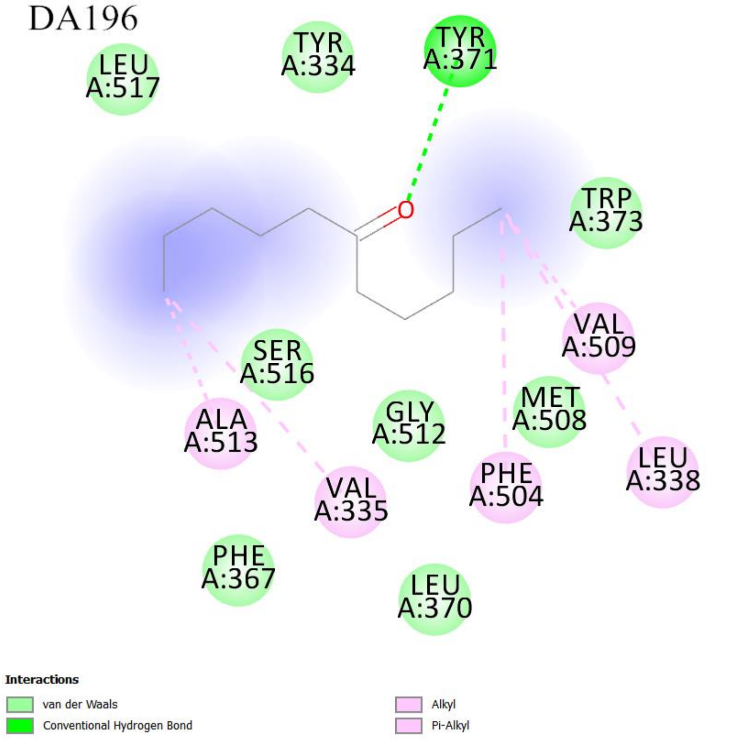

ZF02

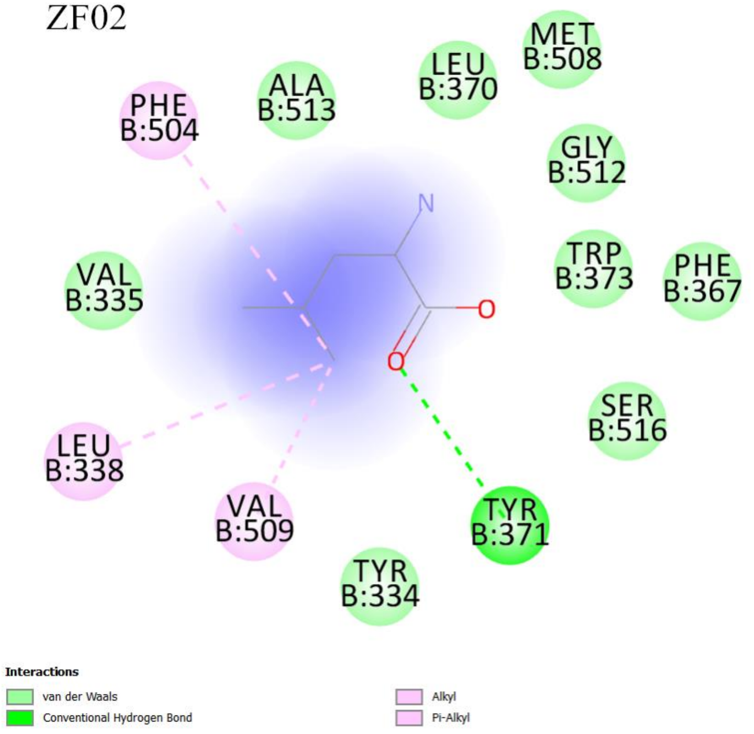

Note: DA196: 6-Undecanone; ZF02: Leucine.

Supplementary Tables

Supplementary Table S1. Summary of herbal compounds identified from Danggui Beimu KushenWan's ingredients.

No.	Derivatives and Constituents	Molecular Formula	PubChem CID/SID	Molecule Weight (g/mol)	References
**1. Angelicae Sinensis Radix**					
**DA**	**Compounds from essential oils (n=216)**				
DA001	Coniferyl ferulate	$C_{20}H_{20}O_6$	6441913	356.4	(Li et al., 2017; Ma et al., 2017; Y. Wang et al., 2016; Wei et al., 2016)
DA002	(E)-Coniferin	$C_{16}H_{22}O_8$	3496897	342.34	(Wei et al., 2016)
DA003	Guaiacylglycerol	$C_{10}H_{14}O_5$	14579	214.21	(Wei et al., 2016)
DA004	Butanal	C_4H_8O	261	72.11	(Li et al., 2016; Pei et al., 2017; Wei et al., 2016)
DA005	3,5-dimethylbenzaldehyde	$C_9H_{10}O$	34225	134.17	(X. Wang et al., 2018; Wei et al., 2016)
DA006	Camphene	$C_{10}H_{16}$	6616	136.23	(Li et al., 2016; Wei et al., 2016)
DA007	α-cedrene	$C_{15}H_{24}$	6431015	204.35	(Wei et al., 2016)
DA008	α-terpinolene	$C_{10}H_{16}$	11463	136.23	(Wei et al., 2016)
DA009	Octadecane	$C_{18}H_{38}$	11635	254.5	(Wei et al., 2016)
DA010	Guaiacol	$C_7H_8O_2$	460	124.14	(Wei et al., 2016)
DA011	Isobutanal	C_4H_8O	6561	72.11	(Wei et al., 2016)
DA012	4-Hydroxy-3-butylphthalide	$C_{12}H_{14}O_3$	275561394	206.24	(Wei et al., 2016)
DA013	M-ethylphenol	$C_8H_{10}O$	12101	122.16	(Wei et al., 2016)
DA014	2-Methylbutanal	$C_5H_{10}O$	7284	86.13	(Wei et al., 2016)
DA015	3-Methylbutanal	$C_5H_{10}O$	11552	86.13	(Wei et al., 2016)
DA016	O-cresol	C_7H_8O	335	108.14	(Wei et al., 2016)
DA017	β-Ocimene	$C_{10}H_{16}$	5281553	136.23	(Li et al., 2016; Liu et al., 2016; X. Wang et al., 2018; Wei et al., 2016)

No.	Derivatives and Constituents	Molecular Formula	PubChem CID/SID	Molecule Weight (g/mol)	References
DA018	Tridecane	$C_{13}H_{28}$	12388	184.36	(X. Wang et al., 2018; Wei et al., 2016)
DA019	Decanal	$C_{10}H_{20}O$	8175	156.26	(Li et al., 2016; Pei et al., 2017; Wei et al., 2016)
DA020	1,3,5-Undecatriene	$C_{11}H_{18}$	5367412	150.26	(Wei et al., 2016)
DA021	3,3'Z-6.7',7.6'-Diligustilide	$C_{24}H_{28}O_4$	NR #	380.48	(Wei et al., 2016)
DA022	1-Acetyl-β-carboline	$C_{13}H_{10}N_2O$	638667	210.23	(Wei et al., 2016)
DA023	Bergamiol	$C_{12}H_{20}O_2$	8294	196.29	(Wei et al., 2016)
DA024	5-Methylfurfural	$C_6H_6O_2$	12097	110.11	(Wei et al., 2016)
DA025	D-Limonene	$C_{10}H_{16}$	440917	136.23	(Pei et al., 2017; Wei et al., 2016)
DA026	α-Phellandrene	$C_{10}H_{16}$	7460	136.23	(Wei et al., 2016)
DA027	Spinasterol	$C_{29}H_{48}O$	5281331	412.7	(Wei et al., 2016; Wu et al., 2016; Xu, 2016; Zhu, 2018)
DA028	Magnolol	$C_{18}H_{18}O_2$	72300	266.3	(Wei et al., 2016; Xu, 2016)
DA029	Baicalin	$C_{21}H_{18}O_{11}$	64982	446.4	(Wei et al., 2016; Wu et al., 2016; Xu, 2016)
DA030	Octanal	$C_8H_{16}O$	454	128.21	(Li et al., 2016; Pei et al., 2017; X. Wang et al., 2018; Wei et al., 2016)
DA031	δ-Guaiene	$C_{15}H_{24}$	94275	204.35	(Li et al., 2016; X. Wang et al., 2018; Wei et al., 2016; Wu et al., 2016; Xu, 2016)
DA032	6-Undecanol	$C_{11}H_{24}O$	32045	172.31	(X. Wang et al., 2018; Wei et al., 2016)
DA033	Aromadendrene	$C_{15}H_{24}$	91354	204.35	(Li et al., 2016; Wei et al., 2016)
DA034	1,10-Didenhydroaristolane	$C_{15}H_{24}$	NR	204.35	(Wei et al., 2016)
DA035	Spathulenol	$C_{15}H_{24}O$	92231	220.35	(Li et al., 2016; X. Wang et al., 2018; Wei et al., 2016; M. Zhao et al., 2018)

No.	Derivatives and Constituents	Molecular Formula	PubChem CID/SID	Molecule Weight (g/mol)	References
DA036	Caryophyllene	$C_{15}H_{24}$	5281515	204.35	(Wei et al., 2016)
DA037	β-Eudesmol	$C_{15}H_{26}O$	91457	222.37	(Wei et al., 2016)
DA038	γ-Muurolene	$C_{15}H_{24}$	12313020	204.35	(Wei et al., 2016)
DA039	α-Pinene	$C_{10}H_{16}$	6654	136.23	(Li et al., 2016; Liu et al., 2016; Pei et al., 2017; Wei et al., 2016)
DA040	Z, Z'-3.3’α,7α.7’α-Diligustilide	$C_{24}H_{28}O_4$	NR #	380.48	(Wei et al., 2016)
DA041	Myrcene	$C_{10}H_{16}$	31253	136.23	(Li et al., 2016; Pei et al., 2017; Wei et al., 2016)
DA042	3, 9-Dihydroxyligustilide	$C_{12}H_{16}O_4$	NR #	224.26	(Wei et al., 2016)
DA043	10-Angeloylbutylphthalide	$C_{17}H_{20}O_4$	11572826	288.34	(Wei et al., 2016)
DA044	Borneol	$C_{10}H_{18}O$	1201518	154.25	(Wei et al., 2016)
DA045	Menthene-3-ol	$C_{10}H_{18}O$	NR	154.25	(Wei et al., 2016)
DA046	2-Methoxy-4-vinylphenol	$C_9H_{10}O_2$	332	150.17	(Li et al., 2016; Pei et al., 2017; X. Wang et al., 2018; Wei et al., 2016)
DA047	Copaene	$C_{15}H_{24}$	19725	204.35	(Wei et al., 2016)
DA048	Octahydro-3,8,8-trimethyl-6-methylene-1H,3A,7-methanoazulene	NR	NR	NR	(Wei et al., 2016)
DA049	2-santalene	NR	NR	NR	(Wei et al., 2016)
DA050	β-chamigrene	$C_{15}H_{24}$	442353	204.35	(Li et al., 2016; X. Wang et al., 2018; Wei et al., 2016; M. Zhao et al., 2018)
DA051	1,5,5-Trimethyl-6-methylene-cyclohexene	$C_{10}H_{16}$	578237	136.23	(Pei et al., 2017; X. Wang et al., 2018)
DA052	2,4,5-trimethylbenzaldehyde	$C_{10}H_{12}O$	22013	148.2	(Li et al., 2016; Pei et al., 2017; X. Wang et al., 2018)
DA053	2,4-dimethylbenzaldehyde	$C_{10}H_8$	61814	134.17	(Li et al., 2016; Pei et al., 2017)
DA054	(-)-alloaromadendrene	$C_{15}H_{24}$	10899740	204.35	(Li et al., 2016; X. Wang et al., 2018)

No.	Derivatives and Constituents	Molecular Formula	PubChem CID/SID	Molecule Weight (g/mol)	References
DA055	(-)-Thujopsene	$C_{15}H_{24}$	442402	204.35	(Li et al., 2016; X. Wang et al., 2018; M. Zhao et al., 2018)
DA056	α-Chamigrene	$C_{15}H_{24}$	442351	204.35	(Li et al., 2016)
DA057	Cuparene	$C_{15}H_{22}$	86895	202.33	(Li et al., 2016; X. Wang et al., 2018; M. Zhao et al., 2018)
DA058	β-Farnesene	$C_{15}H_{24}$	5281517	204.35	(Li et al., 2016; Pei et al., 2017; X. Wang et al., 2018)
DA059	Allo-ocimene	$C_{10}H_{16}$	5368821	136.23	(Li et al., 2016; Pei et al., 2017; X. Wang et al., 2018)
DA060	Bicyclogermacrene	$C_{15}H_{24}$	13894537	204.35	(X. Wang et al., 2018)
DA061	Cis-α-bisabolene	$C_{15}H_{24}$	5352653	204.35	(X. Wang et al., 2018)
DA062	β-Bisabolene	$C_{15}H_{24}$	10104370	204.35	(Li et al., 2016; X. Wang et al., 2018)
DA063	4-Allyloxyimino-2-carene	$C_{13}H_{19}NO$	9603708	205.3	(Liu et al., 2016)
DA064	β-Cedrene	$C_{15}H_{24}$	11106485	204.35	(Li et al., 2016; Pei et al., 2017; X. Wang et al., 2018)
DA065	6-Butyl-1,4-cycloheptadiene	$C_{11}H_{18}$	556470	136.23	(Li et al., 2016; Pei et al., 2017)
DA066	1,3,5,5-Tetramethyl-1,3-cyclohexadiene	$C_{10}H_{16}$	78453	136.23	(Liu et al., 2016)
DA067	1,5,5,6-Tetramethyl-1,3-cyclohexadiene	$C_{10}H_{16}$	10581	136.23	(X. Wang et al., 2018; M. Zhao et al., 2018)
DA068	1,3-diisopropenyl-6-methyl-cyclohexene	$C_{13}H_{20}$	577052	176.3	(Pei et al., 2017)
DA069	4-Methyl-1-(prop-1-en-2-yl) cyclohexene	NR	NR	NR	(Liu et al., 2016)
DA070	4-Methyl-3-(1-methylethylidene)-cyclohexene	$C_{10}H_{16}$	564722	136.23	(Pei et al., 2017)
DA071	1,2-Cyclooctadiene	C_8H_{12}	572661	108.18	(Liu et al., 2016)
DA072	1-Decene	$C_{10}H_{20}$	13381	140.27	(Pei et al., 2017; X. Wang et al., 2018)
DA073	5-Methyl-2-decene	$C_{11}H_{22}$	22171977	154.29	(Pei et al., 2017)
DA074	γ-Elemene	$C_{15}H_{24}$	6432312	204.35	(Li et al., 2016; X. Wang et al., 2018)
DA075	Elixene	$C_{15}H_{24}$	275390917	204.35	(Li et al., 2016)
DA076	1-Hexadecene	$C_{16}H_{32}$	12395	224.42	(Pei et al., 2017)

No.	Derivatives and Constituents	Molecular Formula	PubChem CID/SID	Molecule Weight (g/mol)	References
DA077	5-Methyl-3-(1-methylethylidene)-1, 4-hexadiene	$C_{10}H_{16}$	578220	136.23	(Pei et al., 2017)
DA078	β-Himachalene	$C_{15}H_{24}$	15095	204.35	(Li et al., 2016; Pei et al., 2017; X. Wang et al., 2018; M. Zhao et al., 2018)
DA079	α-Himachalene	$C_{15}H_{24}$	11830551	204.35	(Li et al., 2016)
DA080	Ledene	$C_{15}H_{24}$	10910653	204.35	(Li et al., 2016; Pei et al., 2017)
DA081	Leucine	$C_6H_{13}NO_2$	6106	131.17	(Li et al., 2017; S. Zhu et al., 2017)
DA082	Limonene	$C_{10}H_{16}$	22311	136.23	(Li et al., 2016; X. Wang et al., 2018)
DA083	D-longifolene	$C_{15}H_{24}$	1796220	204.35	(M. Zhao et al., 2018)
DA084	Isolongifolene	$C_{15}H_{24}$	11127402	204.35	(Li et al., 2016)
DA085	Longipinene	$C_{15}H_{24}$	16217635	204.35	(Li et al., 2016; Pei et al., 2017; M. Zhao et al., 2018)
DA086	2,6,6-trimethylbicyclo-[3.1.1]hept-2-ene	$C_{10}H_{16}$	NR	136.23	(Pei et al., 2017)
DA087	5-Ethylidene-2-norbornene	C_9H_{12}	27756	120.19	(Liu et al., 2016)
DA088	3,7-Dimethyl-1,3,7-octatriene	$C_{10}H_{16}$	5320249	136.23	(Pei et al., 2017)
DA089	β-Pinene	$C_{10}H_{16}$	14896	136.23	(Li et al., 2016; X. Wang et al., 2018)
DA090	Sabinene	$C_{10}H_{16}$	18818	136.23	(Li et al., 2016)
DA091	γ-Terpinene	$C_{10}H_{16}$	7461	136.23	(Li et al., 2016; X. Wang et al., 2018)
DA092	1-Pentadecene	$C_{15}H_{30}$	25913	210.4	(X. Wang et al., 2018)
DA093	1-(Methylpropyl)-4-(1',1',2'-trichloro-3'-ethylallyl) benzene	$C_{15}H_{22}Cl_3$	5373097	305.7	(X. Wang et al., 2018)
DA094	1-methoxy-4-[1-propenyl]-benzene	$C_{10}H_{12}O$	NR	148.21	(Pei et al., 2017)
DA095	Ethylbenzene	C_8H_{10}	7500	106.16	(Li et al., 2016)
DA096	Methylbenzene	C_7H_8	1140	92.14	(Li et al., 2016)
DA097	Pentylbenzene	$C_{11}H_{16}$	10864	148.24	(Li et al., 2016; Pei et al., 2017)
DA098	P-xylene	C_8H_{10}	7809	106.16	(Li et al., 2016)
DA099	Squalene	$C_{30}H_{50}$	638072	410.7	(X. Wang et al., 2018)

No.	Derivatives and Constituents	Molecular Formula	PubChem CID/SID	Molecule Weight (g/mol)	References
DA100	Styrene	C_8H_8	7501	104.15	(Li et al., 2016)
DA101	Hexanoic acid, 2-butenyl ester	$C_{10}H_{18}O_2$	5369196	170.25	(Pei et al., 2017)
DA102	Ethyl butyrate	$C_6H_{12}O_2$	7762	116.16	(Liu et al., 2016)
DA103	Hexadecanoic acid, trimethylsilyl ester	$C_{19}H_{40}O_2Si$	521638	328.6	(X. Wang et al., 2018)
DA104	Hexadecanoic acid, butyl ester	$C_{20}H_{40}O_2$	8090	312.5	(X. Wang et al., 2018)
DA105	Hydratropic acid	$C_9H_{10}O_2$	10296	150.17	(Liu et al., 2016)
DA106	Stearic acid	$C_{18}H_{36}O_2$	5281	284.5	(L. Wang et al., 2016; X. Wang et al., 2018)
DA107	Stearolic acid	$C_{18}H_{32}O_2$	68167	280.4	(Pei et al., 2017)
DA108	Tetradecanoic acid	$C_{14}H_{28}O_2$	11005	228.37	(Pei et al., 2017)
DA109	Trans-2-hexen-1-yl ester, phenylacetic acid	$C_{14}H_{18}O_2$	5368236	218.29	(Pei et al., 2017)
DA110	(-)-4-terpineol	$C_{10}H_{18}O$	5325830	154.25	(Pei et al., 2017)
DA111	(-)-α-Terpineol	$C_{10}H_{18}O$	443162	154.25	(Pei et al., 2017; X. Wang et al., 2018)
DA112	(6-hydroxymethyl-2,3-dimethylphenyl)methanol	$C_{10}H_{14}O_2$	590603	166.22	(Pei et al., 2017)
DA113	[Z]-2-[3-cyclopropyl-7-norcaranyl]acetate	$C_{13}H_{20}O_2$	NR	208.3	(Pei et al., 2017)
DA114	10-Undecenal	$C_{11}H_{20}O$	8187	168.28	(Pei et al., 2017)
DA115	1H-Benzimidazol-2-amine	$C_7H_7N_3$	13624	133.15	(Liu et al., 2016)
DA116	1-Tridecyn-4-ol	$C_{13}H_{24}O$	557899	196.33	(Pei et al., 2017)
DA117	2,5-dimethyl-3-hexanol acetate	$C_{10}H_{20}O_2$	537590	172.26	(Pei et al., 2017)
DA118	2,9-Dimethyldecane	$C_{12}H_{26}$	517733	170.34	(Pei et al., 2017)
DA119	2-Methoxy-(4'-hydroxyphenyl)ethanol	$C_9H_{12}O_3$	NR #	168.19	(Wei, 2016)
DA120	2-Methyldodecane	$C_{13}H_{28}$	15270	184.36	(Pei et al., 2017)
DA121	Dodecane	$C_{12}H_{26}$	8182	170.33	(X. Wang et al., 2018)
DA122	2-Methylnonane	$C_{10}H_{22}$	13379	142.28	(Pei et al., 2017)
DA123	2-Propylphenol	$C_9H_{12}O$	12570	125.70	(Zhu, 2018)
DA124	2-Octanal	$C_{10}H_{16}$	NR	136.24	(Pei et al., 2017)
DA125	3,4-Dimethylbenzaldehyde	$C_9H_{10}O$	22278	134.17	(Pei et al., 2017)
DA126	4-Methyl-2-tert-octylphenol	$C_{15}H_{24}O$	521263	220.35	(Pei et al., 2017)
DA127	4-Methyl-5-decanol	$C_{11}H_{24}O$	557858	172.31	(Pei et al., 2017)

No.	Derivatives and Constituents	Molecular Formula	PubChem CID/SID	Molecule Weight (g/mol)	References
DA128	5,6-dihydro-4-[2,3-dimethyl-2-buten-4-yl]-2H-pyran-2-one	$C_{11}H_{16}O_2$	535261	180.24	(Pei et al., 2017)
DA129	5,9,9-Trimethyl-spiro[3.5]non-5-en-1-one	$C_{12}H_{18}O$	578422	178.27	(Pei et al., 2017)
DA130	5-Decen-1-ol	$C_{10}H_{20}O$	5283292	156.26	(Pei et al., 2017)
DA131	5-Formyl-2,4-dimethyl-pyrrole-3-carbonitrile	$C_8H_8N_2O$	NR	148.16	(Liu et al., 2016)
DA132	6,7-dimethylbicyclo[4.2.0]octane	$C_{10}H_{18}$	NR	138.25	(Pei et al., 2017)
DA133	Oleyl alcohol	$C_{18}H_{36}O$	5284499	268.5	(X. Wang et al., 2018)
DA134	Benzaldehyde	C_7H_6O	240	106.12	(Li et al., 2016)
DA135	Butylicene phthalide	$C_{12}H_{12}O_2$	NR	188.23	(Pei et al., 2017)
DA136	Butyl-linolenate	$C_{22}H_{38}O_2$	20833774	334.5	(X. Wang et al., 2018)
DA137	Calarene	$C_{15}H_{24}$	28481	204.35	(Li et al., 2016)
DA138	Campesterol	$C_{28}H_{48}O$	173183	400.7	(X. Wang et al., 2018)
DA139	Carvacrol	$C_{10}H_{14}O$	10364	150.22	(Pei et al., 2017)
DA140	Carveol	$C_{10}H_{16}O$	7438	152.23	(Li et al., 2016)
DA141	Cis-7-tetradecen-1-ol	$C_{14}H_{28}O$	5362795	212.37	(Pei et al., 2017)
DA142	Cis-Z,Z′-3α,7α′,7α,3α′-dihydroxyligustilide	NR	NR	NR	(Li et al., 2017)
DA143	Dodecanal	$C_{12}H_{24}O$	8194	184.32	(Pei et al., 2017; X. Wang et al., 2018)
DA144	Dodecyloxirane	$C_{14}H_{28}O$	18604	212.37	(Pei et al., 2017)
DA145	E-10-pentadecenol	$C_{15}H_{30}O$	5364466	226.4	(Pei et al., 2017)
DA146	E-7-tetradecen-1-ol	$C_{14}H_{28}O$	5362726	212.37	(Pei et al., 2017)
DA147	Eicosane	$C_{20}H_{42}$	8222	282.5	(X. Wang et al., 2018)
DA148	Espatulenol	$C_{15}H_{24}O$	6432640	220.35	(X. Wang et al., 2018)
DA149	Heptane	C_7H_{16}	8900	100.2	(Pei et al., 2017)
DA150	Hexadecene	$C_{16}H_{32}$	NR	224.42	(X. Wang et al., 2018)
DA151	Iso-butylicene phthalide	$C_{12}H_{12}O_2$	NR	188.23	(Pei et al., 2017)
DA152	Methyl linoleate	$C_{19}H_{34}O_2$	5284421	294.5	(X. Wang et al., 2018; Zhu, 2018)
DA153	Methyl linolenate	$C_{19}H_{32}O_2$	5319706	292.5	(X. Wang et al., 2018)
DA154	Methyl palmitate	$C_{17}H_{34}O_2$	8181	270.5	(X. Wang et al., 2018)
DA155	N-Hexyl alcohol	$C_6H_{14}O$	8103	102.17	(Li et al., 2016)

No.	Derivatives and Constituents	Molecular Formula	PubChem CID/SID	Molecule Weight (g/mol)	References
DA156	Nonanal	$C_9H_{18}O$	31289	142.24	(Li et al., 2016)
DA157	Nonane	C_9H_{20}	8141	128.25	(Pei et al., 2017)
DA158	Octane	C_8H_{18}	356	114.23	(Pei et al., 2017)
DA159	Patchouli alcohol	$C_{15}H_{26}O$	10955174	222.37	(Pei et al., 2017)
DA160	Palustrol	$C_{15}H_{26}O$	110745	222.37	(X. Wang et al., 2018)
DA161	1-Pentadecanol	$C_{15}H_{32}O$	12397	228.41	(X. Wang et al., 2018)
DA162	Stigmasterol	$C_{29}H_{48}O$	5280794	412.7	(X. Wang et al., 2018)
DA163	N-Tetradecane	$C_{14}H_{30}$	12389	198.39	(X. Wang et al., 2018)
DA164	Trans,trans-2,4-Hexadienyl acetate	$C_8H_{12}O_2$	5363491	140.18	(Pei et al., 2017)
DA165	2-Undecanone	$C_{11}H_{22}O$	8163	170.29	(X. Wang et al., 2018)
DA166	β-Elemenone	$C_{15}H_{22}O$	10955018	218.33	(Liu et al., 2016)
DA167	2-(1-oxypentyl)-benzoic acid methyl ester	$C_{13}H_{16}O_3$	NR	220.27	(L. Wang et al., 2016)
DA168	1,2-Xylene	C_8H_{10}	7237	106.16	(Li et al., 2016)
DA169	P-cymene	$C_{10}H_{14}$	7463	134.22	(Li et al., 2016)
DA170	2,2'-Methylenebis(4-methyl-6-tert-butylphenol)	$C_{23}H_{32}O_2$	8398	340.5	(X. Wang et al., 2018)
DA171	Tropone	C_7H_6O	10881	106.12	(X. Wang et al., 2018)
DA172	4-Octanone	$C_8H_{16}O$	11516	128.21	(Li et al., 2016)
DA173	2-Nonanone	C_9H_8O	13187	142.24	(Li et al., 2016)
DA174	1,13-Tetradecadiene	$C_{14}H_{26}$	30875	194.36	(X. Wang et al., 2018)
DA175	5-Acetoxymethylfurfural	$C_8H_8O_4$	66349	168.15	(Wu et al., 2016; Xu, 2016)
DA176	2,6,6-Trimethylbicyclo[3,1,1]hept-2-ene	$C_{16}H_{22}O$	NR #	230.34	(M. Zhao et al., 2018)
DA177	5-Hydroxymethylfurfural	$C_6H_6O_3$	237332	126.11	(Wu et al., 2016; Xu, 2016)
DA178	Nonylcyclopropane	$C_{12}H_{24}$	522556	168.32	(X. Wang et al., 2018)
DA179	10,13-Octadecadienoic acid methyl ester	$C_{19}H_{34}O_2$	549045	294.5	(X. Wang et al., 2018)
DA180	(E,Z)-Alloocimene	$C_{10}H_{16}$	5462627	136.23	(X. Wang et al., 2018)
DA181	(Z)-4-propenyl-2-methoxyphenol	$C_{10}H_{12}O_2$	NR	164.2	(X. Wang et al., 2018)
DA182	Trans-2-nonenal	$C_9H_{16}O$	5283335	140.22	(Li et al., 2016)
DA183	2-Methoxy-2-(4'-Hydroxyphenyl)ethanol	$C_9H_{12}O_3$	22297411	168.19	(Wu et al., 2016)
DA184	Cyclohexene, 3-(1,5-dimethyl-4-hexenyl)-6-methylene-	$C_{15}H_{24}$	519764	204.35	(X. Wang et al., 2018; M. Zhao et al., 2018)
DA185	Cyclopropane, trimethyl(2-methyl-1-propenylidene)-	$C_{10}H_{16}$	139833	136.23	(X. Wang et al., 2018)

No.	Derivatives and Constituents	Molecular Formula	PubChem CID/SID	Molecule Weight (g/mol)	References
DA186	Cyclohexane, 1-methylene-4-(1-methylethenyl)-	$C_{10}H_{16}$	68140	136.23	(X. Wang et al., 2018)
DA187	1,3,6-Octatriene, 3,7-dimethyl-, (E)-	$C_{10}H_{16}$	NR	136.23	(M. Zhao et al., 2018)
DA188	6,6-dimethyl-2-methylene-, (1S)-Bicyclo(3.1.1)heptane	$C_{10}H_{16}$	24848167	136.23	(M. Zhao et al., 2018)
DA189	3'-methoxybenzonitrile-(1',2'-b)-1,4-triethylenediamine	$C_{11}H_{14}N_2O$	NR	190.25	(M. Zhao et al., 2018)
DA190	1,5,6,7-Tetramethylbicyclo[3.2.0]hepta-2,6-diene	$C_{11}H_{16}$	583876	148.24	(M. Zhao et al., 2018)
DA191	2(3H)-Benzofuranone, 4,5,6,7-tetramethyl-	$C_{12}H_{14}O_2$	595374	190.24	(M. Zhao et al., 2018)
DA192	Dihydrocoumarin, 5,7,8-trimethyl-	$C_{12}H_{14}O_2$	605390	190.24	(M. Zhao et al., 2018)
DA193	Cyclohexane, 1-ethenyl-1-methyl-2-(1-methylethenyl)-4-(1-methylethylidene)-	$C_{15}H_{24}$	94254	204.35	(M. Zhao et al., 2018)
DA194	Cyclohexane,1-ethylene-1-methyl-2-(1-methylethenyl)-4-(1R,2R)-(1-methylethylidene)-	$C_{15}H_{24}$	NR	204.35	(M. Zhao et al., 2018)
DA195	Cyclohexene, 3-(1,5-dimethyl-4-ethenyl)-6-methylene-[S-(R*,S*)]	$C_{15}H_{24}$	NR	204.35	(M. Zhao et al., 2018)
DA196	6-Undecanone	$C_{11}H_{22}O$	13561	170.29	(X. Wang et al., 2018)
DA197	2-Hydroxy-2-(4'-ethoxyethyl)ethanol	NR	NR	NR	(Wu et al., 2016)
DA198	Di(2-ethylhexyl)phthalate	$C_{24}H_{38}O_4$	8343	390.6	(X. Wang et al., 2018)
DA199	Ethyl linoleate	$C_{20}H_{36}O_2$	5282184	308.5	(X. Wang et al., 2018)
DA200	Propoxur	$C_{11}H_{15}NO_3$	4944	209.24	(X. Wang et al., 2018)
DA201	(3R,8S)-falcarindiol	$C_{17}H_{24}O_2$	5281148	260.399	(Li et al., 2017; Wu et al., 2016; Xu, 2016; Zhu, 2018)
DA202	β-Sitosterol	$C_{29}H_{50}O$	222284	414.7	(Ma et al., 2016; Wei, 2016; Zhu, 2018)
DA203	γ-Sitosterol	$C_{29}H_{50}O$	457801	414.7	(X. Wang et al., 2018)
DA204	1-(3',4'-dihaydroxycinnamoyl)-cyclopentane-2,3-diol	NR	NR	NR	(Luo et al., 2017)
DA205	4,10-Aromadendranediol	$C_{15}H_{26}O_2$	14312736	238.37	(Zhu, 2018)
DA206	Benzyl alcohol	C_7H_8O	244	108.14	(Li et al., 2016)
DA207	Daucosterol	$C_{35}H_{60}O_6$	5742590	576.8	(Ma et al., 2016; Wei, 2016; Zhu, 2018)
DA208	Globulol	$C_{15}H_{26}O$	101716	222.37	(X. Wang et al., 2018)
DA209	1-Pentanone, 1-phenyl-	$C_{11}H_{14}O$	66093	162.23	(Li et al., 2016)
DA210	Undecane	$C_{11}H_{24}$	14257	156.31	(X. Wang et al., 2018)

No.	Derivatives and Constituents	Molecular Formula	PubChem CID/SID	Molecule Weight (g/mol)	References
DA211	D-threo-guaiacylglycerol 8-O-β-glucopyranoside	$C_{16}H_{24}O_{10}$	NR #	376.36	(Wei, 2016)
DA212	Hexanal	$C_6H_{12}O$	6184	100.16	(Li et al., 2016)
DA213	Isophorone	$C_9H_{14}O$	6544	138.21	(Li et al., 2016)
DA214	Vanillin	$C_8H_8O_3$	1183	152.15	(Wu et al., 2016; Xu, 2016; Zhu, 2018)
DA215	P-cresol	C_7H_8O	2879	108.14	(Wu et al., 2016)
DA216	P-hydroxyacetophenone	$C_8H_8O_2$	7469	136.15	(Zhu, 2018)
**DB**	**Phthalides (n=39)**				
DB001	Z-ligustilide	$C_{12}H_{14}O_2$	529865	190.24	(Li et al., 2017; Liu et al., 2016; Liu, 2018; L. Wang et al., 2016; X. Wang et al., 2018; Wei et al., 2016; C. Wu et al., 2018)
DB002	E-ligustilide	$C_{12}H_{14}O_2$	5877292	190.24	(Liu et al., 2016; L. Wang et al., 2019; L. Wang et al., 2016; X. Wang et al., 2018; Wei et al., 2016)
DB003	Z-butylidenephthalide	$C_{12}H_{12}O_2$	5352899	188.22	(Gong et al., 2016; L. Wang et al., 2016; Wei et al., 2016; M. Zhao et al., 2018)
DB004	E-butylidenephthalide	$C_{12}H_{12}O_2$	275559960	188.22	(Gong et al., 2016; L. Wang et al., 2016; Wei et al., 2016)
DB005	Butylphthalide	$C_{12}H_{14}O_2$	61361	190.24	(L. Wang et al., 2016; Wei et al., 2016; Wu et al., 2016; Xu, 2016)
DB006	Senkyunolide A	$C_{12}H_{16}O_2$	3085257	192.25	(Ma et al., 2017; L. Wang et al., 2016; Wei et al., 2016)
DB007	Senkyunolide I	$C_{12}H_{16}O_4$	11521428	224.25	(Liu, 2018; Ma et al., 2017; Wei et al., 2016)
DB008	Z-ligustulide dimer E-232	$C_{24}H_{28}O_4$	NR #	380.48	(Wei et al., 2016)

No.	Derivatives and Constituents	Molecular Formula	PubChem CID/SID	Molecule Weight (g/mol)	References
DB009	Senkyunolide H	$C_{12}H_{16}O_4$	5321251	224.25	(Ma et al., 2017; Y. Wang et al., 2016; Wei et al., 2016; G. Wu et al., 2018)
DB010	Senkyunolide P	$C_{24}H_{30}O_4$	91731751	382.5	(Wei et al., 2016)
DB011	Levistolide A	$C_{24}H_{28}O_4$	70698035	380.5	(Y. Wang et al., 2016; Wei et al., 2016)
DB012	Riligustilide	$C_{24}H_{28}O_4$	6442656	380.5	(Gong et al., 2016; Wei et al., 2016)
DB013	Tokinolide B	$C_{24}H_{28}O_4$	11090206	380.5	(Y. Wang et al., 2016; Wei et al., 2016; Wu et al., 2016; Xu, 2016; Zhu, 2018)
DB014	Neocnidilide	$C_{12}H_{18}O_2$	3083857	194.27	(Wei et al., 2016)
DB015	Ansaspirolide	$C_{24}H_{26}O_4$	44575265	378.5	(Wei et al., 2016)
DB016	Z,Z’-6.8’,7.3’-diligustilide	$C_{24}H_{28}O_4$	NR #	380.48	(Wei et al., 2016)
DB017	Angelicide	$C_{24}H_{28}O_4$	5316848	380.48	(Y. Wang et al., 2016; Wei et al., 2016)
DB018	Z,Z’-3.3’8.8’-diligustilide	$C_{24}H_{28}O_4$	NR #	380.48	(Y. Wang et al., 2016; Wei et al., 2016)
DB019	Senkyunolide F	$C_{12}H_{14}O_3$	NR #	206.24	(Y. Wang et al., 2016; Wei et al., 2016)
DB020	Ligustilide dimer 1	$C_{24}H_{28}O_4$	NR	380.48	(X. Wang et al., 2018)
DB021	Ligustilide dimer 2	$C_{24}H_{28}O_4$	NR	380.48	(X. Wang et al., 2018)
DB022	Ligustilide dimer 3	$C_{24}H_{28}O_4$	NR	380.48	(X. Wang et al., 2018)
DB023	Ligustilide dimer 4	$C_{24}H_{28}O_4$	NR	380.48	(N. Wang et al., 2017; X. Wang et al., 2018)
DB024	3-Butylidenephthalide	$C_{12}H_{12}O_2$	642376	188.22	(Li et al., 2017; Li et al., 2016; Liu et al., 2016; Ma et al., 2016; N. Wang et al., 2017; X. Wang et al., 2018; G. Wu et al., 2018)
DB025	3-N-butylphthalide	$C_{12}H_{14}O_2$	61361	190.24	(Li et al., 2016; G. Wu et al., 2018)

No.	Derivatives and Constituents	Molecular Formula	PubChem CID/SID	Molecule Weight (g/mol)	References
DB026	3-Butylidene-7-hydroxyphthalide	$C_{12}H_{12}O_3$	5281559	204.22	(L. Wang et al., 2019)
DB027	Bergapten	$C_{12}H_8O_4$	2355	216.19	(Jiang et al., 2019)
DB028	Senkyunolide E	$C_{12}H_{12}O_3$	11830530	204.22	(Y. Wang et al., 2016)
DB029	Senkyunolide G	$C_{12}H_{16}O_3$	10013283	208.25	(L. Wang et al., 2019)
DB030	Senkyunolide R	$C_{12}H_{16}O_5$	101686923	240.25	(Ma et al., 2016; Wei, 2016)
DB031	Senkyunolide S	$C_{12}H_{16}O_5$	101686926	240.25	(Ma et al., 2016; Wei, 2016)
DB032	Z, Z′-6,6′,7,3α′-diligustilide	$C_{24}H_{28}O_4$	NR #	380.5	(Li et al., 2017; Zhu, 2018)
DB033	E-6,7-dihydroxydihydroligustilide	$C_{12}H_{16}O_4$	NR	224.26	(L. Wang et al., 2016)
DB034	Senkyunolide D	$C_{12}H_{14}O_4$	NR #	222.24	(L. Wang et al., 2016)
DB035	Z-6,7-epoxyligustilide	$C_{12}H_{14}O_3$	5317139	206.24	(Liu, 2018; L. Wang et al., 2016)
DB036	Cis-Z,Z’-3a. 7a’,7a.3a′-dihydroxyligustilide	$C_{24}H_{28}O_4$	NR #	380.48	(Gong et al., 2016)
DB037	Tokinolide A	$C_{12}H_{14}O_2$	NR #	190.24	(Gong et al., 2016)
DB038	Tokinolide C	$C_{24}H_{32}O_3$	NR #	368.52	(Gong et al., 2016)
DB039	11-Angeloylsenkyunolide F		NR		(Liu, 2018)
**DC**	**Organic acids (n=26)**				
DC001	Protocatechuic acid	$C_7H_6O_4$	72	154.12	(Wei et al., 2016)
DC002	Phthalic acid	$C_8H_6O_4$	1017	166.13	(L. Wang et al., 2019; L. Wang et al., 2016; Wei et al., 2016)
DC003	P-hydroxybenzoic acid	$C_7H_6O_3$	135	138.12	(Wei et al., 2016)
DC004	Vanillic acid	$C_8H_8O_4$	8468	168.15	(L. Wang et al., 2019; L. Wang et al., 2016; Wei et al., 2016)
DC005	Ferulic acid	$C_{10}H_{10}O_4$	445858	194.18	(Ding, 2016; Gao, 2016; Guan et al., 2018; Huang, 2017; Li et al., 2017; Ma et al., 2017; Pan et al., 2017; Su, 2019; Sun et al., 2019; N. Wang et al., 2017; Wei et al., 2016; G. Wu et al., 2018; Wu et al., 2016; Zhu, 2018)

No.	Derivatives and Constituents	Molecular Formula	PubChem CID/SID	Molecule Weight (g/mol)	References
DC006	Caffeic acid	$C_9H_8O_4$	689043	180.16	(Guan et al., 2018; Wei et al., 2016)
DC007	Folinic acid	$C_{20}H_{23}N_7O_7$	135403648	473.4	(Wei et al., 2016)
DC008	Folic acid	$C_{19}H_{19}N_7O_6$	135398658	441.4	(Wei et al., 2016)
DC009	Nicotinic acid	$C_6H_5NO_2$	938	123.11	(Wei et al., 2016)
DC010	Succinic acid	$C_4H_6O_4$	1110	118.09	(Wei et al., 2016)
DC011	Anisic acid	$C_8H_8O_3$	7478	152.15	(Wei et al., 2016)
DC012	Azelaic acid	$C_9H_{16}O_4$	2266	188.22	(Wei et al., 2016)
DC013	Palmitic acid	$C_{16}H_{32}O_2$	985	256.43	(X. Wang et al., 2018; Wei et al., 2016)
DC014	Lactic acid	$C_3H_6O_3$	612	90.08	(Li et al., 2017)
DC015	Acetic acid	$C_2H_4O_2$	176	60.05	(Pei et al., 2017)
DC016	Chlorogenic acid	$C_{16}H_{18}O_9$	1794427	354.31	(L. Wang et al., 2019)
DC017	Citric acid	$C_6H_8O_7$	311	192.12	(Li et al., 2017)
DC018	Formic acid	CH_2O_2	284	46.025	(Li et al., 2017)
DC019	Fumaric acid	$C_4H_4O_4$	444972	116.07	(Li et al., 2017)
DC020	Hexanoic acid	$C_6H_{12}O_2$	8892	116.16	(Li et al., 2016)
DC021	Linoleic acid	$C_{18}H_{32}O_2$	5280450	280.4	(L. Wang et al., 2019; L. Wang et al., 2016; X. Wang et al., 2018)
DC022	Linolenic acid	$C_{18}H_{30}O_2$	5280934	278.4	(J. Wang et al., 2016)
DC023	Octadecenoic acid	$C_{18}H_{34}O_2$	172146	282.5	(J. Wang et al., 2016)
DC024	Cinnamic acid	$C_9H_8O_2$	444539	444539	(Zhu, 2018)
DC025	5-Caffeoylquinic acid	$C_{16}H_{18}O_9$	12310830	354.3	(Wei, 2016)
DC026	Phytic acid	$C_6H_{18}O_{24}P_6$	890	660.04	(Filipiak-Szok et al., 2016)
**DD**	**Coumarins (n=14)**				
DD001	7H-Furo[3,2-g][1]benzopyran-7-one,9-hydroxy-	$C_{11}H_6O_4$	65090	202.16	(Jiang et al., 2019)
DD002	(+)-Marmesin	$C_{14}H_{14}O_4$	334704	246.26	(Jiang et al., 2019)
DD003	7-Hydroxy-6-methoxycoumarin	$C_{10}H_8O_4$	5280460	192.17	(Zhu, 2018)
DD004	8-Methoxypsoralen	$C_{12}H_8O_4$	4114	216.19	(Jiang et al., 2019)
DD005	Imperatorin	$C_{16}H_{14}O_4$	10212	270.28	(Jiang et al., 2019)

No.	Derivatives and Constituents	Molecular Formula	PubChem CID/SID	Molecule Weight (g/mol)	References
DD006	Alloisoimperatorin	$C_{16}H_{14}O_4$	5317436	270.28	(Jiang et al., 2019)
DD007	Isoimperatorin	$C_{16}H_{14}O_4$	68081	270.28	(Jiang et al., 2019; Xu, 2016)
DD008	Isopimpinellin	$C_{13}H_{10}O_5$	68079	246.21	(Jiang et al., 2019)
DD009	Osthenol	$C_{14}H_{14}O_3$	5320318	230.26	(Jiang et al., 2019)
DD010	Oxypeucedanin	$C_{16}H_{14}O_5$	160544	286.28	(Jiang et al., 2019)
DD011	Pabulenol	$C_{16}H_{14}O_5$	3009225	286.28	(Jiang et al., 2019)
DD012	Phellopterin	$C_{17}H_{16}O_5$	98608	300.3	(Jiang et al., 2019)
DD013	Psoralen	$C_{11}H_6O_3$	6199	186.16	(Jiang et al., 2019)
DD014	Cnidilin	$C_{17}H_{16}O_5$	821449	300.3	(Jiang et al., 2019)
**DE**	**Phospholipids (n=2)**				
DE001	1,2-di-O-hexadecanoyl-sn-glycero-3-phosphorylcholine	$C_{40}H_{80}NO_8P$	NR #	734.05	(Ma et al., 2016; Wei, 2016)
DE002	1-O-hexadecanoyl-sn-glycero-3-phosphorylcholine	$C_{24}H_{50}NO_7P$	NR #	495.64	(Ma et al., 2016; Wei, 2016)
**DF**	**Nucleosides (n=12)**				
DF001	Adenosine	$C_{10}H_{13}N_5O_4$	60961	267.25	(Ma et al., 2016; Wei, 2016; H. Zhu et al., 2017)
DF002	Uridine	$C_9H_{12}N_2O_6$	6029	244.2	(Li et al., 2017; H. Zhu et al., 2017)
DF003	Inosine	$C_{10}H_{12}N_4O_5$	6021	268.23	(S. Zhu et al., 2017)
DF004	Cytidine	$C_9H_{13}N_3O_5$	6175	243.22	(S. Zhu et al., 2017)
DF005	Thymidine	$C_{10}H_{14}N_2O_5$	5789	242.23	(S. Zhu et al., 2017)
DF006	Guanosine	$C_{10}H_{13}N_5O_5$	6802	283.24	(S. Zhu et al., 2017)
DF007	Adenosine-5′-monophosphate	$C_{10}H_{12}N_5O_7P^{-}_2$	15938965	345.21	(S. Zhu et al., 2017)
DF008	Guanosine-5′-monophosphate	$C_{10}H_{14}N_5O_8P$	135398631	363.22	(S. Zhu et al., 2017)
DF009	2′-Deoxyadenosine	$C_{10}H_{13}N_5O_3$	13730	251.24	(S. Zhu et al., 2017)
DF010	2′-Deoxyadenosine-5′-monophosphate	$C_{10}H_{14}N_5O_6P$	12599	331.22	(S. Zhu et al., 2017)
DF011	2′-Deoxyinosine	$C_{10}H_{12}N_4O_4$	135398593	252.23	(S. Zhu et al., 2017)
DF012	Cytidine-5′-monophosphate	$C_9H_{14}N_3O_8P$	6131	323.2	(S. Zhu et al., 2017)
**DG**	**Nucleobases (n=6)**				
DG001	Adenine	$C_5H_5N_5$	190	135.13	(S. Zhu et al., 2017)
DG002	Uracil	$C_4H_4N_2O_2$	1174	112.09	(S. Zhu et al., 2017)
DG003	Thymine	$C_5H_6N_2O_2$	1135	126.11	(S. Zhu et al., 2017)

No.	Derivatives and Constituents	Molecular Formula	PubChem CID/SID	Molecule Weight (g/mol)	References
DG004	Guanine	$C_5H_5N_5O$	135398634	151.13	(S. Zhu et al., 2017)
DG005	Hypoxanthine	$C_5H_4N_4O$	135398638	136.11	(S. Zhu et al., 2017)
DG006	Cytosine	$C_4H_5N_3O$	597	111.1	(S. Zhu et al., 2017)
**DH**	**Amino acids (n=17)**				
DH001	Proline	$C_5H_9NO_2$	614	115.13	(Li et al., 2017; Wei et al., 2016)
DH002	Cystine	$C_6H_{12}N_2O_4S_2$	595	240.3	(Wei et al., 2016)
DH003	Valine	$C_5H_{11}NO_2$	6287	117.15	(Li et al., 2017; Wei et al., 2016; Zhu, 2018)
DH004	Isoleucine	$C_6H_{13}NO_2$	6306	131.17	(Li et al., 2017; Wei et al., 2016; Zhu, 2018)
DH005	Phenylalanine	$C_9H_{11}NO_2$	6140	165.19	(Li et al., 2017; Wei et al., 2016; Zhu, 2018)
DH006	Asparaginic acid	$C_4H_7NO_4$	5960	133.1	(Li et al., 2017; Wei et al., 2016; Zhu, 2018)
DH007	Methionine	$C_5H_{11}NO_2S$	6137	149.208	(Li et al., 2017; Wei et al., 2016; Zhu, 2018)
DH008	Histidine	$C_6H_9N_3O_2$	6274	155.15	(Wei et al., 2016)
DH009	γ-Aminobutyric acid	$C_4H_9NO_2$	119	103.12	(Wei et al., 2016; Zhu, 2018)
DH010	Glutamic acid	$C_5H_9NO_4$	33032	147.13	(Li et al., 2017)
DH011	Alanine	$C_3H_7NO_2$	5950	89.09	(Li et al., 2017)
DH012	Arginine	$C_6H_{14}N_4O_2$	6322	174.2	(Li et al., 2017)
DH013	Threonine	$C_4H_9NO_3$	6288	119.12	(Li et al., 2017)
DH014	Tryptophan	$C_{11}H_{12}N_2O_2$	6305	204.22	(Li et al., 2017; Zhu, 2018)
DH015	Tyrosine	$C_9H_{11}NO_3$	6057	181.19	(Li et al., 2017; Zhu, 2018)
DH016	L-Proline	$C_5H_9NO_2$	145742	115.13	(S. Zhu et al., 2017)
DH017	Taurine	$C_2H_7NO_3S$	1123	125.15	(S. Zhu et al., 2017)
**DI**	**Alkaloids (n=1)**				
DI001	Banegasine	$C_{11}H_{12}N_2O_2$	NR #	204.23	(Ma et al., 2016; Wei, 2016)
**DJ**	**Vitamins (n=2)**				
DJ001	Vitamin E	$C_{29}H_{50}O_2$	14985	430.7	(X. Wang et al., 2018)

No.	Derivatives and Constituents	Molecular Formula	PubChem CID/SID	Molecule Weight (g/mol)	References
DJ002	Choline	$C_5H_{14}NO^+$	305	104.17	(Li et al., 2017)
**DK**	**Monoglycerides (n=1)**				
DK001	Glyceryl monolinoleate	$C_{21}H_{38}O_4$	5283469	354.5	(Zhu, 2018)
**DL**	**Carbohydrates (n=43)**				
DL001	α-Glucose	$C_6H_{12}O_6$	79025	180.16	(Li et al., 2017)
DL002	β-Glucose	$C_6H_{12}O_6$	64689	180.16	(Li et al., 2017)
DL003	Sucrose	$C_{12}H_{22}O_{11}$	5988	342.3	(Li et al., 2017; Ma et al., 2016; Wei, 2016)
DL004	Maltose	$C_{12}H_{22}O_{11}$	6255	342.3	(Ma et al., 2016; Wei, 2016)
DL005	As-IIIa	NR	NR	NR	(Wei et al., 2016)
DL006	As-IIIb	NR	NR	NR	(Wei et al., 2016)
DL007	X-C-3-II	NR	NR	NR	(Wei et al., 2016)
DL008	X-C-3-III	NR	NR	NR	(Wei et al., 2016)
DL009	X-C-3-IV	NR	NR	NR	(Wei et al., 2016)
DL010	XC-1	NR	NR	NR	(Wei et al., 2016)
DL011	ASP1	NR	NR	NR	(Wei et al., 2016)
DL012	ASP2	NR	NR	NR	(Wei et al., 2016)
DL013	ASP3	NR	NR	NR	(Wei et al., 2016)
DL014	APF1	NR	NR	NR	(Wei et al., 2016)
DL015	APF2	NR	NR	NR	(Wei et al., 2016)
DL016	APF3	NR	NR	NR	(Wei et al., 2016)
DL017	W-ASP11	NR	NR	NR	(Wei et al., 2016)
DL018	W-ASP12	NR	NR	NR	(Wei et al., 2016)
DL019	W-ASP-2	NR	NR	NR	(Wei et al., 2016)
DL020	W-ASP-3	NR	NR	NR	(Wei et al., 2016)
DL021	APS-1cI	NR	NR	NR	(Wei et al., 2016)
DL022	APS-1cII	NR	NR	NR	(Wei et al., 2016)
DL023	APS-1d	NR	NR	NR	(Wei et al., 2016)
DL024	ASDII-3-3	NR	NR	NR	(Wei et al., 2016)
DL025	APS-2a	NR	NR	NR	(Wei et al., 2016)
DL026	APS-bII	NR	NR	NR	(Wei et al., 2016)

No.	Derivatives and Constituents	Molecular Formula	PubChem CID/SID	Molecule Weight (g/mol)	References
DL027	APS-3a	NR	NR	NR	(Wei et al., 2016)
DL028	APS-3b	NR	NR	NR	(Wei et al., 2016)
DL029	APS-3c	NR	NR	NR	(Wei et al., 2016)
DL030	APS-1a	NR	NR	NR	(Wei et al., 2016)
DL031	ASPSF1	NR	NR	NR	(Wei et al., 2016)
DL032	ASPSF2	NR	NR	NR	(Wei et al., 2016)
DL033	ASPSF3	NR	NR	NR	(Wei et al., 2016)
DL034	ASPSF4	NR	NR	NR	(Wei et al., 2016)
DL035	ASTP	NR	NR	NR	(Wei et al., 2016)
DL036	ASTUP	NR	NR	NR	(Wei et al., 2016)
DL037	ASJP	NR	NR	NR	(Wei et al., 2016)
DL038	ASYP	NR	NR	NR	(Wei et al., 2016)
DL039	CAPS30	NR	NR	NR	(J. Wang et al., 2016)
DL040	CAPS50	NR	NR	NR	(J. Wang et al., 2016)
DL041	CAPS70	NR	NR	NR	(J. Wang et al., 2016)
DL042	CAPS80	NR	NR	NR	(J. Wang et al., 2016)
DL043	CAPSt	NR	NR	NR	(J. Wang et al., 2016)
**DM**	**Phthalic monoglycosides (n=11)**				
DM001	Ligustilide glucoside sulphate 1	$C_{21}H_{24}O_{17}S$	NR	580.48	(L. Wang et al., 2019)
DM002	Ligustilide glucoside sulphate 2	$C_{21}H_{24}O_{17}S$	NR	580.48	(L. Wang et al., 2019)
DM003	Ligustilide glucoside sulphate 3	$C_{21}H_{24}O_{17}S$	NR	580.48	(L. Wang et al., 2019)
DM004	Ligustilide glucoside sulphate 4	$C_{21}H_{24}O_{17}S$	NR	580.48	(L. Wang et al., 2019)
DM005	Phthalide derivative glucoside 1	$C_{25}H_{30}O_{13}$	NR	538.51	(L. Wang et al., 2019)
DM006	Phthalide derivative glucoside 2	$C_{25}H_{20}O_{13}$	NR	538.51	(L. Wang et al., 2019)
DM007	Phthalide derivative glucoside 3	$C_{25}H_{20}O_{13}$	NR	538.51	(L. Wang et al., 2019)
DM008	Phthalide derivative glucoside 4	$C_{24}H_{20}O_{13}$	NR	516.42	(L. Wang et al., 2019)
DM009	Phthalide derivative glucoside 5	$C_{26}H_{22}O_{13}$	NR	542.45	(L. Wang et al., 2019)
DM010	Phthalide glucoside sulphate 1	$C_{18}H_{28}O_{11}S$	NR	452.48	(L. Wang et al., 2019)
DM011	Phthalide glucoside sulphate 2	$C_{18}H_{28}O_{11}S$	NR	452.48	(L. Wang et al., 2019)
**DN**	**Elements (n=18)**				

No.	Derivatives and Constituents	Molecular Formula	PubChem CID/SID	Molecule Weight (g/mol)	References
DN001	Arsenic	As	5359596	74.922	(Qin, 2017; Rong & Zhang, 2017; H. Wang et al., 2019)
DN002	Boron	B	5462311	10.81	(H. Wang et al., 2019)
DN003	Cadmium	Cd	23973	112.414	(Rong & Zhang, 2017; H. Wang et al., 2019)
DN004	Cobalt	Co	104730	58.933	(H. Wang et al., 2019)
DN005	Chromium	Cr	23976	51.996	(Chen, 2017; Cui et al., 2017; H. Wang et al., 2019)
DN006	Copper	Cu	23978	63.546	(Chen, 2017; Cui et al., 2017; Qin et al., 2017; Qin, 2017; H. Wang et al., 2019)
DN007	Ferrum	Fe	23925	55.84	(Chen, 2017; Qin et al., 2017)
DN008	Mercury	Hg	23931	200.592	(Rong & Zhang, 2017; H. Wang et al., 2019)
DN009	Manganese	Mn	23930	54.938	(Chen, 2017; Cui et al., 2017; Qin et al., 2017; H. Wang et al., 2019)
DN010	Molybdenum	Mo	23932	95.95	(Cui et al., 2017; H. Wang et al., 2019)
DN011	Nickel	Ni	935	58.693	(Chen, 2017; H. Wang et al., 2019)
DN012	Lead	Pb	5352425	207.2	(Qin et al., 2017; Rong & Zhang, 2017; H. Wang et al., 2019)
DN013	Rubidium	Rb	5357696	85.468	(H. Wang et al., 2019)
DN014	Selenium	Se	6326970	78.971	(Chen, 2017; H. Wang et al., 2019)
DN015	Tin	Sn	5352426	118.71	(H. Wang et al., 2019)
DN016	Strontium	Sr	5359327	87.62	(H. Wang et al., 2019)
DN017	Vanadium	V	23990	50.941	(Chen, 2017; H. Wang et al., 2019)

No.	Derivatives and Constituents	Molecular Formula	PubChem CID/SID	Molecule Weight (g/mol)	References
DN018	Zinc	Zn	23994	65.379	(Chen, 2017; Cui et al., 2017; Qin et al., 2017; H. Wang et al., 2019)
**2. Fritillariae Thunbergii Bulbus**					
ZA	**Alkaloids (n=26)**				
ZA01	Peimine	$C_{27}H_{45}NO_3$	131900	431.661	(Chen et al., 2016; Duan et al., 2012; Guo, 2007; Huang, 2016; Kim et al., 2016; Ma et al., 2008)
ZA02	Peiminine	$C_{27}H_{43}NO_3$	167691	429.645	(Cheng et al., 2008; Suh et al., 2018; Xue & Gu, 2005)
ZA03	Zhebeinine	$C_{27}H_{45}NO_3$	21121503	431.661	(Zhang et al., 1993b)
ZA04	Zhebeinone	$C_{27}H_{43}NO_3$	NR #	429.645	(Zhang et al., 1992)
ZA05	Ebeiedine	$C_{27}H_{45}NO_2$	101324888	415.662	(Kim et al., 2016; Suh et al., 2018)
ZA06	Ebeiedinone	$C_{27}H_{43}NO_2$	102062796	413.646	(Suh et al., 2018; X. Wu et al., 2018)
ZA07	Isoverticine	$C_{27}H_{45}NO_3$	21573744	431.661	(Suh et al., 2018; X. Wu et al., 2018)
ZA08	Suchengbeisine	$C_{27}H_{43}NO_3$	102112537	429.645	(Kim et al., 2016; Suh et al., 2018)
ZA09	Peimisine	$C_{27}H_{41}NO_3$	161294	427.629	(Duan et al., 2012; Zhou et al., 2010)
ZA10	Peimisine-N-oxide	$C_{27}H_{42}NO_4$	NR	444.636	(Cui et al., 2016)
ZA11	Zhebeininoside	$C_{33}H_{55}NO_8$	NR #	593.802	(Ma et al., 2014; Zhou et al., 2010)
ZA12	Verticinone-3-β-D-glucoside	$C_{33}H_{53}NO_8$	90479257	591.786	(Zhou et al., 2010)
ZA13	Puqietinone	$C_{28}H_{47}NO_2$	10693900	429.689	(Zhou et al., 2010)
ZA14	Puqiedinone	$C_{27}H_{43}NO_2$	126149	413.646	(Ma et al., 2014; Zhou et al., 2010)

No.	Derivatives and Constituents	Molecular Formula	PubChem CID/SID	Molecule Weight (g/mol)	References
ZA15	Puqiedine	$C_{27}H_{45}NO_2$	101400593	415.662	(Ma et al., 2014; Zhou et al., 2010)
ZA16	Eduardine	$C_{27}H_{43}NO_2$	NR #	413.646	(Zhang et al., 1991)
ZA17	Zhebeirine	$C_{27}H_{43}NO_2$	NR #	413.646	(Zhou et al., 2017)
ZA18	2,3-deoxyladenosine	$C_{10}H_{11}N_5O_2$	NR	233.231	(Zhou et al., 2017)
ZA19	N-demethylpuqietinone	$C_{27}H_{45}NO_2$	11304576	415.662	(Zhou et al., 2017)
ZA20	Puqienine B	$C_{28}H_{45}NO_2$	11419389	443.672	(Zhou et al., 2017)
ZA21	Zhebeinone-3-β-D-glucoside	$C_{33}H_{55}NO_8$	NR #	593.802	(Zhou et al., 2017)
ZA22	Peiminoside	$C_{33}H_{55}NO_7$	90479565	577.803	(Morimoto & Kimata, 1960)
ZA23	Frithunbol A	$C_{27}H_{41}NO_4$	NR #	443.628	(Suh et al., 2018)
ZA24	Frithunbol B	$C_{27}H_{42}NO_3$	NR #	428.637	(Suh et al., 2018)
ZA25	Eduardinine	$C_{27}H_{45}NO_2$	NR #	415.662	(Suh et al., 2018)
ZA26	3β-hydroxy-5α-jervanin-12-en-6-one	$C_{27}H_{42}NO_3$	NR #	428.637	(Suh et al., 2018)
**ZB**	**Compounds from essential oils (n=28)**				
ZB01	δ-elemene	$C_{15}H_{24}$	12309449	204.357	(Cao et al., 2012)
ZB02	δ-selinene	$C_{15}H_{24}$	520383	204.357	(Cao et al., 2012)
ZB03	Tetradecanoic acid	$C_{14}H_{28}O_2$	11005	228.376	(Cao et al., 2012)
ZB04	Pentadecanoic acid	$C_{15}H_{30}O_2$	13849	242.403	(Cao et al., 2012)
ZB05	Hexadecanoic acid, methyl ester	$C_{17}H_{34}O_2$	8181	270.457	(Cao et al., 2012; Du et al., 2018)
ZB06	9-Hexadecenoic acid	$C_{16}H_{30}O_2$	5282745	254.414	(Cao et al., 2012)
ZB07	N-hexadecanoic acid	$C_{16}H_{32}O_2$	985	256.43	(Cao et al., 2012)
ZB08	Kaur-15-ene	$C_{20}H_{32}$	521318	272.476	(Cao et al., 2012)
ZB09	Heptadecanoic acid	$C_{17}H_{34}O_2$	10465	270.457	(Cao et al., 2012)
ZB10	Kaurene	$C_{20}H_{32}$	91746569	272.476	(Cao et al., 2012)
ZB11	9,12-Octadecadienoic acid (Z,Z)-, methyl ester	$C_{19}H_{34}O_2$	5284421	294.479	(Cao et al., 2012)
ZB12	9-Tetradecenal, (Z)-	$C_{14}H_{26}O$	5364471	210.361	(Cao et al., 2012)
ZB13	9,12-Octadecadienoic acid, methyl ester, (E, E)-	$C_{19}H_{34}O_2$	135058711	294.479	(Cao et al., 2012)
ZB14	Oleic acid	$C_{18}H_{34}O_2$	445639	282.468	(Cao et al., 2012)
ZB15	Linoleic acid, ethyl ester	$C_{20}H_{36}O_2$	5282184	308.506	(Cao et al., 2012)
ZB16	Butylated hydroxytoluene	$C_{15}H_{24}O$	31404	220.356	(Du et al., 2018)

No.	Derivatives and Constituents	Molecular Formula	PubChem CID/SID	Molecule Weight (g/mol)	References
ZB17	L-(+)-Ascorbic acid 2,6-dihexadecanoate	$C_{38}H_{68}O_8$	54722209	652.954	(Du et al., 2018)
ZB18	Ethyl 9-hexadecenoate	$C_{18}H_{34}O_2$	5364759	282.468	(Du et al., 2018)
ZB19	Hexadecanoic acid, ethyl ester	$C_{18}H_{36}O_2$	12366	284.484	(Du et al., 2018)
ZB20	1H-Naphtho [2,1-B] pyran, 3-ethenyldodecahydro-3,4a,7,7,10a-pentamethyl-	$C_{20}H_{34}O$	273540178	290.491	(Du et al., 2018)
ZB21	Kaur-16-ene	$C_{20}H_{32}O$	520687	272.476	(Du et al., 2018)
ZB22	9,11-Octadecadienoic acid, methyl ester, (E, E)-	$C_{19}H_{34}O_2$	319301067	294.479	(Du et al., 2018)
ZB23	9,12-Octadecadienoic acid	$C_{18}H_{32}O_2$	5282457	280.452	(Du et al., 2018)
ZB24	Octadecanoic acid, ethyl ester	$C_{20}H_{40}O_2$	8122	312.538	(Du et al., 2018)
ZB25	2(1H)-Phenanthrenone, 3,4,4a,4b,5,6,7,8,10,10a-decahydro-1,1,4a,7,7-pentamethyl, [4aR-(4a.α., 4b.β.,10a.β.)]-	$C_{19}H_{30}O$	621255	274.448	(Du et al., 2018)
ZB26	3-Methyleneandrostan-17-ol	$C_{20}H_{32}O$	625647	288.475	(Du et al., 2018)
ZB27	Androst-4-en-3-one, 17-hydroxy-, (17.β.)	$C_{19}H_{28}O_2$	50049744	288.431	(Du et al., 2018)
ZB28	Podocarp-7-en-3.β.-ol, 13.β.-methyl-13-vinyl-	$C_{20}H_{32}O$	620519	288.475	(Du et al., 2018)
**ZC**	**Diterpenoids (n=13)**				
ZC01	Isopimaran-19-ol	$C_{20}H_{32}O$	75399514	288.475	(Kitajima, Noda, et al., 1982)
ZC02	Isopimaran-19-oic acid, methyl ester	$C_{21}H_{32}O_2$	NR #	316.485	(Kitajima, Komori, et al., 1982; Kitajima, Noda, et al., 1982)
ZC03	Ent-kauran-16β, 17-diol	$C_{20}H_{34}O_2$	NR #	306.49	(Kitajima, Komori, et al., 1982; Kitajima, Noda, et al., 1982)
ZC04	Ent-kauran-16α, 17-diol	$C_{20}H_{34}O_2$	NR #	306.49	(Kitajima, Komori, et al., 1982; Kitajima, Noda, et al., 1982)
ZC05	Ent-16β, 17-epoxy-kaurane	$C_{20}H_{32}O$	79592848	288.475	(Kitajima, Noda, et al., 1982)
ZC06	Ent-16α-methoxy-kauran-17-ol	$C_{21}H_{36}O_2$	51842049	320.517	(Kitajima, Noda, et al., 1982)
ZC07	Ent-kaur-15-en-17-ol	$C_{20}H_{32}O$	3082069	288.475	(Kitajima, Noda, et al., 1982)
ZC08	Trans-communol	$C_{20}H_{32}O$	51909318	288.475	(Kitajima, Komori, et al., 1982)
ZC09	Trans-comminic acid, methyl ester	$C_{21}H_{32}O_2$	NR #	316.485	(Kitajima, Komori, et al., 1982)
ZC10	Ent-17-norkauran-16-one	$C_{19}H_{30}O$	12740861	274.448	(Kitajima, Komori, et al., 1982)
ZC11	Ent-15β,16-epoxy-kauran-17-ol	$C_{20}H_{32}O_2$	51511087	304.474	(Kitajima, Komori, et al., 1982)
ZC12	Ent-16β-hydroxy-kauran-17-yl ent-kaur-15-en-17-oate	$C_{40}H_{63}O_3$	NR #	591.941	(Kitajima, Komori, et al., 1982)

No.	Derivatives and Constituents	Molecular Formula	PubChem CID/SID	Molecule Weight (g/mol)	References
ZC13	Ent-(16S)-atisan-13, 17-oxide	$C_{20}H_{32}O$	50418337	288.475	(Kitajima, Komori, et al., 1982)
**ZD**	**Carbohydrates (n=2)**				
ZD01	β-D-glucose4-1β-D-galactose	$C_{12}H_{22}O_{11}$	NR #	342.297	(Chen & Wang, 2012)
ZD02	Sucrose	$C_{12}H_{22}O_{11}$	5988	342.297	(Chen & Wang, 2012)
**ZE**	**Sterols (n=2)**				
ZE01	β-sitosterol	$C_{29}H_{50}O$	222284	414.718	(Zhang et al., 1993b)
ZE02	Daucosterol	$C_{35}H_{60}O_6$	5742590	576.859	(Zhang et al., 1993b)
**ZF**	**Amino acids (n=18)**				
ZF01	Glycine	$C_2H_5NO_2$	750	75.067	(C. Zhang et al., 2016)
ZF02	Leucine	$C_6H_{13}NO_2$	6106	131.175	(C. Zhang et al., 2016)
ZF03	Methionine	$C_5H_{11}NO_2S$	6137	149.208	(C. Zhang et al., 2016)
ZF04	Tyrosine	$C_9H_{11}NO_3$	6057	181.191	(C. Zhang et al., 2016)
ZF05	Histidine	$C_6H_9N_3O_2$	6274	155.157	(C. Zhang et al., 2016)
ZF06	Threonine	$C_4H_9NO_3$	6288	119.12	(C. Zhang et al., 2016)
ZF07	Alanine	$C_3H_7NO_2$	5950	89.094	(C. Zhang et al., 2016)
ZF08	Isoleucine	$C_6H_{13}NO_2$	6306	131.175	(C. Zhang et al., 2016)
ZF09	Tryptophan	$C_{11}H_{12}N_2O_2$	6305	204.229	(C. Zhang et al., 2016)
ZF10	Cystine	$C_6H_{12}N_2O_4S_2$	67678	240.292	(C. Zhang et al., 2016)
ZF11	Lysine	$C_6H_{14}N_2O_2$	5962	146.19	(C. Zhang et al., 2016)
ZF12	Aspartic acid	$C_4H_7NO_4$	5960	133.103	(C. Zhang et al., 2016)
ZF13	Valine	$C_5H_{11}NO_2$	6287	117.148	(C. Zhang et al., 2016)
ZF14	Phenylalanine	$C_8H_8O_2$	6140	165.192	(C. Zhang et al., 2016)
ZF15	Proline	$C_5H_9NO_2$	145742	115.132	(C. Zhang et al., 2016)
ZF16	Serine	$C_3H_7NO_3$	5951	105.093	(C. Zhang et al., 2016)
ZF17	Glutamic acid	$C_5H_9NO_4$	33032	147.13	(C. Zhang et al., 2016)
ZF18	Arginine	$C_6H_{14}N_4O_2$	6322	174.204	(C. Zhang et al., 2016)
**ZG**	**Nucleosides (n=6)**				
ZG01	Uridine	$C_9H_{12}N_2O_6$	6029	244.203	(C. Zhang et al., 2016)
ZG02	Guanosine	$C_{10}H_{13}N_5O_5$	6802	283.244	(C. Zhang et al., 2016)
ZG03	Adenosine	$C_{10}H_{13}N_5O_4$	60961	267.245	(C. Zhang et al., 2016)
ZG04	Thymidine	$C_{10}H_{14}N_2O_5$	5789	242.231	(C. Zhang et al., 2016)

No.	Derivatives and Constituents	Molecular Formula	PubChem CID/SID	Molecule Weight (g/mol)	References
ZG05	Cytidine	$C_9H_{13}N_3O_5$	6175	243.219	(C. Zhang et al., 2016)
ZG06	Inosine	$C_{10}H_{12}N_4O_5$	6021	268.229	(C. Zhang et al., 2016)
**ZH**	**Nucleobases (n=4)**				
ZH01	Adenine	$C_5H_5N_5$	190	135.13	(C. Zhang et al., 2016)
ZH02	Hypoxanthine	$C_5H_4N_4O$	790	136.114	(C. Zhang et al., 2016)
ZH03	Uracil	$C_4H_4N_2O_2$	1174	112.088	(C. Zhang et al., 2016)
ZH04	Thymine	$C_5H_6N_2O_2$	1135	126.115	(C. Zhang et al., 2016)
**ZI**	**Fatty acids (n=4)**				
ZI01	Vernolic acid	$C_{18}H_{32}O_3$	6449780	296.451	(Zhou et al., 2017)
ZI02	2-monopalmitin	$C_{19}H_{38}O_4$	123409	330.509	(Zhou et al., 2017)
ZI03	13(R)-hydroxy-octadeca-(9Z,11E,15Z)-trien-oic acid	$C_{18}H_{30}O_3$	643726	294.435	(Zhou et al., 2017)
ZI04	Picropodophyllotoxin	$C_{22}H_{22}O_8$	72435	414.41	(Zhang et al., 1993a)
**ZJ**	**Lignans (n=3)**				
ZJ01	Octahydrocurcumin	$C_{21}H_{28}O_6$	11068834	376.449	(Zhou et al., 2017)
ZJ02	Zhebeiresinol	$C_{14}H_{16}O_6$	192547	280.276	(Jin et al., 1993; Zhou et al., 2017)
ZJ03	Sauriol B	$C_{21}H_{28}O_6$	15965508	376.449	(Zhou et al., 2017)
**ZK**	**Elements (n=27)**				
ZK01	Aluminum	Al	5359268	26.982	(Yu et al., 2007; Yuan et al., 2010)
ZK02	Arsenic	As	5359596	74.922	(Yu et al., 2007; Yuan et al., 2010)
ZK03	Boron	B	5462311	10.81	(Cai et al., 2013; Lou et al., 2014; Zhou et al., 2014)
ZK04	Barium	Ba	5355457	137.327	(Cai et al., 2013; Yuan et al., 2010)
ZK05	Bismuth	Bi	5359367	208.98	(Cai et al., 2013)
ZK06	Calcium	Ca	5460341	40.078	Yuan et al., 2010; Wang et al., 2007
ZK07	Cadmium	Cd	23973	112.414	(Cai et al., 2013; Lou et al., 2014; Zhou et al., 2014)

No.	Derivatives and Constituents	Molecular Formula	PubChem CID/SID	Molecule Weight (g/mol)	References
ZK08	Cobalt	Co	104730	58.933	(Cai et al., 2013; Yu et al., 2007)
ZK09	Chromium	Cr	23976	51.996	(Liu et al., 2008; Yu et al., 2007)
ZK10	Copper	Cu	23978	63.546	(Cai et al., 2013; Lou et al., 2014)
ZK11	Iron	Fe	23925	55.845	(Cai et al., 2013; Lou et al., 2014)
ZK12	Mercury	Hg	23931	200.592	(Cai et al., 2013; Lou et al., 2014)
ZK13	Indium	In	5359967	114.818	(Cai et al., 2013)
ZK14	Potassium	K	5462222	39.098	(Yu et al., 2007; Yuan et al., 2010)
ZK15	Lithium	Li	3028194	6.94	(Lou et al., 2014)
ZK16	Magnesium	Mg	5462224	24.305	(Lou et al., 2014; Yuan et al., 2010)
ZK17	Manganese	Mn	23930	54.938	(Cai et al., 2013; Lou et al., 2014)
ZK18	Molybdenum	Mo	23932	95.95	(Yuan et al., 2010)
ZK19	Sodium	Na	5360545	22.99	(Yu et al., 2007; Yuan et al., 2010)
ZK20	Nickel	Ni	935	58.693	(Cai et al., 2013; Yuan et al., 2010)
ZK21	Phosphorus	P	5462309	30.974	(Yuan et al., 2010)
ZK22	Lead	Pb	5352425	207.2	(Cai et al., 2013; Lou et al., 2014)
ZK23	Sulphur	S	5362487	32.06	(Lou et al., 2014)
ZK24	Selenium	Se	6326970	78.971	(Wu et al., 1998)
ZK25	Strontium	Sr	5359327	87.62	(Lou et al., 2014; Yu et al., 2007)
ZK26	Vanadium	V	23990	50.941	(Wu & Zheng, 1997)

No.	Derivatives and Constituents	Molecular Formula	PubChem CID/SID	Molecule Weight (g/mol)	References
ZK27	Zinc	Zn	23994	65.379	(Cai et al., 2013; Lou et al., 2014)
**3. Sophorae Flavescentis Radix**					
**KA**	**Flavonoids (n=179)**				
KA001	Quercetin	$C_{15}H_{10}O_7$	5280343	302.23	(He et al., 2015; Weng et al., 2018)
KA002	Rutin	$C_{27}H_{30}O_{16}$	5280805	610.5	(He et al., 2015; Lee et al., 2018; Weng et al., 2018)
KA003	Kushenol C	$C_{25}H_{26}O_7$	5481237	438.5	(He et al., 2015; Kim et al., 2018)
KA004	Methylkushenol C	$C_{26}H_{28}O_7$	NR #	452.5	(He et al., 2015)
KA005	Kushenol G	$C_{25}H_{28}O_8$	44259516	456.5	(He et al., 2015; Li et al., 2019)
KA006	8-Prenylkaempferol	$C_{20}H_{18}O_6$	5318624	354.4	(He et al., 2015; Kim et al., 2018; Li et al., 2019)
KA007	Isoanhydroicaritin	$C_{21}H_{20}O_6$	5322079	368.4	(He et al., 2015; Z. Yang et al., 2016)
KA008	Sophoflavescenol	$C_{21}H_{20}O_6$	9929189	368.4	(He et al., 2015)
KA009	Resokaempferol	$C_{15}H_{10}O_5$	5281611	270.24	(He et al., 2015)
KA010	8-Lavandulylkaempferol	$C_{25}H_{26}O_6$	16083184	422.5	(He et al., 2015; Li et al., 2019)
KA011	8-Lavandulyl-5,7,4'-trihydroxy-flavonol	$C_{25}H_{26}O_6$	NR #	422.5	(He et al., 2015)
KA012	Citrusinol	$C_{20}H_{16}O_6$	44259051	352.3	(He et al., 2015)
KA013	Flavenochromane B	$C_{25}H_{26}O_6$	273676818	422.5	(He et al., 2015)
KA014	Flavenochromane C	$C_{21}H_{20}O_6$	273676819	368.4	(He et al., 2015)
KA015	Kaempferol	$C_{15}H_{10}O_6$	5280863	286.24	(He et al., 2015; Lee et al., 2018)
KA016	Catechin	$C_{15}H_{14}O_6$	9064	290.27	(Lee et al., 2018)
KA017	Epicatechin	$C_{15}H_{14}O_6$	72276	290.27	(Lee et al., 2018)
KA018	Noranhyoicaritin	$C_{20}H_{18}O_6$	NR #	354.36	(Zhao, 2015)
KA019	Lanceolarin	$C_{27}H_{30}O_{14}$	5492234	578.5	(Zhao, 2015)

No.	Derivatives and Constituents	Molecular Formula	PubChem CID/SID	Molecule Weight (g/mol)	References
KA020	Kushenol A	$C_{25}H_{28}O_5$	44563121	408.5	(Kim et al., 2017; Kim et al., 2018; Weng et al., 2018; Zhao, 2015)
KA021	Kushenol B	$C_{30}H_{36}O_6$	5318891	492.6	(He et al., 2015; Kim et al., 2017; Li et al., 2019)
KA022	Kushenol E	$C_{25}H_{28}O_6$	9979767	424.5	(He et al., 2015; Kim et al., 2017; Zhao, 2015)
KA023	Kushenol F	$C_{25}H_{28}O_6$	10455036	424.5	(He et al., 2015)
KA024	Kushenol P	$C_{26}H_{32}O_7$	10742453	456.5	(He et al., 2015; Zhao, 2015)
KA025	Kushenol Q	$C_{25}H_{30}O_7$	NR #	442.51	(He et al., 2015; Li et al., 2019)
KA026	Kushenol R	$C_{26}H_{30}O_5$	42607847	422.5	(He et al., 2015; Li et al., 2019)
KA027	Kushenol S	$C_{20}H_{20}O_5$	10854625	340.4	(He et al., 2015; Li et al., 2019)
KA028	Kushenol T	$C_{25}H_{30}O_6$	10598514	426.5	(He et al., 2015; Li et al., 2019)
KA029	Kushenol U	$C_{26}H_{30}O_5$	42608062	422.5	(He et al., 2015; Li et al., 2019)
KA030	Kushenol V	$C_{21}H_{22}O_7$	10572194	386.4	(He et al., 2015)
KA031	Kushenol W	$C_{21}H_{22}O_7$	42608033	386.4	(He et al., 2015)
KA032	Kurarinone	$C_{26}H_{30}O_6$	11982640	438.5	(He et al., 2015; Q. Huang et al., 2017; Sun et al., 2017; Weng et al., 2018; Z. Yang et al., 2016; Q. Zhang, J. Yu, Y. Wang, et al., 2016; Zhao, 2015)
KA033	2'-methoxykurarinone	$C_{27}H_{32}O_6$	133561937	452.5	(He et al., 2015; Li et al., 2019; Zhao, 2015)
KA034	Isokurarinone	$C_{26}H_{30}O_6$	5318581	438.5	(He et al., 2015; Zhao, 2015)
KA035	Norkurarinone	$C_{25}H_{28}O_6$	273580998	424.5	(He et al., 2015; Li et al., 2019; Zhao, 2015)
KA036	Kurarinol	$C_{26}H_{32}O_7$	44563198	456.5	(He et al., 2015; Q. Zhang, J. Yu, Y. Wang, et al., 2016; Zhao, 2015)
KA037	Neokurarinol	$C_{27}H_{34}O_7$	102090469	470.6	(He et al., 2015; Li et al., 2019)
KA038	Norkurarinol	$C_{25}H_{30}O_7$	44563159	442.5	(He et al., 2015; Li et al., 2019)

No.	Derivatives and Constituents	Molecular Formula	PubChem CID/SID	Molecule Weight (g/mol)	References
KA039	Isoxanthohumol	$C_{21}H_{22}O_5$	513197	354.4	(He et al., 2015; Q. Huang et al., 2017; Li et al., 2019; Sun et al., 2017; Z. Yang et al., 2016; Zhao, 2015)
KA040	Leachianone A	$C_{26}H_{30}O_6$	44593449	438.5	(He et al., 2015)
KA041	Leachianone G	$C_{20}H_{20}O_6$	5275227	356.4	(He et al., 2015)
KA042	Sophoraflavanone B	$C_{20}H_{20}O_5$	252448649	340.4	(He et al., 2015; Li et al., 2019; Zhao, 2015)
KA043	5-Methylsophoraflavanone B	$C_{21}H_{22}O_5$	273518150	354.4	(He et al., 2015)
KA044	Sophoraflavanone G	$C_{25}H_{28}O_6$	72936	424.5	(He et al., 2015; Q. Huang et al., 2017; Sun et al., 2017; Weng et al., 2018; Z. Yang et al., 2016; Zhao, 2015)
KA045	Sophoraflavanone K	$C_{26}H_{30}O_7$	NR	454.5	(He et al., 2015; Zhao, 2015)
KA046	Sophoraflavanone L	$C_{25}H_{28}O_6$	16083180	424.5	(He et al., 2015)
KA047	Naringenin	$C_{15}H_{12}O_5$	932	272.25	(He et al., 2015)
KA048	Naringenin-7-O-β-D-glucosyl-4'-O-β-D-glucose	NR	NR	NR	(He et al., 2015)
KA049	5-Methoxy-7,2',4'-trihydroxy-8-[3,3-dimethylallyl]-flavanone	$C_{21}H_{22}O_6$	NR #	370.4	(He et al., 2015)
KA050	(2S)-6-lavandulyl-isopentenyl-5-methoxy-7,2',4',-trihydroxy-flavonone	$C_{25}H_{28}O_6$	NR #	424.5	(He et al., 2015)
KA051	(2S)-8-isopentenyl-7,2',4',-5-methoxyflavonone	$C_{21}H_{22}O_6$	NR #	370.4	(He et al., 2015)
KA052	(2S)-6[2(3-hydroxyisopropyl)-5-methyl-4-hexenyl]-5-methoxy-7,2',4'-trihydroxy flavanone	$C_{26}H_{32}O_7$	NR #	456.54	(He et al., 2015)
KA053	(2S)-5, 4'-dimethoxy-8-lavandulyl-7,2'-dihydroxy flavanone	$C_{27}H_{32}O_6$	NR #	452.55	(He et al., 2015)
KA054	(2S)-8-(5-hydroxy-2-isopropenyl-5-methylhexyl)-7-methoxy-5,2', 4'-trihydroxy-flavanone	$C_{26}H_{32}O_7$	NR #	456.54	(He et al., 2015)
KA055	(2S)-7,4'-dihydroxy-5-methoxy-8-(γ, γ-dimethylallyl)-flavanone	$C_{21}H_{22}O_5$	NR #	354.4	(He et al., 2015)

No.	Derivatives and Constituents	Molecular Formula	PubChem CID/SID	Molecule Weight (g/mol)	References
KA056	(2R)-3α,7,4'-trihydroxy-5-methoxy-8-(γ,γ-dimethylallyl)-flavanone	$C_{21}H_{22}O_6$	NR #	370.4	(He et al., 2015)
KA057	(2S)-3β,7,4'-trihydroxy-5-methoxy-8-(γ,γ-dimethylally)-flavanone	$C_{21}H_{22}O_6$	NR #	370.4	(He et al., 2015)
KA058	(2S)-7,2',4'-trihydroxy-5-methoxy-8-dimethylallylflavanone	$C_{21}H_{22}O_6$	NR #	370.4	(He et al., 2015)
KA059	3β,7,4'-Trihydroxy-5-methoxy-8-[3,3-dimethylallyl]-flavanone	$C_{21}H_{22}O_6$	NR #	370.4	(He et al., 2015)
KA060	4'-Hydroxyisolonchocarpin	$C_{20}H_{18}O_4$	14353465	322.4	(He et al., 2015)
KA061	5'-Hydroxy norkurarinol	$C_{25}H_{28}O_8$	NR #	456.49	(Zhao, 2015)
KA062	7,4"-Dihydroxy-8-(3-methylisobutanol)flavanone	$C_{20}H_{20}O_5$	NR #	340.38	(Zhao, 2015)
KA063	7-Hydroxy-3″-methoxy-isoflavone-7-primeveroside	$C_{27}H_{30}O_{13}$	NR	562.52	(Li et al., 2019)
KA064	Sophoraisoflavone C	$C_{30}H_{34}O_5$	NR #	474.6	(Zhao, 2015)
KA065	Sophoraisoflavone D	$C_{30}H_{34}O_6$	NR #	490.6	(Zhao, 2015)
KA066	Sophoraisoflavone A	$C_{20}H_{16}O_6$	10383349	352.3	(Wu, 2016; Zhao, 2015)
KA067	Kushenol J	$C_{32}H_{41}O_{18}$	NR #	713.66	(He et al., 2015; Zhao, 2015)
KA068	Kushenol H	$C_{26}H_{32}O_8$	NR #	472.53	(He et al., 2015)
KA069	Kushenol I	$C_{25}H_{28}O_7$	NR #	440.49	(He et al., 2015; R. Huang et al., 2017; Li et al., 2019; Q. Zhang, J. Yu, Y. Wang, et al., 2016)
KA070	Kushenol K	$C_{26}H_{32}O_8$	5318897	472.5	(He et al., 2015)
KA071	Kushenol L	$C_{25}H_{28}O_7$	21721878	440.5	(He et al., 2015; Kim et al., 2017; Li et al., 2019; Zhao, 2015)
KA072	Kushenol M	$C_{30}H_{36}O_7$	180948	508.6	(He et al., 2015; Zhao, 2015)
KA073	Kushenol N	$C_{26}H_{30}O_7$	381851	454.5	(He et al., 2015; R. Huang et al., 2017; Li et al., 2019; Weng et al., 2018; Q. Zhang, J. Yu, Y. Wang, et al., 2016)
KA074	Kushenol X	$C_{25}H_{28}O_7$	10599228	440.5	(He et al., 2015; Li et al., 2019)

No.	Derivatives and Constituents	Molecular Formula	PubChem CID/SID	Molecule Weight (g/mol)	References
KA075	Kosamol A	$C_{30}H_{38}O_8$	275852942	526.6	(He et al., 2015)
KA076	(2R, 3R)-8-isopentenyl-7,4'-dihydroxy -5-methoxy-flavanonol	$C_{21}H_{22}O_6$	NR #	370.4	(He et al., 2015; Li et al., 2019)
KA077	(2R, 3R)-8-lavanduly-5,7,4'-trihydroxy-2'-methoxy-flavanonol	$C_{26}H_{30}O_7$	NR #	454.5	(He et al., 2015)
KA078	(2R, 3R)-8-isopentenyl-7,2',4'-trihydroxy-5-methoxy-flavanonol	$C_{21}H_{22}O_7$	NR #	386.4	(He et al., 2015)
KA079	Flavenochromane A	$C_{25}H_{28}O_7$	273676817	440.49	(He et al., 2015)
KA080	Formononetin	$C_{16}H_{12}O_4$	5280378	268.26	(He et al., 2015; Q. Huang et al., 2017; Kim et al., 2018; Li et al., 2019; Z. Yang et al., 2016)
KA081	Daidzein	$C_{15}H_{10}O_4$	5281708	254.24	(He et al., 2015; Weng et al., 2018; Zhao, 2015)
KA082	Biochanin A	$C_{16}H_{12}O_5$	5280373	284.26	(He et al., 2015; Weng et al., 2018; Zhao, 2015)
KA083	7-Methoxy-4'-hydroxyisoflavone	$C_{12}H_{16}O_4$	NR #	224.26	(He et al., 2015)
KA084	7,4'-Dihydroxy-3'-methoxyisoflavone	$C_{16}H_{12}O_5$	160709470	284.26	(He et al., 2015)
KA085	Calycosin	$C_{16}H_{12}O_5$	5280448	284.26	(He et al., 2015; Q. Huang et al., 2017; Sun et al., 2017; Zhao, 2015)
KA086	Kushenol O	$C_{27}H_{30}O_{13}$	44257224	562.5	(He et al., 2015; Li et al., 2019)
KA087	3’-Hydroxykushenol O	NR	NR	NR	(He et al., 2015)
KA088	Ononin	$C_{22}H_{22}O_9$	442813	430.4	(He et al., 2015)
KA089	Pseudobatigenin	$C_{16}H_{10}O_5$	NR #	282.25	(He et al., 2015)
KA090	Pseudobatigenin-7-O-β-D-xylose-(1→6)-β-D-glucopyranoside	$C_{27}H_{24}O_{14}$	NR #	576.51	(He et al., 2015)
KA091	Daidzein-7-O-β-D-xylose-(1→6)-β-D-glucopyranoside	$C_{26}H_{28}O_{13}$	NR #	548.5	(He et al., 2015)
KA092	3'-Methoxy-4'-hydroxyisoflavone-7-O-β-D-xylose-(1→6)-β-D-glucopyranoside	$C_{27}H_{30}O_{14}$	NR #	578.52	(He et al., 2015)
KA093	3'-Hydroxy-4'-methoxyisoflavone-7-O-β-D-xylopyranosyl-(1→6)-β-D-glucopyranoside	$C_{27}H_{30}O_{14}$	NR #	578.52	(He et al., 2015)

No.	Derivatives and Constituents	Molecular Formula	PubChem CID/SID	Molecule Weight (g/mol)	References
KA094	3'-Hydroxy-4'-methoxyisoflavone-7-O-β-D-apiofuranosyl-(1→6)-β-D-glucopyranoside	$C_{27}H_{30}O_{14}$	NR #	578.52	(He et al., 2015)
KA095	3'-Methoxy-4'-hydroxyisoflavone-7-O-β-D-apiose-(1-6)-β-D-glucopyranoside	$C_{27}H_{30}O_{14}$	NR #	578.52	(He et al., 2015)
KA096	4'-Hydroxy-3'-methoxyisoflavone-7-O-β-D-xylopyranosyl-(1→6)-β-D-glucopyranoside	$C_{27}H_{30}O_{14}$	NR #	578.52	(He et al., 2015)
KA097	4'-Hydroxy-3'-methoxyisoflavone-7-O-β-D-apiofuranosyl-(l→6)-β-D-glucopyranoside	$C_{27}H_{30}O_{14}$	NR #	578.52	(He et al., 2015)
KA098	4'-Hydroxyisoflavone-7-O-β-D-apiose-(1→6)-β-D-glucopyranoside	$C_{26}H_{28}O_{13}$	NR #	548.5	(He et al., 2015)
KA099	4'-Methoxyisoflavone-7-O-β-D-apiose-(1→6) - β-D-glucopyranoside	$C_{27}H_{30}O_{13}$	NR #	562.52	(He et al., 2015)
KA100	3',4'-Methylenedioxyisoflavone-7-O-β-D-apiofuranosyl-(l→6)-β-D-glucopyranoside	$C_{27}H_{28}O_{14}$	NR #	576.51	(He et al., 2015)
KA101	3',4'-Dihydroxy-isoflavone-7-O-β-D-glucopyranoside	$C_{21}H_{20}O_{10}$	NR #	632.38	(He et al., 2015)
KA102	5-Hydroxy-4'-methoxyisoflavone-7-O-β-D-xylose-(1→6) -β-D-glucopyranoside	$C_{27}H_{30}O_{14}$	NR #	578.52	(He et al., 2015)
KA103	5-Hydroxy-4'-methoxyisoflavone-7-O-β-D-apiose-(1→6)-β-D-glucopyranoside	$C_{27}H_{30}O_{14}$	NR #	578.52	(He et al., 2015)
KA104	5'-Hydroxy-4'-methoxyisoflavone-2'-β-D-glucopyranoside	$C_{22}H_{22}O_{12}$	NR #	478.41	(He et al., 2015)
KA105	5,4'-Dihydroxy-isoflavone-7-O-β-D-xylose-(1→6)-β-D-glucopyranoside	$C_{26}H_{28}O_{14}$	NR #	564.5	(He et al., 2015)
KA106	Calycosin-7-glucoside	$C_{22}H_{22}O_{10}$	71571502	446.4	(Weng et al., 2018)
KA107	4'-Hydroxyisoflavone-7-O-β-D-xylose-(1→6)-glucopyranoside	$C_{26}H_{28}O_{13}$	NR #	548.5	(Zhao, 2015)
KA108	5,7,2',5'-Tetrahydroxy-4'-methoxy-6-lavandulyl-8-(3-methyl-2-butene)-isoflavone	$C_{31}H_{38}O_{7}$	NR #	522.64	(Zhao, 2015)
KA109	5,7,4'-Trihydroxy-3'-methoxy-6,5'-di(γ,γ-dimethylallyl)-isoflavone	$C_{26}H_{30}O_{6}$	NR #	438.52	(Zhao, 2015)

No.	Derivatives and Constituents	Molecular Formula	PubChem CID/SID	Molecule Weight (g/mol)	References
KA110	5,7-Dimethoxy-4',5',-methylenedioxy-8,3'-di(3-methy-4-hydroxy-2-buten)flavanone	$C_{28}H_{32}O_6$	NR #	464.56	(Zhao, 2015)
KA111	5-Methoxykushenol C	NR	NR	NR	(Li et al., 2019)
KA112	7-Hydroxy-3″-methoxyisoflavone-7-primeveroside	NR	NR	NR	(Li et al., 2019)
KA113	Kakkanin	$C_{27}H_{30}O_{14}$	273966287	578.5	(Zhao, 2015)
KA114	Orobol	$C_{15}H_{10}O_6$	5281801	286.24	(Zhao, 2015)
KA115	Pseudobatigenin-7-O-β-D-apiofuranosyl-(1→6)-β-D-glucopyranosid	$C_{27}H_{28}O_{14}$	NR #	576.51	(Zhao, 2015)
KA116	Pseudobatioenin	$C_{16}H_{12}O_5$	NR #		(Zhao, 2015)
KA117	7-Hydroxy-4'-hydroxyisoflavonone-3'-O-β-D-glucopyranoside	NR	NR	NR	(He et al., 2015)
KA118	2,3-Dihydroxy-4'-methoxyisoflavonone-7-O-β- D-xylose-(1→6)-β-D-glucopyranoside	NR	NR	NR	(He et al., 2015)
KA119	2,3-Dihydroxy-4'-methoxyisoflavonone-7-O-β-D-apiose-(1→6)-β-D-glucopyranoside	NR	NR	NR	(He et al., 2015)
KA120	Medicarpin	$C_{16}H_{14}O_4$	336327	270.28	(Takahashi et al., 2016)
KA121	5-Methoxy-isoflavone-7-O-β-D-glucopyranoside	$C_{22}H_{22}O_9$	NR #	430.41	(Zhao, 2015)
KA122	5-Methoxy-isoflavone-7-di-O-β-D-glucopyranoside	$C_{28}H_{32}O_{14}$	NR #	592.55	(Zhao, 2015)
KA123	Genistein-7-rutinoside	NR	NR	NR	(Zhao, 2015)
KA124	2,3,4'-Trihydroxy-homoisoflavone-7-O-β-D-glucopyranoside	NR	NR	NR	(He et al., 2015)
KA125	2,3-Dihydroxy-4'-methoxyhomoisoflavone-7-O-xyloside	NR	NR	NR	(He et al., 2015)
KA126	2',4-Dihydroxy-4',6'-dimethoxychalcone	$C_{17}H_{16}O_5$	277363800	300.3	(He et al., 2015)
KA127	Kuraridin	$C_{26}H_{30}O_6$	9954815	438.5	(He et al., 2015; Li et al., 2019; Sun et al., 2017; Z. Yang et al., 2016)
KA128	Kuraridinol	$C_{26}H_{32}O_7$	5318880	456.5	(He et al., 2015; Li et al., 2019)
KA129	Kushenol D	$C_{27}H_{32}O_6$	5318893	452.5	(He et al., 2015; Li et al., 2019)
KA130	Xanthohumol	$C_{21}H_{22}O_5$	639665	354.4	(He et al., 2015; Li et al., 2019; Sun et al., 2017; Q. Wang et al., 2017)

No.	Derivatives and Constituents	Molecular Formula	PubChem CID/SID	Molecule Weight (g/mol)	References
KA131	Cyclokuraridin	$C_{26}H_{30}O_6$	16083181	438.5	(He et al., 2015)
KA132	(Z)-4,2',4'-trihydroxy chalcone	$C_{15}H_{12}O_4$	NR #	256.26	(He et al., 2015)
KA133	Sophflavone A	$C_{52}H_{58}O_{12}$	NR #	875.02	(He et al., 2015)
KA134	Sophflavone B	$C_{52}H_{56}O_{12}$	NR #	873.01	(He et al., 2015)
KA135	Kushecarpin A	$C_{17}H_{18}O_6$	272895690	318.32	(He et al., 2015)
KA136	Kushecarpin B	$C_{18}H_{18}O_7$	272895691	346.3	(He et al., 2015)
KA137	Kushecarpin C	$C_{17}H_{16}O_7$	272895692	332.3	(He et al., 2015)
KA138	Kushecarpin D	$C_{16}H_{14}O_6$	275167221	302.28	(He et al., 2015)
KA139	Maackiain	$C_{16}H_{12}O_5$	91510	284.26	(He et al., 2015; Q. Huang et al., 2017; Lee et al., 2018; Li et al., 2019; Takahashi et al., 2016; Weng et al., 2018; Zhao, 2015)
KA140	4-Methoxy-maackiain	NR	NR	NR	(He et al., 2015)
KA141	Maackiain-7-O-β-D-apiose-(1→6)-β-D-glucopyranoside	NR	NR	NR	(He et al., 2015)
KA142	Pterocarpin	$C_{17}H_{14}O_5$	1715306	298.29	(He et al., 2015)
KA143	3-hydroxy-4-methoxy-8,9-methylenedioxypterocarpan	NR	NR	NR	(He et al., 2015)
KA144	Trifolirhizin	$C_{22}H_{22}O_{10}$	442827	446.4	(He et al., 2015; Q. Huang et al., 2017; Sun et al., 2017; Weng et al., 2018; Z. Yang et al., 2016)
KA145	Trifolirhizin-6'-monoacetate	$C_{24}H_{24}O_{11}$	44257443	488.4	(He et al., 2015; Q. Huang et al., 2017; Zhao, 2015)
KA146	Kushenin	$C_{16}H_{14}O_5$	5318889	286.28	(He et al., 2015)
KA147	Medicarpin-3-O-β-D-apiose-(1→6)-β-D- glucopyranoside	NR	NR	NR	(He et al., 2015)
KA148	Sophoraflavanone M	$C_{20}H_{20}O_5$	NR #	340.38	(Ma et al., 2019)
KA149	Sophoraflavanone N	$C_{25}H_{28}O_5$	NR #	408.49	(Ma et al., 2019)
KA150	Glabranin	$C_{20}H_{20}O_4$	124049	324.4	(Ma et al., 2019)
KA151	(2S)-7-hydroxy-5-methoxy-8-prenylflavanone	$C_{21}H_{22}O_4$	NR #	338.4	(Ma et al., 2019)
KA152	(2S)-8-[2-(3-hydroxyisopropyl)-5-methyl-4- hexenyl]-2′-methoxy-5,7,4′-trihydroxyflavanone	$C_{26}H_{32}O_7$	NR #	456.54	(Ma et al., 2019)

No.	Derivatives and Constituents	Molecular Formula	PubChem CID/SID	Molecule Weight (g/mol)	References
KA153	Leachianone B	$C_{26}H_{30}O_6$	15719490	438.5	(Ma et al., 2019)
KA154	3,7-Dihydroxycoumarin	$C_9H_6O_4$	6918810	178.14	(R. Huang et al., 2017)
KA155	8-(3-Hydroxymethyl-2-butenyl)-5,7,2′,4′-tetrahydroxy-flavanone	$C_{20}H_{20}O_7$	NR #	372.37	(R. Huang et al., 2017)
KA156	8-Lavandulyl-5,7,4′-trihydroxyflavonol	$C_{25}H_{26}O_6$	NR #	422.48	(Q. Huang et al., 2017)
KA157	8-Prenylnaringenin	$C_{20}H_{20}O_5$	480764	340.4	(Kim et al., 2018)
KA158	Luteolin	$C_{15}H_{10}O_6$	5280445	286.24	(Weng et al., 2018)
KA159	Daidzin	$C_{21}H_{20}O_9$	107971	416.4	(Weng et al., 2018)
KA160	Luteoloside	$C_{21}H_{20}O_{11}$	162188761	448.4	(Weng et al., 2018)
KA161	Isoquercitrin	$C_{21}H_{20}O_{12}$	5280804	464.4	(Weng et al., 2018)
KA162	Myricetin	$C_{15}H_{10}O_8$	5281672	318.23	(Weng et al., 2018)
KA163	(2R)-3α,7,4′-Trihydroxy-5-methoxy-8-prenylflavanone	$C_{21}H_{22}O_6$	NR #	370.4	(R. Huang et al., 2017)
KA164	(2R)-3β,7,4′-Trihydroxy-5-methoxy-8-prenylflavanone	$C_{21}H_{22}O_6$	NR #	370.4	(R. Huang et al., 2017)
KA165	Shandougenines B	$C_{30}H_{18}O_{10}$	NR #	538.46	(R. Huang et al., 2017)
KA166	Specionin	$C_{20}H_{26}O_8$	10982109	394.4	(R. Huang et al., 2017)
KA167	2'-Hydroxyisoxanthohumol	$C_{21}H_{22}O_6$	NR	370.4	(Li et al., 2019)
KA168	Euchrestaflavanone A	$C_{25}H_{28}O_5$	484588	408.5	(Zhao, 2015)
KA169	Exiguaflavanone B	$C_{26}H_{30}O_6$	10455800	438.5	(Zhao, 2015)
KA170	Exiguaflavanone K	$C_{21}H_{22}O_6$	11036041	370.4	(Zhao, 2015)
KA171	Diosmetin	$C_{16}H_{12}O_6$	5281612	300.26	(Zhao, 2015)
KA172	7,3'-Dihydroxy-4'-methoxyflavone	$C_{16}H_{12}O_5$	5378220	284.26	(Zhao, 2015)
KA173	6"-Apioside-genistin	$C_{26}H_{28}O_{14}$	NR #	564.5	(Zhao, 2015)
KA174	Kurarinol C	$C_{25}H_{26}O_7$	NR #	438.48	(Zhao, 2015)
KA175	Kurarinol O	$C_{27}H_{30}O_{14}$	NR #	578.52	(Zhao, 2015)
KA176	Genistin	$C_{21}H_{20}O_{10}$	5281377	432.4	(Zhao, 2015)
KA177	Genistein	$C_{15}H_{10}O_5$	5280961	270.24	(Zhao, 2015)
KA178	Desmethylxanthohumol	$C_{20}H_{20}O_5$	6443339	340.4	(Li et al., 2019)
KA179	Trifolirhizin-6"-O-malonate	$C_{25}H_{24}O_{13}$	14841223	532.4	(Zhao, 2015)
**KB**	**Alkaloids (n=50)**				

No.	Derivatives and Constituents	Molecular Formula	PubChem CID/SID	Molecule Weight (g/mol)	References
KB001	Matrine	$C_{15}H_{24}N_2O$	91466	248.36	(Chen et al., 2017; Guan et al., 2015; Hao et al., 2017; He et al., 2015; Ji et al., 2017; Li et al., 2019; Sun et al., 2017; B. Wang et al., 2018; C. Wang et al., 2017; Wu et al., 2017; Wu, 2016; Y. Yang et al., 2016; R. Zhang et al., 2016; Zhang et al., 2015; Y. B. Zhang et al., 2016; D. Zhao et al., 2018)
KB002	Isomatrine	$C_{15}H_{24}N_2O$	5271984	248.36	(He et al., 2015)
KB003	(+)-Allomatrine	$C_{15}H_{24}N_2O$	7000681	248.36	(He et al., 2015)
KB004	Cis-neomatrine	$C_{15}H_{24}N_2O$	273503408	248.36	(He et al., 2015)
KB005	Trans-neomatrine	$C_{15}H_{24}N_2O$	273503409	248.36	(He et al., 2015)
KB006	(-)-9α-Hydroxy-7,11-dehydromatrine	$C_{15}H_{22}N_2O_2$	NR #	262.35	(He et al., 2015)
KB007	Oxymatrine	$C_{15}H_{24}N_2O_2$	114850	264.36	(Chen et al., 2017; Guan et al., 2015; Hao et al., 2017; He et al., 2015; Li et al., 2019; Sun et al., 2017; C. Wang et al., 2017; Wu, 2016; Y. Yang et al., 2016; Q. Zhang, J. Yu, L. Zhang, et al., 2016; Zhang et al., 2015; D. Zhao et al., 2018; Zhao, 2015)
KB008	(+)-9α-Hydroxymatrine	$C_{15}H_{24}N_2O_2$	103481335	264.36	(He et al., 2015; D. Zhao et al., 2018)
KB009	(-)-14β-Hydroxymatrine	$C_{15}H_{24}N_2O_2$	274945414	264.36	(He et al., 2015)
KB010	(+)-Sophoranol	$C_{15}H_{24}N_2O_2$	12442899	264.36	(He et al., 2015; Ji et al., 2017; D. Zhao et al., 2018)
KB011	(+)-Sophoranol N-oxide	NR	NR	NR	(He et al., 2015)
KB012	(+)-Oxysophoranol N-oxide	NR	NR	NR	(He et al., 2015)
KB013	13,14-Dehydroxysophoridine	$C_{15}H_{24}N_2O$	NR #	248.36	(He et al., 2015)

No.	Derivatives and Constituents	Molecular Formula	PubChem CID/SID	Molecule Weight (g/mol)	References
KB014	(-)-Sophocarpine	$C_{15}H_{22}N_2O$	115269	246.35	(He et al., 2015; Ji et al., 2017; Li et al., 2019; Sun et al., 2017; C. Wang et al., 2017; Wu et al., 2017; Zhang et al., 2015; D. Zhao et al., 2018; Zhao, 2015)
KB015	Oxysophocarpine	$C_{15}H_{22}N_2O_2$	24721085	262.35	(He et al., 2015; Ji et al., 2017; Li et al., 2019; C. Wang et al., 2017; Q. Zhang, J. Yu, L. Zhang, et al., 2016; Zhang et al., 2015; D. Zhao et al., 2018; Zhao, 2015)
KB016	Sophocarpine, N-oxide	$C_{15}H_{22}N_2O_2$	250098840	262.35	(He et al., 2015)
KB017	(-)-9α-Hydroxysophocarpine	$C_{15}H_{24}N_2O$	355353482	248.36	(Li et al., 2019)
KB018	9α-Hydroxysophocarpine N-oxide	NR	NR	NR	(He et al., 2015)
KB019	(+)-12α-Hydroxysophocarpine	$C_{15}H_{22}N_2O_2$	44408595	262.35	(He et al., 2015)
KB020	Sophoramine	$C_{15}H_{20}N_2O$	169014	244.33	(He et al., 2015; Li et al., 2019; Sun et al., 2017)
KB021	(-)-13-Ethylsophoramine	NR	NR	NR	(He et al., 2015)
KB022	Tetrahydroneosophoramine	NR	NR	NR	(He et al., 2015)
KB023	(-)-Cytisine	$C_{11}H_{14}N_2O$	10235	190.24	(He et al., 2015; Li et al., 2019; Zhao, 2015)
KB024	N-Methylcytisine	$C_{12}H_{16}N_2O$	670971	204.27	(He et al., 2015; Li et al., 2019; Sun et al., 2017; Zhang et al., 2015; Zhao, 2015)
KB025	(-)-Baptifoline	$C_{15}H_{20}N_2O_2$	621307	260.33	(He et al., 2015; Li et al., 2019; Sun et al., 2017; Zhao, 2015)
KB026	(-)-Anagyrine	$C_{15}H_{20}N_2O$	10246	244.33	(He et al., 2015; Sun et al., 2017; D. Zhao et al., 2018; Zhao, 2015)
KB027	Flavascensine	$C_{12}H_{20}N_2O$	NR #	208.31	(He et al., 2015)
KB028	2-Oxymatrine	$C_{15}H_{22}N_2O_2$	NR #	262.35	(Y. B. Zhang et al., 2016)

No.	Derivatives and Constituents	Molecular Formula	PubChem CID/SID	Molecule Weight (g/mol)	References
KB029	13,14-Dehydromatrine	$C_{15}H_{22}N_2O$	NR #	246.35	(Y. B. Zhang et al., 2016)
KB030	Flavesine A	$C_{30}H_{48}N_4O_2$	NR #	496.74	(Y. B. Zhang et al., 2016)
KB031	Flavesine B	$C_{30}H_{48}N_4O_2$	NR #	496.74	(Y. B. Zhang et al., 2016)
KB032	Flavesine C	$C_{30}H_{46}N_4O_2$	NR #	494.72	(Y. B. Zhang et al., 2016)
KB033	Flavesine D	$C_{30}H_{44}N_4O_2$	NR #	492.71	(Y. B. Zhang et al., 2016)
KB034	Flavesine E	$C_{30}H_{46}N_4O_2$	NR #	494.72	(Y. B. Zhang et al., 2016)
KB035	Flavesine F	$C_{26}H_{36}N_4O_2$	NR #	436.6	(Y. B. Zhang et al., 2016)
KB036	Sophoridine	$C_{15}H_{24}N_2O$	165549	248.36	(Ji et al., 2017; Sun et al., 2017; R. Zhang et al., 2016; Zhang et al., 2015; D. Zhao et al., 2018; Zhao, 2015)
KB037	(+)-Cytisine	$C_{11}H_{14}N_2O$	NR	190.25	(Li et al., 2019)
KB038	17β-Hydroxysophoridine	$C_{15}H_{24}N_2O_2$	NR #	264.36	(D. Zhao et al., 2018)
KB039	7,11-Dehydromatrine	$C_{15}H_{22}N_2O$	5316459	246.35	(Li et al., 2019)
KB040	(+)-Epilamprolobine N-oxide	$C_{15}H_{24}N_2O_3$	274291215	280.36	(Zhao, 2015)
KB041	Oxysophoridine	$C_{15}H_{24}N_2O_2$	7061140	264.36	(Li et al., 2019; Zhao, 2015)
KB042	Oxymamanine	$C_{15}H_{22}N_2O_3$	NR #	278.35	(Zhao, 2015)
KB043	(-)-Lehmannine N-oxide	$C_{15}H_{22}N_2O_2$	NR #	262.35	(Zhao, 2015)
KB044	(+)-5α,9α-Dihydroxymatrine	$C_{15}H_{24}N_2O_3$	50221581	280.36	(Zhao, 2015)
KB045	(+)-9β-Hydroxylamprolobine	$C_{15}H_{24}N_2O_3$	NR #	280.37	(Li et al., 2019; Zhao, 2015)
KB046	(+)-9β-Hydroxylamprolobine N-oxide	$C_{15}H_{25}N_2O_4$	NR #	296.37	(Zhao, 2015)
KB047	Mamanine	$C_{15}H_{22}N_2O_2$	323274	262.35	(Zhao, 2015)
KB048	(+)-Sophoranhol N-oxide	$C_{15}H_{24}N_2O_3$	NR	280.37	(Li et al., 2019; Zhao, 2015)
KB049	5α,9α-Isodihydroxymatrine	$C_{15}H_{24}N_2O_3$	NR	280.37	(Li et al., 2019)
KB050	Liriodenine	$C_{17}H_9NO_3$	10144	275.26	(Zhao, 2015)
**KC**	**Terpenoids (n=10)**				
KC001	Lupeol	$C_{30}H_{50}O$	259846	426.7	(He et al., 2015)
KC002	Lupenone	$C_{30}H_{48}O$	92158	424.7	(He et al., 2015)
KC003	Monogynol B	$C_{30}H_{50}O$	NR #	426.7	(He et al., 2015)
KC004	β-Amyrenol	$C_{30}H_{50}O$	73145	426.7	(He et al., 2015)
KC005	Soyasaponin I	$C_{48}H_{78}O_{18}$	122097	943.1	(He et al., 2015)

No.	Derivatives and Constituents	Molecular Formula	PubChem CID/SID	Molecule Weight (g/mol)	References
KC006	Sophoraflavoside I	$C_{59}H_{96}O_{27}$	274478437	1237.4	(He et al., 2015)
KC007	Sophoraflavoside II	$C_{48}H_{76}O_{20}$	274198996	973.1	(He et al., 2015)
KC008	Sophoraflavoside III	$C_{53}H_{84}O_{24}$	274198997	1105.2	(He et al., 2015)
KC009	Sophoraflavoside IV	$C_{59}H_{94}O_{29}$	135229963	1267.4	(He et al., 2015)
KC010	Sophopterocarpan A	$C_{16}H_{16}O_6$	355426221	304.29	(H. Zhu et al., 2017)
**KD**	**Lignans (n=3)**				
KD001	Citrusin A	$C_{26}H_{34}O_{12}$	131752579	538.5	(He et al., 2015)
KD002	Citrusin B	$C_{27}H_{36}O_{13}$	131752580	568.6	(He et al., 2015)
KD003	Alaschanioside A	NR	NR	NR	(He et al., 2015)
**KE**	**Dibenzoyl derivatives (n=12)**				
KE001	Sophodibenzoside A	$C_{26}H_{30}O_{15}$	275217506	582.5	(He et al., 2015)
KE002	Sophodibenzoside B	$C_{26}H_{30}O_{15}$	275217507	582.5	(He et al., 2015)
KE003	Sophodibenzoside C	$C_{21}H_{22}O_{11}$	NR #	450.4	(He et al., 2015)
KE004	Sophodibenzoside D	$C_{26}H_{30}O_{14}$	NR #	566.51	(He et al., 2015)
KE005	Sophodibenzoside E	$C_{26}H_{30}O_{14}$	NR #	566.51	(He et al., 2015)
KE006	Sophodibenzoside F	$C_{21}H_{22}O_{10}$	NR #	434.4	(He et al., 2015)
KE007	Sophodibenzoside G	$C_{26}H_{28}O_{15}$	NR #	580.5	(He et al., 2015)
KE008	Sophodibenzoside H	$C_{26}H_{28}O_{15}$	NR #	580.5	(He et al., 2015)
KE009	Sophodibenzoside I	$C_{21}H_{20}O_{11}$	NR #	448.38	(He et al., 2015)
KE010	Sophodibenzoside J	$C_{26}H_{30}O_{15}$	NR #	582.51	(He et al., 2015)
KE011	Sophodibenzoside K	$C_{21}H_{22}O_{11}$	NR #	450.4	(He et al., 2015)
KE012	Sophodibenzoside L	$C_{25}H_{28}O_{15}$	NR #	568.48	(He et al., 2015)
**KF**	**Phenolic acids (n=7)**				
KF001	2,4-Dihydroxy benzoic acid	$C_7H_6O_4$	1491	154.12	(He et al., 2015)
KF002	T-cinnamic acid	$C_9H_8O_2$	444539	148.16	(Lee et al., 2018)
KF003	Benzoic acid	$C_7H_6O_2$	243	122.12	(Lee et al., 2018)
KF004	P-coumaric acid	$C_9H_8O_3$	637542	164.16	(Lee et al., 2018)
KF005	Caffeic acid	$C_9H_8O_4$	689043	180.16	(Lee et al., 2018)
KF006	Ferulic acid	$C_{10}H_{10}O_4$	445858	194.18	(Lee et al., 2018)
KF007	Chlorogenic acid	$C_{16}H_{18}O_9$	1794427	354.31	(Lee et al., 2018)

No.	Derivatives and Constituents	Molecular Formula	PubChem CID/SID	Molecule Weight (g/mol)	References
**KG**	**Saponins (n=1)**				
KG001	Kaikasaponin II	$C_{48}H_{78}O_{17}$	273966286	927.1	(Zhao, 2015)
**KH**	**Fatty acids (n=1)**				
KH001	Tetracosanoic acid	$C_{24}H_{48}O_2$	11197	368.6	(He et al., 2015)
KI	Coumarins (n=1)				
KI001	Umbelliferone	$C_9H_6O_3$	5281426	162.14	(He et al., 2015)
**KJ**	**Quinones (n=1)**				
KJ001	Kushequinone A	$C_{17}H_{22}O_4$	NR #	290.36	(He et al., 2015)
**KK**	**Sterols (n=1)**				
KK001	β-Sitosterol	$C_{29}H_{50}O$	222284	414.7	(He et al., 2015)
**KL**	**Polysaccharides (n=7)**				
KL001	SF1	NR	NR	NR	(Q. Zhang, J. Yu, L. Zhang, et al., 2016)
KL002	SF2	NR	NR	NR	(Q. Zhang, J. Yu, L. Zhang, et al., 2016)
KL003	SF3	NR	NR	NR	(Q. Zhang, J. Yu, L. Zhang, et al., 2016)
KL004	SF4	NR	NR	NR	(Q. Zhang, J. Yu, L. Zhang, et al., 2016)
KL005	SFP-100-A	NR	NR	NR	(Chen et al., 2018)
KL006	SFP-100-B	NR	NR	NR	(Chen et al., 2018)
KL007	SFP-100-C	NR	NR	NR	(Chen et al., 2018)
**KM**	**Elements (n=4)**				
KM001	Cadmium	Cd	23973	112.41	(Liu et al., 2018)
KM002	Copper	Cu	23978	63.55	(Liu et al., 2018)
KM003	Lead	Pb	5352425	207.2	(Liu et al., 2018)
KM004	Zinc	Zn	23994	65.38	(Liu et al., 2018)

Note: NR: Not reported; Corresponding molecular structures refer to PubChem and Supplementary Figs. S1 to S3 (#) This table was retrieved from our published thesis. Li, H. (2020). *Clinical applications and mechanisms of action of Danggui Beimu Kushen Wan on the targets and pathways implicated in prostate cancer: Literature reviews and computational analyses* [Doctoral dissertation, RMIT University]. RMIT Research Repository. Identification data of each compound, such as LC-MS, HPLC and UPLC-CAD, could refer to corresponding references.

Supplementary Table S2. Details of identified anti-cancer drugs from 2019 National Comprehensive Cancer Network Clinical Practice Guideline-Prostate Cancer and their drug targets.

Treatments and Drug Names	Drug Targets
**1. Androgen deprivation therapy**	
Goserelin	LHCGR, GNRHR
Histrelin	GNRHR
Leuprolide	GNRHR
Triptorelin	GNRHR
Nilutamide	AR
Flutamide	AR, AHR, NR1I2
Bicalutamide	AR
Degarelix	GNRHR
Abiraterone	CYP17A1
Apalutamide	AR
Enzalutamide	AR
Ketoconazole	AR, CYP17A1, CYP21A2, CYP19A1, KCHN2, NR1I2, NR1I3
**2. Chemotherapy**	
Docetaxel	TUBB1, BCL2, MAP2, MAP4, MAPT, NR1I2
Cabazitaxel	TUBA4A, TUBB1
**3. Immunotherapy**	
Sipuleucel-T	ACPP
Pembrolizumab	PDCD1
**4. Bone health**	
Denosumab	TNFSF11
Zoledronic acid	FDPS, GGPS1

Note: ACPP: Acid phosphatase prostate; AHR: Aryl hydrocarbon receptor; AR: Androgen receptor; BCL2: B-cell lymphoma-2; CYP17A1: Cytochrome P450 family 17 subfamily A member 1; CYP21A2: Cytochrome P450 family 21 subfamily A member 2; CYP19A1: Cytochrome P450 family 19 subfamily A member 1; FDPS: Farnesyl diphosphate synthase; GGPS1: Geranylgeranyl diphosphate synthase 1; GNRHR: Gonadotropin releasing hormone receptor; KCNH2: Potassium voltage-gated channel subfamily H member 2; LHCGR: Luteinising hormone/choriogonadotropin receptor; MAP2: Microtubule associated protein 2; MAP4: Microtubule associated protein 4; MAPT: Microtubule associated protein tau; NR1I2: Nuclear receptor subfamily 1 group I member 2; NR1I3: Nuclear receptor subfamily 1 group I member 3; PDCD1: Programmed cell death 1; TNFSF11: Tumour necrosis factor superfamily member 11; TUBA4A: Tubulin alpha 4a; TUBB1: Tubulin beta 1 class VI. Targets were identified from the 2019 National Comprehensive Cancer Network Clinical Practice Guideline-Prostate Cancer (National Comprehensive Cancer Network, 2019).

Supplementary Table S3. Summary of Kyoto Encyclopedia of Genes and Genomes enrichment of the included targets.

KEGG Term	p-value	Genes	Category of KEGG Term
Small cell lung cancer	1.08e-09	CASP3; PTEN; BCL2; BAX; PTGS2; TP53	6.2 Cancer: specific types
Pathways in cancer	5.52e-09	AR; CASP3; PTEN; BCL2; BAX; PTGS2; HIF1A; TP53; IL2	6.1 Cancer: overview
Measles	1.18e-08	CASP3; IL1B; BCL2; BAX; TP53; IL2	6.9 Infectious disease: viral
p53 signalling pathway	2.12e-08	CASP3; PTEN; BCL2; BAX; TP53	4.2 Cell growth and death
Kaposi sarcoma-associated herpesvirus infection	6.99e-08	CASP3; BAX; PTGS2; HIF1A; TP53; ICAM1	6.9 Infectious disease: viral
Nuclear factor-kappa signalling pathway	8.60e-08	IL1B; BCL2; TNFSF11; PTGS2; ICAM1	3.2 Signal transduction
AGE-RAGE signalling pathway in diabetic complications	1.11e-07	CASP3; IL1B; BCL2; BAX; ICAM1	6.7 Endocrine and metabolic disease
Human T-cell leukemia virus 1 infection	1.84e-07	FDPS; PTEN; BAX; TP53; IL2; ICAM1	6.9 Infectious disease: viral
Ovarian steroidogenesis	3.26e-07	LHCGR; PTGS2; CYP19A1; CYP17A1	5.2 Endocrine system
Amyotrophic lateral sclerosis	3.84e-07	CASP3; BCL2; BAX; TP53	6.4 Neurodegenerative disease
Apoptosis	6.64e-07	CASP3; BCL2; BAX; TP53; TUBA4A	4.2 Cell growth and death
Colorectal cancer	3.17e-06	CASP3; BCL2; BAX; TP53	6.2 Cancer: specific types
Epstein–Barr virus infection	3.55e-06	CASP3; BCL2; BAX; TP53; ICAM1	6.9 Infectious disease: viral
Prostate cancer	5.13e-06	AR; PTEN; BCL2; TP53	6.2 Cancer: specific types
Human cytomegalovirus infection	6.15e-06	CASP3; IL1B; BAX; PTGS2; TP53	6.9 Infectious disease: viral
Th17 cell differentiation	7.58e-06	IL1B; AHR; HIF1A; IL2	5.1 Immune system
TNF signalling pathway	8.45e-06	CASP3; IL1B; PTGS2; ICAM1	3.2 Signal transduction
Sphingolipid signalling pathway	1.15e-05	PTEN; BCL2; BAX; TP53	3.2 Signal transduction
Fluid shear stress and atherosclerosis	2.13e-05	IL1B; BCL2; TP53; ICAM1	6.6 Cardiovascular disease
MicroRNAs in cancer	2.43e-05	CASP3; PTEN; BCL2; PTGS2; TP53	6.1 Cancer: overview
Breast cancer	2.65e-05	PTEN; BAX; TNFSF11; TP53	6.2 Cancer: specific types

KEGG Term	p-value	Genes	Category of KEGG Term
Human papillomavirus infection	3.90e-05	CASP3; PTEN; BAX; PTGS2; TP53	6.9 Infectious disease: viral
Hepatitis B	3.98e-05	CASP3; BCL2; BAX; TP53	6.9 Infectious disease: viral
Endometrial cancer	4.49e-05	PTEN; BAX; TP53	6.2 Cancer: specific types
Steroid hormone biosynthesis	4.97e-05	CYP21A2; CYP19A1; CYP17A1	1.3 Lipid metabolism
Tuberculosis	5.73e-05	CASP3; IL1B; BCL2; BAX	6.8 Infectious disease: bacterial
Central carbon metabolism in cancer	6.32e-05	PTEN; HIF1A; TP53	6.1 Cancer: overview
Prolactin signalling pathway	7.88e-05	LHCGR; TNFSF11; CYP17A1	5.2 Endocrine system
Melanoma	8.58e-05	PTEN; BAX; TP53	6.2 Cancer: specific types
Glioma	9.69e-05	PTEN; BAX; TP53	6.2 Cancer: specific types
Rheumatoid arthritis	1.72e-04	IL1B; TNFSF11; ICAM1	6.3 Immune disease
IL-17 signalling pathway	1.84e-04	CASP3; IL1B; PTGS2	5.1 Immune system
Herpes simplex virus 1 infection	2.55e-04	CASP3; IL1B; BCL2; BAX; TP53	6.9 Infectious disease: viral
C-type lectin receptor signalling pathway	2.55e-04	IL1B; PTGS2; IL2	5.1 Immune system
Terpenoid backbone biosynthesis	3.14e-04	FDPS; GGPS1	1.9 Metabolism of terpenoids and polyketides
Neurotrophin signalling pathway	3.79e-04	BCL2; BAX; TP53	5.6 Nervous system
Autophagy	4.70e-04	PTEN; BCL2; HIF1A	4.1 Transport and catabolism
Non-alcoholic fatty liver disease	7.31e-04	CASP3; IL1B; BAX	6.7 Endocrine and metabolic disease
Gastric cancer	7.31e-04	BCL2; BAX; TP53	6.2 Cancer: specific types
PI3K-Akt signalling pathway	7.75e-04	PTEN; BCL2; TP53; IL2	3.2 Signal transduction
Prion diseases	8.01e-04	IL1B; BAX	6.4 Neurodegenerative disease
Hepatitis C	8.20e-04	CASP3; BAX; TP53	6.9 Infectious disease: viral
Cushing syndrome	8.20e-04	CYP21A2; AHR; CYP17A1	6.7 Endocrine and metabolic disease
Thyroid cancer	8.96e-04	BAX; TP53	6.2 Cancer: specific types
African trypanosomiasis	8.96e-04	IL1B; ICAM1	6.10 Infectious disease: parasitic
Necroptosis	9.32e-04	IL1B; BCL2; BAX	4.2 Cell growth and death
Hepatocellular carcinoma	1.03e-03	PTEN; BAX; TP53	6.2 Cancer: specific types
Influenza A	1.09e-03	FDPS; IL1B; ICAM1	6.9 Infectious disease: viral
Graft-versus-host disease	1.10e-03	IL1B; IL2	6.3 Immune disease
Type I diabetes mellitus	1.21e-03	IL1B; IL2	6.7 Endocrine and metabolic disease
Huntington disease	1.54e-03	CASP3; BAX; TP53	6.4 Neurodegenerative disease
Malaria	1.57e-03	IL1B; ICAM1	6.10 Infectious disease: parasitic
Proteoglycans in cancer	1.73e-03	CASP3; HIF1A; TP53	6.1 Cancer: overview
Viral carcinogenesis	1.73e-03	CASP3; BAX; TP53	6.1 Cancer: overview

KEGG Term	p-value	Genes	Category of KEGG Term
Pathogenic Escherichia coli infection	1.97e-03	TUBB1; TUBA4A	6.8 Infectious disease: bacterial
Legionellosis	1.97e-03	CASP3; IL1B	6.8 Infectious disease: bacterial
Human immunodeficiency virus 1 infection	2.02e-03	CASP3; BCL2; BAX	6.9 Infectious disease: viral
Viral myocarditis	2.26e-03	CASP3; ICAM1	6.6 Cardiovascular disease
Basal cell carcinoma	2.58e-03	BAX; TP53	6.2 Cancer: specific types
Mitophagy	2.74e-03	HIF1A; TP53	4.1 Transport and catabolism
Inflammatory bowel disease	2.74e-03	IL1B; IL2	6.3 Immune disease
Cortisol synthesis and secretion	2.74e-03	CYP21A2; CYP17A1	5.2 Endocrine system
Non-small cell lung cancer	2.82e-03	BAX; TP53	6.2 Cancer: specific types
Leishmaniasis	3.54e-03	IL1B; PTGS2	6.10 Infectious disease: parasitic
Pancreatic cancer	3.63e-03	BAX; TP53	6.2 Cancer: specific types
Pertussis	3.73e-03	CASP3; IL1B	6.8 Infectious disease: bacterial
Chronic myeloid leukemia	3.73e-03	BAX; TP53	6.2 Cancer: specific types
Gap junction	4.96e-03	TUBB1; TUBA4A	4.3 Cellular community - eukaryotes
Cytokine-cytokine receptor interaction	5.06e-03	IL1B; TNFSF11; IL2	3.3 Signalling molecules and interaction
MAPK signalling pathway	5.11e-03	CASP3; IL1B; TP53	3.2 Signal transduction
Amoebiasis	5.87e-03	CASP3; IL1B	6.10 Infectious disease: parasitic
HIF-1 signalling pathway	6.36e-03	BCL2; HIF1A	3.2 Signal transduction
T cell receptor signalling pathway	6.48e-03	PDCD1; IL2	5.1 Immune system
Longevity regulating pathway	6.61e-03	BAX; TP53	5.9 Aging
Chagas disease (American trypanosomiasis)	6.73e-03	IL1B; IL2	6.10 Infectious disease: parasitic
Parathyroid hormone synthesis, secretion and action	7.12e-03	BCL2; TNFSF11	5.2 Endocrine system
Serotonergic synapse	8.05e-03	CASP3; PTGS2	5.6 Nervous system
Toxoplasmosis	8.05e-03	CASP3; BCL2	6.10 Infectious disease: parasitic
Thyroid hormone signalling pathway	8.47e-03	HIF1A; TP53	5.2 Endocrine system
Osteoclast differentiation	1.01e-02	IL1B; TNFSF11	5.8 Development and regeneration
Natural killer cell mediated cytotoxicity	1.07e-02	CASP3; ICAM1	5.1 Immune system
Cell adhesion molecules	1.30e-02	PDCD1; ICAM1	3.3 Signalling molecules and interaction
Phagosome	1.42e-02	TUBB1; TUBA4A	4.1 Transport and catabolism
Cellular senescence	1.56e-02	PTEN; TP53	4.2 Cell growth and death
JAK-STAT signalling pathway	1.60e-02	BCL2; IL2	3.2 Signal transduction
Protein processing in endoplasmic reticulum	1.66e-02	BCL2; BAX	2.3 Folding, sorting and degradation

KEGG Term	p-value	Genes	Category of KEGG Term
Alzheimer disease	1.77e-02	CASP3; IL1B	6.4 Neurodegenerative disease
NOD-like receptor signalling pathway	1.91e-02	IL1B; BCL2	5.1 Immune system
Transcriptional misregulation in cancer	2.08e-02	BAX; TP53	6.1 Cancer: overview
Focal adhesion	2.35e-02	PTEN; BCL2	4.3 Cellular community - eukaryotes
Allograft rejection	4.46e-02	IL2	6.3 Immune disease
Ferroptosis	4.69e-02	TP53	4.2 Cell growth and death
Bladder cancer	4.81e-02	TP53	6.2 Cancer: specific types

Note: AR: Androgen receptor; BAX: B-cell lymphoma-2 associated X; BCL2: B-cell lymphoma-2; CASP3: Caspase 3; CYP17A1: Cytochrome P450 family 17 subfamily A member 1; CYP21A2: Cytochrome P450 family 21 subfamily A member 2; CYP19A1: Cytochrome P450 family 19 subfamily A member 1; FDPS: Farnesyl diphosphate synthase; GGPS1: Geranylgeranyl diphosphate synthase1; GNRHR: Gonadotropin releasing hormone receptor; HIF1A: Hypoxia inducible factor-1α; ICAM1: Intercellular cell adhesion molecule 1; IL1B: Interleukin 1β; IL2: Interleukin 2; LHCGR: Luteinizing hormone/choriogonadotropin receptor; MAP4: Microtubule associated protein 4; MAPT: Microtubule associated protein tau; NR1I2: Nuclear receptor subfamily 1 group I member 2; PDCD1: Programmed cell death 1; PTEN: Phosphatase and tensin homolog; PTGS2: Prostaglandin-endoperoxide synthase 2; TNFSF11: Tumour necrosis factor superfamily member 11; TP53: Tumour protein 53; TUBA4A: Tubulin alpha 4a; TUBB1: Tubulin beta 1 class VI.

Supplementary Table S4. Details of docking results between 621 natural compounds from Danggui Beimu Kushen Wan and 21 targets for prostate cancer (kcal/mol).

Compound ID	Min	25% Percentile	Med	75% Percentile	Max	Average Value	SD	SEM	95% Confidence Interval of Mean	
									Lower	Upper
DA001	−8.3	−7.9	−7.3	−6.4	−5.9	−7.17	0.79	0.17	−7.53	−6.81
DA002	−7.7	−6.8	−6.6	−6.0	−5.4	−6.47	0.64	0.14	−6.76	−6.18
DA003	−6.5	−6.1	−5.7	−5.5	−4.8	−5.76	0.49	0.11	−5.98	−5.53
DA004	−4.1	−3.7	−3.6	−3.3	−2.8	−3.53	0.34	0.08	−3.69	−3.37
DA005	−6.5	−6.1	−5.6	−5.1	−4.5	−5.61	0.60	0.13	−5.88	−5.34
DA006	−7.2	−5.9	−5.5	−5.1	−3.9	−5.49	0.92	0.20	−5.90	−5.07
DA007	−9.7	−7.3	−6.6	−5.7	−5.1	−6.61	1.13	0.25	−7.13	−6.10
DA008	−7.1	−6.4	−6.1	−5.6	−4.5	−5.93	0.73	0.16	−6.26	−5.59
DA009	−6.7	−5.3	−4.9	−4.2	−3.3	−4.87	0.82	0.18	−5.24	−4.49
DA010	−5.6	−5.2	−4.9	−4.9	−4.2	−5.00	0.35	0.08	−5.16	−4.84
DA011	−3.9	−3.8	−3.6	−3.3	−2.9	−3.50	0.32	0.07	−3.65	−3.36
DA012	−7.7	−7.0	−6.0	−5.9	−4.9	−6.33	0.80	0.17	−6.70	−5.97
DA013	−6.5	−5.8	−5.5	−5.2	−4.6	−5.47	0.48	0.11	−5.69	−5.25
DA014	−4.4	−4.2	−3.9	−3.7	−3.1	−3.90	0.35	0.08	−4.06	−3.75
DA015	−4.6	−4.1	−4.0	−3.8	−3.2	−3.92	0.33	0.07	−4.07	−3.77
DA016	−5.9	−5.5	−5.2	−4.7	−4.4	−5.16	0.47	0.10	−5.37	−4.94
DA017	−6.1	−5.7	−5.2	−5.0	−4.1	−5.18	0.59	0.13	−5.45	−4.91
DA018	−5.9	−5.2	−4.7	−4.2	−3.3	−4.65	0.74	0.16	−4.98	−4.31
DA019	−5.3	−5.0	−4.6	−4.4	−3.3	−4.59	0.55	0.12	−4.84	−4.34
DA020	−6.2	−5.5	−5.2	−4.7	−3.8	−5.11	0.60	0.13	−5.38	−4.83
DA021	−10.4	−8.7	−8.2	−7.2	−6.2	−8.16	1.13	0.25	−8.68	−7.65
DA022	−9.0	−7.7	−7.2	−6.8	−5.5	−7.16	0.85	0.19	−7.55	−6.77
DA023	−7.1	−5.7	−5.3	−4.9	−4.3	−5.36	0.65	0.14	−5.65	−5.06
DA024	−5.4	−5.0	−4.7	−4.3	−3.8	−4.62	0.49	0.11	−4.84	−4.40
DA025	−6.7	−6.1	−5.6	−5.3	−4.3	−5.60	0.71	0.15	−5.92	−5.27
DA026	−6.7	−6.2	−5.8	−5.4	−4.5	−5.71	0.66	0.14	−6.02	−5.41
DA027	−9.7	−9.0	−8.3	−7.3	−6.2	−8.14	1.04	0.23	−8.62	−7.67

Compound ID	Min	25% Percentile	Med	75% Percentile	Max	Average Value	SD	SEM	95% Confidence Interval of Mean	
									Lower	Upper
DA028	−8.5	−7.4	−6.9	−6.3	−5.5	−6.92	0.74	0.16	−7.26	−6.58
DA029	−10.5	−9.1	−8.2	−7.8	−6.5	−8.40	1.03	0.22	−8.86	−7.93
DA030	−5.2	−4.6	−4.3	−4.0	−3.4	−4.28	0.52	0.11	−4.52	−4.04
DA031	−9.1	−7.3	−7.0	−6.5	−5.4	−6.88	0.91	0.20	−7.29	−6.46
DA032	−5.5	−4.9	−4.7	−4.4	−3.7	−4.70	0.50	0.11	−4.92	−4.47
DA033	−9.3	−7.7	−6.7	−6.3	−5.1	−6.90	1.09	0.24	−7.40	−6.40
DA035	−9.5	−7.5	−7.0	−5.8	−5.1	−6.80	1.17	0.26	−7.33	−6.27
DA036	−8.7	−7.2	−6.7	−5.6	−5.1	−6.61	1.00	0.22	−7.06	−6.15
DA037	−9.4	−7.5	−6.8	−5.9	−5.5	−6.92	1.01	0.22	−7.39	−6.46
DA038	−9.5	−7.3	−6.9	−6.0	−5.2	−6.85	1.00	0.22	−7.30	−6.39
DA039	−7.3	−5.9	−5.6	−5.3	−4.0	−5.59	0.83	0.18	−5.97	−5.21
DA040	−9.8	−8.4	−7.8	−6.8	−5.8	−7.75	1.07	0.23	−8.24	−7.27
DA041	−6.0	−5.5	−5.1	−4.9	−3.6	−5.12	0.66	0.14	−5.42	−4.83
DA042	−7.7	−6.6	−6.3	−5.9	−5.1	−6.31	0.63	0.14	−6.60	−6.03
DA043	−8.6	−7.8	−7.2	−6.3	−5.8	−7.04	0.90	0.20	−7.45	−6.63
DA044	−7.1	−5.7	−5.3	−4.7	−3.9	−5.31	0.82	0.18	−5.69	−4.94
DA046	−6.1	−5.7	−5.5	−5.0	−4.5	−5.40	0.47	0.10	−5.61	−5.19
DA047	−9.2	−7.3	−6.8	−6.0	−5.1	−6.73	1.03	0.23	−7.20	−6.26
DA050	−9.5	−7.2	−6.8	−5.6	−5.3	−6.70	1.11	0.24	−7.20	−6.20
DA051	−7.2	−6.4	−5.7	−5.1	−4.5	−5.75	0.81	0.18	−6.12	−5.38
DA052	−7.0	−6.4	−5.9	−5.5	−4.7	−5.90	0.66	0.14	−6.20	−5.60
DA053	−6.6	−6.2	−5.6	−5.4	−4.5	−5.74	0.61	0.13	−6.02	−5.46
DA054	−10.0	−7.3	−6.7	−6.4	−5.3	−6.86	1.08	0.24	−7.35	−6.36
DA055	−9.1	−7.5	−6.7	−5.5	−5.1	−6.66	1.08	0.24	−7.15	−6.16
DA056	−9.3	−7.4	−6.9	−5.7	−5.2	−6.74	1.13	0.25	−7.26	−6.23
DA057	−9.6	−7.2	−6.7	−5.9	−5.1	−6.72	1.10	0.24	−7.22	−6.22
DA058	−7.0	−6.2	−5.7	−5.5	−4.4	−5.79	0.71	0.15	−6.11	−5.47
DA059	−6.5	−5.7	−5.5	−5.2	−4.1	−5.45	0.57	0.12	−5.71	−5.20
DA060	−8.9	−6.9	−6.8	−5.6	−4.9	−6.55	0.98	0.21	−7.00	−6.11
DA061	−8.1	−7.0	−6.7	−6.1	−5.1	−6.57	0.79	0.17	−6.93	−6.21
DA062	−7.7	−6.9	−6.2	−5.9	−4.7	−6.40	0.75	0.16	−6.74	−6.05

Compound ID	Min	25% Percentile	Med	75% Percentile	Max	Average Value	SD	SEM	95% Confidence Interval of Mean	
									Lower	Upper
DA063	−7.5	−6.6	−6.3	−5.7	−4.8	−6.20	0.71	0.15	−6.52	−5.87
DA064	−9.7	−7.7	−6.7	−6.2	−5.3	−6.94	1.18	0.26	−7.48	−6.40
DA065	−6.9	−6.1	−5.6	−5.1	−4.2	−5.58	0.75	0.16	−5.92	−5.24
DA066	−7.1	−6.1	−5.6	−5.4	−4.5	−5.66	0.69	0.15	−5.98	−5.35
DA067	−7.0	−5.8	−5.4	−5.1	−4.2	−5.50	0.81	0.18	−5.87	−5.13
DA068	−7.9	−6.5	−5.8	−5.4	−4.8	−6.04	0.88	0.19	−6.44	−5.64
DA070	−7.1	−6.3	−5.8	−5.2	−4.6	−5.81	0.72	0.16	−6.14	−5.48
DA071	−6.2	−5.6	−5.2	−4.8	−4.0	−5.18	0.63	0.14	−5.46	−4.89
DA072	−5.4	−4.9	−4.4	−4.2	−3.0	−4.42	0.62	0.14	−4.71	−4.14
DA073	−6.0	−5.4	−5.0	−4.6	−3.7	−4.96	0.66	0.14	−5.26	−4.66
DA074	−8.8	−6.8	−6.3	−5.7	−5.0	−6.37	0.95	0.21	−6.80	−5.94
DA075	−8.7	−6.4	−5.9	−5.1	−4.8	−6.00	1.04	0.23	−6.47	−5.53
DA076	−6.2	−5.3	−4.5	−4.0	−3.2	−4.59	0.83	0.18	−4.97	−4.21
DA077	−6.5	−5.7	−5.4	−5.0	−4.0	−5.29	0.70	0.15	−5.60	−4.97
DA078	−8.7	−7.5	−6.6	−5.9	−5.3	−6.78	0.97	0.21	−7.22	−6.34
DA079	−9.3	−7.1	−6.6	−5.7	−5.2	−6.60	1.06	0.23	−7.08	−6.12
DA080	−9.6	−7.7	−6.8	−6.0	−5.2	−6.92	1.17	0.25	−7.45	−6.39
DA081	−5.5	−5.1	−4.7	−4.4	−4.1	−4.74	0.44	0.09	−4.94	−4.54
DA082	−6.7	−6.2	−5.8	−5.4	−4.4	−5.74	0.72	0.16	−6.07	−5.41
DA083	−8.7	−7.2	−6.4	−5.7	−5.0	−6.50	1.07	0.23	−6.98	−6.01
DA084	−9.2	−7.3	−6.8	−5.5	−5.2	−6.69	1.16	0.25	−7.21	−6.16
DA085	−10.0	−7.4	−6.9	−5.7	−5.1	−6.75	1.19	0.26	−7.29	−6.21
DA087	−6.7	−6.1	−5.5	−4.9	−4.3	−5.51	0.71	0.15	−5.83	−5.19
DA088	−6.1	−5.5	−5.1	−4.9	−3.7	−5.05	0.60	0.13	−5.33	−4.78
DA089	−7.2	−5.8	−5.6	−5.1	−4.0	−5.55	0.84	0.18	−5.93	−5.17
DA090	−6.6	−6.0	−5.7	−5.3	−4.1	−5.56	0.69	0.15	−5.88	−5.25
DA091	−6.8	−6.2	−6.0	−5.3	−4.5	−5.77	0.70	0.15	−6.09	−5.45
DA092	−6.2	−5.1	−4.5	−4.0	−3.3	−4.57	0.81	0.18	−4.94	−4.20
DA093	−7.9	−6.8	−6.3	−5.8	−5.0	−6.38	0.84	0.18	−6.76	−6.00
DA095	−6.1	−5.6	−5.3	−5.0	−4.0	−5.25	0.53	0.12	−5.49	−5.01
DA096	−5.9	−5.3	−5.1	−4.8	−3.8	−5.00	0.58	0.13	−5.27	−4.73

Compound ID	Min	25% Percentile	Med	75% Percentile	Max	Average Value	SD	SEM	95% Confidence Interval of Mean	
									Lower	Upper
DA097	−6.7	−6.1	−5.5	−5.2	−4.0	−5.57	0.78	0.17	−5.93	−5.22
DA098	−6.1	−5.6	−5.4	−5.0	−4.2	−5.26	0.55	0.12	−5.51	−5.01
DA099	−8.2	−7.0	−6.0	−5.5	−3.5	−6.06	1.17	0.26	−6.60	−5.53
DA100	−6.1	−5.6	−5.4	−5.1	−4.0	−5.26	0.56	0.12	−5.52	−5.01
DA101	−5.7	−5.0	−4.8	−4.6	−3.4	−4.67	0.55	0.12	−4.92	−4.42
DA102	−4.6	−4.3	−4.0	−4.0	−3.3	−4.05	0.32	0.07	−4.20	−3.91
DA103	−6.5	−5.3	−4.7	−4.4	−4.0	−4.90	0.68	0.15	−5.21	−4.59
DA104	−6.2	−5.4	−4.9	−4.7	−3.9	−5.02	0.58	0.13	−5.29	−4.76
DA105	−6.6	−6.5	−6.0	−5.5	−4.9	−5.91	0.58	0.13	−6.17	−5.65
DA106	−6.6	−5.5	−4.8	−4.5	−3.8	−5.05	0.75	0.16	−5.39	−4.71
DA107	−6.5	−5.3	−5.1	−4.6	−3.9	−5.10	0.76	0.17	−5.45	−4.76
DA108	−6.8	−5.6	−4.9	−4.6	−4.1	−5.10	0.70	0.15	−5.42	−4.79
DA109	−6.6	−6.1	−5.6	−5.2	−4.1	−5.61	0.62	0.13	−5.89	−5.33
DA110	−6.7	−6.0	−5.8	−5.3	−4.6	−5.64	0.60	0.13	−5.92	−5.37
DA111	−7.1	−6.4	−6.0	−5.5	−4.7	−5.85	0.68	0.15	−6.16	−5.54
DA112	−6.7	−6.0	−5.8	−5.4	−4.8	−5.76	0.59	0.13	−6.03	−5.49
DA114	−5.9	−5.0	−4.5	−4.3	−3.4	−4.58	0.62	0.14	−4.86	−4.30
DA115	−6.4	−6.0	−5.7	−5.1	−4.6	−5.56	0.52	0.11	−5.80	−5.32
DA116	−6.1	−5.0	−4.7	−4.1	−3.2	−4.65	0.73	0.16	−4.98	−4.32
DA117	−5.7	−5.2	−4.9	−4.3	−3.8	−4.80	0.55	0.12	−5.05	−4.55
DA118	−6.3	−5.3	−4.9	−4.6	−3.7	−4.89	0.67	0.15	−5.20	−4.58
DA119	−6.2	−5.8	−5.6	−5.2	−4.6	−5.45	0.48	0.11	−5.67	−5.23
DA120	−6.1	−5.4	−4.8	−4.3	−3.6	−4.79	0.71	0.16	−5.11	−4.47
DA121	−5.7	−5.2	−4.5	−4.2	−3.2	−4.60	0.73	0.16	−4.93	−4.27
DA122	−5.5	−5.0	−4.7	−4.3	−3.1	−4.56	0.64	0.14	−4.85	−4.27
DA123	−6.2	−6.0	−5.6	−4.9	−4.3	−5.50	0.59	0.13	−5.77	−5.24
DA125	−6.8	−6.1	−5.7	−5.1	−4.3	−5.64	0.68	0.15	−5.95	−5.33
DA126	−7.6	−6.8	−6.3	−5.6	−4.8	−6.26	0.83	0.18	−6.64	−5.88
DA127	−5.7	−5.4	−5.1	−4.5	−3.7	−4.93	0.61	0.13	−5.21	−4.66
DA128	−7.3	−6.5	−5.7	−5.6	−4.6	−5.87	0.78	0.17	−6.22	−5.51
DA129	−7.8	−6.2	−5.8	−4.9	−4.6	−5.83	0.96	0.21	−6.26	−5.39

Compound ID	Min	25% Percentile	Med	75% Percentile	Max	Average Value	SD	SEM	95% Confidence Interval of Mean	
									Lower	Upper
DA130	−5.6	−5.1	−4.8	−4.6	−3.8	−4.77	0.47	0.10	−4.98	−4.55
DA133	−6.3	−5.4	−4.8	−4.4	−3.7	−4.88	0.66	0.14	−5.18	−4.57
DA134	−6.1	−5.3	−5.1	−4.8	−4.0	−5.03	0.51	0.11	−5.27	−4.80
DA136	−6.7	−5.7	−5.4	−4.9	−3.7	−5.30	0.75	0.16	−5.64	−4.96
DA137	−9.3	−7.0	−6.3	−5.4	−4.8	−6.37	1.15	0.25	−6.90	−5.85
DA138	−10.7	−8.9	−8.0	−7.5	−6.4	−8.19	1.09	0.24	−8.68	−7.69
DA139	−6.8	−6.4	−5.7	−5.4	−4.8	−5.81	0.62	0.14	−6.09	−5.53
DA140	−7.0	−6.2	−6.0	−5.6	−4.7	−5.84	0.66	0.14	−6.14	−5.54
DA141	−5.9	−5.5	−5.2	−4.6	−3.6	−5.03	0.66	0.14	−5.33	−4.73
DA143	−5.5	−5.0	−4.7	−4.2	−3.7	−4.62	0.56	0.12	−4.88	−4.37
DA144	−5.8	−5.1	−4.8	−4.1	−3.6	−4.67	0.70	0.15	−4.99	−4.35
DA145	−6.1	−5.4	−5.0	−4.5	−3.7	−5.01	0.62	0.14	−5.29	−4.73
DA146	−6.9	−5.4	−4.8	−4.4	−3.8	−4.92	0.77	0.17	−5.27	−4.58
DA147	−6.0	−5.3	−4.8	−4.4	−3.4	−4.77	0.76	0.17	−5.12	−4.42
DA148	−8.5	−7.1	−6.8	−5.6	−5.2	−6.60	1.05	0.23	−7.08	−6.12
DA149	−4.8	−4.3	−4.1	−3.9	−3.2	−4.09	0.40	0.09	−4.27	−3.91
DA150	−6.0	−5.2	−4.7	−4.4	−3.3	−4.70	0.69	0.15	−5.01	−4.38
DA152	−6.7	−5.6	−5.2	−4.7	−4.1	−5.22	0.66	0.14	−5.52	−4.92
DA153	−7.3	−6.1	−5.2	−5.0	−3.9	−5.43	0.98	0.21	−5.87	−4.98
DA154	−6.3	−5.4	−5.1	−4.3	−3.6	−4.90	0.75	0.16	−5.24	−4.56
DA155	−4.7	−4.4	−4.2	−4.0	−3.4	−4.15	0.35	0.08	−4.31	−3.99
DA156	−5.5	−4.8	−4.3	−4.1	−3.5	−4.37	0.51	0.11	−4.60	−4.13
DA157	−5.2	−4.8	−4.5	−4.0	−3.3	−4.38	0.55	0.12	−4.63	−4.12
DA158	−5.3	−4.7	−4.2	−4.1	−3.0	−4.27	0.58	0.13	−4.53	−4.00
DA159	−8.4	−6.9	−6.4	−5.6	−4.8	−6.40	0.95	0.21	−6.83	−5.97
DA160	−8.4	−7.2	−6.7	−6.1	−5.3	−6.78	0.88	0.19	−7.18	−6.38
DA161	−5.8	−5.3	−4.9	−4.3	−3.7	−4.77	0.62	0.13	−5.05	−4.49
DA162	−11.0	−8.6	−8.0	−7.3	−6.0	−8.10	1.25	0.27	−8.67	−7.52
DA163	−6.1	−5.0	−4.6	−4.1	−3.4	−4.60	0.75	0.16	−4.95	−4.26
DA164	−5.9	−5.1	−4.9	−4.5	−4.0	−4.87	0.48	0.10	−5.09	−4.65
DA165	−6.1	−5.2	−4.9	−4.2	−3.3	−4.76	0.71	0.15	−5.08	−4.44

Compound ID	Min	25% Percentile	Med	75% Percentile	Max	Average Value	SD	SEM	95% Confidence Interval of Mean	
									Lower	Upper
DA166	−7.9	−7.0	−6.6	−5.7	−5.2	−6.44	0.79	0.17	−6.80	−6.08
DA168	−6.4	−5.7	−5.4	−5.0	−4.0	−5.33	0.61	0.13	−5.61	−5.05
DA169	−6.8	−6.2	−5.8	−5.3	−4.5	−5.72	0.73	0.16	−6.05	−5.39
DA170	−10.0	−7.9	−7.1	−6.7	−6.0	−7.45	1.04	0.23	−7.93	−6.98
DA171	−6.2	−5.6	−5.1	−4.9	−4.2	−5.12	0.54	0.12	−5.36	−4.87
DA172	−5.3	−4.7	−4.5	−4.2	−3.4	−4.43	0.54	0.12	−4.68	−4.19
DA173	−5.8	−5.0	−4.6	−4.4	−3.5	−4.63	0.58	0.13	−4.90	−4.37
DA174	−5.8	−5.2	−4.8	−4.1	−3.4	−4.67	0.74	0.16	−5.00	−4.33
DA175	−6.3	−5.7	−5.4	−5.0	−4.1	−5.32	0.54	0.12	−5.57	−5.07
DA176	−6.9	−5.8	−5.5	−4.7	−4.0	−5.38	0.86	0.19	−5.77	−4.99
DA177	−5.6	−5.0	−4.7	−4.5	−4.0	−4.76	0.40	0.09	−4.94	−4.58
DA178	−5.5	−5.2	−4.9	−4.6	−3.3	−4.78	0.63	0.14	−5.07	−4.49
DA179	−6.4	−5.5	−5.0	−4.8	−3.7	−5.06	0.74	0.16	−5.40	−4.72
DA180	−6.6	−5.7	−5.4	−4.8	−4.0	−5.31	0.70	0.15	−5.63	−4.99
DA182	−5.6	−4.9	−4.6	−4.2	−3.6	−4.60	0.55	0.12	−4.84	−4.35
DA183	−6.1	−5.6	−5.4	−5.2	−4.3	−5.34	0.48	0.10	−5.56	−5.12
DA184	−8.0	−6.7	−6.3	−5.6	−4.3	−6.22	0.89	0.19	−6.63	−5.82
DA185	−6.6	−5.7	−5.4	−5.0	−4.1	−5.30	0.59	0.13	−5.56	−5.03
DA186	−6.9	−6.2	−5.9	−5.5	−4.4	−5.75	0.68	0.15	−6.06	−5.44
DA188	−7.1	−5.9	−5.6	−5.2	−4.0	−5.57	0.80	0.18	−5.94	−5.21
DA190	−7.6	−6.3	−5.9	−5.1	−4.4	−5.87	0.92	0.20	−6.28	−5.45
DA191	−8.5	−7.2	−6.7	−6.1	−5.3	−6.70	0.83	0.18	−7.08	−6.33
DA192	−9.0	−7.2	−6.9	−6.3	−5.4	−6.83	0.88	0.19	−7.23	−6.43
DA193	−8.8	−6.7	−6.1	−5.5	−4.8	−6.20	0.99	0.22	−6.66	−5.75
DA196	−6.0	−5.1	−4.8	−4.2	−3.4	−4.65	0.66	0.14	−4.95	−4.35
DA198	−8.1	−6.8	−6.4	−5.6	−4.2	−6.19	0.92	0.20	−6.61	−5.77
DA199	−6.6	−5.6	−5.4	−4.8	−3.9	−5.28	0.70	0.15	−5.59	−4.96
DA200	−7.0	−6.3	−5.9	−5.5	−4.5	−5.82	0.68	0.15	−6.13	−5.51
DA201	−6.8	−6.1	−5.9	−4.9	−4.1	−5.59	0.75	0.16	−5.93	−5.24
DA202	−10.4	−8.7	−7.9	−7.1	−5.6	−8.05	1.24	0.27	−8.61	−7.48
DA203	−10.6	−9.0	−8.3	−7.5	−5.4	−8.20	1.29	0.28	−8.79	−7.61

Compound ID	Min	25% Percentile	Med	75% Percentile	Max	Average Value	SD	SEM	95% Confidence Interval of Mean	
									Lower	Upper
DA205	−8.9	−7.3	−6.7	−5.7	−5.0	−6.62	1.07	0.23	−7.11	−6.13
DA206	−5.8	−5.3	−5.1	−4.9	−4.1	−5.04	0.43	0.09	−5.24	−4.85
DA207	−10.6	−8.9	−8.3	−7.4	−6.2	−8.23	1.03	0.22	−8.70	−7.76
DA208	−8.2	−7.3	−6.5	−5.8	−5.1	−6.59	0.96	0.21	−7.02	−6.15
DA209	−6.8	−6.0	−5.7	−5.3	−4.6	−5.65	0.66	0.14	−5.95	−5.35
DA210	−5.3	−4.9	−4.5	−4.2	−3.0	−4.45	0.64	0.14	−4.74	−4.16
DA211	−8.3	−7.0	−6.5	−6.0	−5.0	−6.58	0.78	0.17	−6.94	−6.23
DA212	−5.0	−4.3	−4.1	−3.9	−3.1	−4.08	0.44	0.10	−4.28	−3.87
DA213	−6.7	−5.7	−5.4	−4.8	−4.3	−5.40	0.69	0.15	−5.72	−5.08
DA214	−6.1	−5.5	−5.3	−5.0	−4.5	−5.28	0.43	0.09	−5.47	−5.08
DA215	−5.9	−5.5	−5.1	−4.8	−4.2	−5.10	0.48	0.10	−5.31	−4.88
DA216	−6.3	−5.9	−5.5	−5.2	−4.5	−5.43	0.54	0.12	−5.68	−5.19
DB001	−7.8	−6.8	−6.2	−6.0	−5.0	−6.31	0.73	0.16	−6.65	−5.98
DB002	−7.7	−6.5	−6.1	−5.8	−5.1	−6.24	0.76	0.16	−6.59	−5.90
DB003	−7.8	−7.0	−6.4	−6.1	−5.1	−6.47	0.77	0.17	−6.82	−6.11
DB004	−7.8	−7.0	−6.6	−6.2	−5.1	−6.55	0.72	0.16	−6.88	−6.22
DB005	−7.9	−6.9	−6.3	−6.0	−4.9	−6.42	0.81	0.18	−6.79	−6.05
DB006	−7.6	−6.7	−6.2	−5.9	−5.1	−6.26	0.72	0.16	−6.59	−5.94
DB007	−7.0	−6.7	−6.4	−5.9	−5.0	−6.28	0.56	0.12	−6.53	−6.02
DB008	−9.9	−8.7	−8.0	−7.0	−6.2	−7.89	1.07	0.23	−8.38	−7.40
DB009	−7.8	−6.9	−6.5	−6.0	−5.0	−6.41	0.70	0.15	−6.73	−6.10
DB010	−9.3	−8.4	−7.9	−6.7	−5.9	−7.72	0.97	0.21	−8.16	−7.28
DB012	−9.5	−8.6	−8.4	−6.9	−6.1	−7.90	1.07	0.23	−8.39	−7.41
DB013	−9.6	−8.0	−7.5	−6.6	−5.6	−7.39	0.93	0.20	−7.82	−6.97
DB014	−7.4	−6.9	−6.4	−5.9	−4.8	−6.25	0.72	0.16	−6.57	−5.92
DB015	−10.3	−8.1	−7.5	−6.9	−5.7	−7.67	1.09	0.24	−8.16	−7.17
DB016	−9.9	−8.6	−7.9	−7.4	−5.9	−7.88	1.03	0.22	−8.34	−7.41
DB017	−9.4	−8.1	−7.8	−6.8	−6.0	−7.57	0.98	0.21	−8.02	−7.12
DB018	−11.1	−8.4	−7.5	−6.8	−6.0	−7.68	1.16	0.25	−8.21	−7.15
DB019	−7.7	−6.9	−6.5	−6.0	−5.0	−6.43	0.73	0.16	−6.76	−6.10
DB024	−7.7	−6.8	−6.2	−6.1	−5.1	−6.37	0.73	0.16	−6.70	−6.03

Compound ID	Min	25% Percentile	Med	75% Percentile	Max	Average Value	SD	SEM	95% Confidence Interval of Mean	
									Lower	Upper
DB025	−7.7	−6.8	−6.5	−6.2	−4.8	−6.40	0.67	0.15	−6.70	−6.09
DB026	−8.0	−6.9	−6.4	−6.1	−5.3	−6.50	0.74	0.16	−6.83	−6.16
DB027	−7.6	−6.8	−6.5	−6.1	−4.9	−6.44	0.70	0.15	−6.76	−6.13
DB028	−7.9	−7.0	−6.5	−6.1	−5.0	−6.50	0.72	0.16	−6.83	−6.18
DB029	−7.9	−7.0	−6.4	−6.0	−4.9	−6.47	0.73	0.16	−6.80	−6.13
DB030	−7.9	−6.8	−6.4	−6.0	−4.9	−6.41	0.69	0.15	−6.72	−6.10
DB031	−7.6	−6.9	−6.5	−6.0	−5.1	−6.52	0.68	0.15	−6.83	−6.21
DB032	−11.1	−8.5	−8.1	−7.5	−6.3	−8.06	1.07	0.23	−8.54	−7.57
DB034	−7.9	−6.7	−6.3	−6.0	−5.1	−6.30	0.74	0.16	−6.64	−5.96
DB035	−7.4	−6.6	−6.2	−5.9	−4.8	−6.17	0.70	0.15	−6.49	−5.85
DB036	−9.6	−8.7	−8.0	−7.3	−6.0	−7.89	1.00	0.22	−8.34	−7.44
DB037	−7.9	−6.6	−6.1	−5.9	−5.1	−6.30	0.78	0.17	−6.65	−5.94
DB038	−9.1	−7.9	−7.7	−6.7	−5.6	−7.41	0.86	0.19	−7.81	−7.02
DC001	−6.3	−6.1	−5.9	−5.2	−4.8	−5.69	0.53	0.11	−5.93	−5.45
DC002	−6.8	−6.1	−5.8	−5.4	−4.9	−5.77	0.57	0.12	−6.03	−5.51
DC003	−6.2	−5.9	−5.6	−4.9	−4.5	−5.48	0.52	0.11	−5.71	−5.24
DC004	−6.2	−6.0	−5.7	−5.2	−4.7	−5.63	0.44	0.10	−5.84	−5.43
DC005	−7.1	−6.4	−5.9	−5.6	−4.8	−5.96	0.61	0.13	−6.23	−5.68
DC006	−7.1	−6.5	−6.1	−5.6	−4.8	−6.01	0.62	0.13	−6.29	−5.73
DC007	−10.1	−8.7	−7.9	−7.1	−6.5	−7.96	1.01	0.22	−8.42	−7.50
DC008	−10.3	−8.6	−8.1	−7.5	−6.8	−8.17	0.91	0.20	−8.58	−7.75
DC009	−5.7	−5.4	−5.1	−4.7	−4.2	−5.06	0.45	0.10	−5.27	−4.86
DC010	−5.4	−4.9	−4.5	−4.3	−3.8	−4.57	0.44	0.10	−4.77	−4.37
DC011	−6.4	−6.0	−5.7	−5.1	−4.6	−5.57	0.54	0.12	−5.81	−5.32
DC012	−6.3	−5.5	−5.0	−4.7	−4.2	−5.04	0.54	0.12	−5.29	−4.80
DC013	−6.8	−5.6	−4.9	−4.5	−3.5	−5.01	0.85	0.19	−5.40	−4.63
DC014	−5.2	−4.4	−4.1	−3.8	−3.6	−4.13	0.43	0.09	−4.33	−3.94
DC015	−4.1	−3.5	−3.3	−3.1	−2.8	−3.30	0.34	0.07	−3.45	−3.14
DC016	−9.1	−7.9	−7.5	−6.7	−5.8	−7.40	0.94	0.21	−7.83	−6.98
DC017	−6.5	−5.6	−5.4	−4.8	−4.6	−5.32	0.49	0.11	−5.54	−5.10
DC018	−3.6	−2.9	−2.8	−2.6	−2.4	−2.81	0.28	0.06	−2.94	−2.68

Compound ID	Min	25% Percentile	Med	75% Percentile	Max	Average Value	SD	SEM	95% Confidence Interval of Mean	
									Lower	Upper
DC019	−5.0	−4.7	−4.4	−4.1	−3.8	−4.41	0.37	0.08	−4.58	−4.25
DC020	−5.2	−4.9	−4.5	−4.2	−3.9	−4.53	0.40	0.09	−4.72	−4.35
DC021	−6.5	−6.0	−5.5	−4.7	−3.7	−5.30	0.91	0.20	−5.72	−4.89
DC022	−6.9	−6.0	−5.4	−4.9	−4.4	−5.52	0.74	0.16	−5.85	−5.18
DC023	−6.4	−5.6	−5.0	−4.5	−3.8	−5.09	0.77	0.17	−5.43	−4.74
DC024	−6.8	−6.4	−6.0	−5.5	−4.8	−5.90	0.61	0.13	−6.18	−5.62
DC025	−9.4	−8.0	−7.3	−6.8	−5.9	−7.45	0.98	0.21	−7.90	−7.01
DC026	−7.7	−6.1	−5.4	−5.2	−4.4	−5.59	0.75	0.16	−5.93	−5.25
DD001	−8.0	−7.2	−7.0	−6.7	−5.6	−6.94	0.68	0.15	−7.25	−6.63
DD002	−9.1	−7.6	−7.4	−6.8	−5.7	−7.21	0.82	0.18	−7.59	−6.84
DD003	−7.5	−6.5	−6.4	−6.0	−5.1	−6.27	0.63	0.14	−6.55	−5.98
DD004	−7.8	−6.9	−6.7	−6.3	−5.0	−6.57	0.65	0.14	−6.86	−6.27
DD005	−9.4	−7.7	−7.3	−6.9	−5.8	−7.30	0.86	0.19	−7.69	−6.92
DD006	−9.6	−7.9	−7.5	−6.8	−5.9	−7.43	0.86	0.19	−7.82	−7.04
DD007	−9.0	−7.5	−7.0	−6.7	−5.7	−7.09	0.83	0.18	−7.47	−6.71
DD008	−7.8	−6.8	−6.4	−6.0	−4.9	−6.37	0.67	0.15	−6.68	−6.07
DD009	−8.3	−7.6	−7.1	−6.3	−5.6	−7.02	0.80	0.18	−7.38	−6.65
DD010	−9.6	−7.6	−7.3	−6.7	−5.4	−7.27	0.99	0.21	−7.72	−6.82
DD011	−8.3	−7.3	−6.8	−6.2	−5.4	−6.78	0.79	0.17	−7.14	−6.42
DD012	−8.9	−7.8	−7.1	−6.7	−5.8	−7.22	0.84	0.18	−7.60	−6.84
DD013	−8.2	−7.1	−6.9	−6.5	−5.2	−6.76	0.83	0.18	−7.14	−6.38
DD014	−8.7	−7.8	−7.3	−6.4	−5.6	−7.16	0.85	0.19	−7.55	−6.77
DE001	−6.2	−5.5	−5.1	−4.4	−4.0	−4.97	0.68	0.15	−5.28	−4.66
DE002	−6.9	−5.5	−5.1	−4.6	−4.0	−5.10	0.71	0.16	−5.42	−4.78
DF001	−8.0	−7.4	−7.0	−6.1	−5.5	−6.82	0.74	0.16	−7.16	−6.48
DF002	−7.5	−7.1	−6.5	−6.0	−5.2	−6.49	0.68	0.15	−6.80	−6.18
DF003	−8.1	−7.1	−6.7	−6.2	−5.5	−6.71	0.75	0.16	−7.05	−6.37
DF004	−7.6	−6.7	−6.1	−5.8	−4.9	−6.26	0.74	0.16	−6.60	−5.93
DF005	−7.9	−6.9	−6.5	−6.0	−5.2	−6.52	0.72	0.16	−6.85	−6.19
DF006	−8.9	−7.9	−7.2	−6.5	−5.5	−7.20	0.88	0.19	−7.60	−6.80
DF007	−8.5	−7.8	−7.0	−6.6	−5.3	−7.14	0.85	0.19	−7.53	−6.75

Compound ID	Min	25% Percentile	Med	75% Percentile	Max	Average Value	SD	SEM	95% Confidence Interval of Mean	
									Lower	Upper
DF008	−9.4	−7.6	−6.9	−6.6	−5.9	−7.12	0.91	0.20	−7.54	−6.71
DF009	−7.8	−7.0	−6.4	−6.1	−5.1	−6.51	0.71	0.15	−6.83	−6.19
DF010	−8.7	−7.4	−7.1	−6.7	−5.1	−6.99	0.74	0.16	−7.33	−6.66
DF011	−7.9	−6.8	−6.3	−5.9	−5.2	−6.40	0.71	0.16	−6.73	−6.08
DF012	−7.9	−7.0	−6.5	−6.0	−5.0	−6.51	0.74	0.16	−6.85	−6.18
DG001	−5.8	−5.4	−5.2	−4.9	−4.4	−5.10	0.38	0.08	−5.27	−4.93
DG002	−5.5	−4.9	−4.7	−4.4	−4.0	−4.69	0.39	0.09	−4.87	−4.51
DG003	−5.8	−5.3	−5.1	−4.8	−4.2	−5.03	0.43	0.09	−5.23	−4.84
DG004	−7.1	−6.0	−5.7	−5.4	−5.0	−5.79	0.50	0.11	−6.02	−5.56
DG005	−6.2	−5.4	−5.2	−4.9	−4.4	−5.17	0.41	0.09	−5.35	−4.98
DG006	−5.4	−4.8	−4.6	−4.5	−4.0	−4.64	0.36	0.08	−4.80	−4.47
DH001	−5.3	−5.0	−4.6	−4.3	−4.0	−4.62	0.41	0.09	−4.81	−4.43
DH002	−5.4	−5.2	−4.7	−4.4	−3.8	−4.73	0.49	0.11	−4.96	−4.51
DH003	−5.3	−4.9	−4.6	−4.3	−4.0	−4.58	0.38	0.08	−4.75	−4.41
DH004	−5.4	−4.9	−4.7	−4.3	−4.1	−4.67	0.41	0.09	−4.86	−4.49
DH005	−7.0	−6.4	−5.8	−5.3	−4.8	−5.82	0.64	0.14	−6.11	−5.53
DH006	−5.4	−4.9	−4.6	−4.3	−3.8	−4.64	0.45	0.10	−4.84	−4.43
DH007	−5.0	−4.5	−4.3	−3.9	−3.4	−4.24	0.40	0.09	−4.42	−4.06
DH008	−5.9	−5.3	−5.1	−4.7	−4.3	−5.11	0.45	0.10	−5.31	−4.91
DH009	−4.7	−4.3	−4.1	−3.9	−3.3	−4.05	0.38	0.08	−4.23	−3.88
DH010	−5.6	−5.1	−4.8	−4.5	−4.0	−4.78	0.41	0.09	−4.96	−4.59
DH011	−4.7	−4.2	−3.9	−3.8	−3.3	−3.97	0.32	0.07	−4.11	−3.82
DH012	−6.1	−5.4	−5.3	−4.9	−4.5	−5.20	0.40	0.09	−5.38	−5.02
DH013	−5.2	−4.8	−4.5	−4.1	−3.7	−4.47	0.45	0.10	−4.67	−4.26
DH014	−7.9	−7.1	−6.8	−6.2	−5.0	−6.56	0.75	0.16	−6.90	−6.22
DH015	−7.0	−6.4	−5.9	−5.3	−4.6	−5.88	0.65	0.14	−6.18	−5.58
DH016	−5.6	−4.6	−4.5	−4.3	−3.9	−4.53	0.41	0.09	−4.72	−4.35
DH017	−4.7	−4.1	−3.9	−3.6	−3.2	−3.88	0.40	0.09	−4.06	−3.69
DI001	−7.6	−7.0	−6.5	−6.0	−5.2	−6.46	0.70	0.15	−6.78	−6.14
DJ001	−8.8	−7.3	−6.7	−5.8	−5.2	−6.67	1.00	0.22	−7.13	−6.21
DJ002	−3.9	−3.6	−3.3	−3.1	−2.8	−3.35	0.29	0.06	−3.48	−3.22

Compound ID	Min	25% Percentile	Med	75% Percentile	Max	Average Value	SD	SEM	95% Confidence Interval of Mean	
									Lower	Upper
DK001	−7.4	−6.0	−5.3	−4.9	−4.1	−5.46	0.79	0.17	−5.82	−5.10
DL001	−6.2	−5.5	−5.2	−5.0	−4.1	−5.24	0.51	0.11	−5.47	−5.01
DL002	−6.1	−5.6	−5.2	−5.0	−4.1	−5.26	0.49	0.11	−5.48	−5.03
DL003	−7.3	−6.5	−5.7	−5.5	−4.7	−5.94	0.66	0.14	−6.24	−5.64
DL004	−7.6	−6.7	−6.0	−5.8	−4.5	−6.20	0.76	0.17	−6.55	−5.85
KA001	−9.7	−8.2	−7.7	−7.0	−5.9	−7.61	0.94	0.21	−8.04	−7.18
KA002	−10.0	−9.5	−7.8	−7.5	−6.6	−8.25	1.16	0.25	−8.78	−7.73
KA003	−9.1	−8.2	−7.8	−6.7	−5.9	−7.57	0.94	0.21	−8.00	−7.14
KA004	−9.4	−8.3	−7.6	−6.8	−6.1	−7.61	0.95	0.21	−8.04	−7.17
KA005	−8.9	−8.0	−7.6	−6.7	−5.8	−7.38	0.94	0.21	−7.81	−6.95
KA006	−8.9	−8.1	−7.5	−6.8	−6.2	−7.48	0.73	0.16	−7.81	−7.14
KA007	−9.0	−8.1	−7.6	−6.7	−6.1	−7.46	0.79	0.17	−7.82	−7.10
KA008	−8.6	−8.0	−7.6	−6.8	−6.1	−7.45	0.74	0.16	−7.79	−7.12
KA009	−8.6	−8.0	−7.7	−7.0	−6.0	−7.51	0.75	0.16	−7.85	−7.17
KA010	−9.3	−8.5	−7.6	−6.6	−6.2	−7.60	0.98	0.21	−8.04	−7.15
KA011	−9.5	−8.6	−8.1	−7.2	−6.4	−7.97	0.91	0.20	−8.38	−7.55
KA012	−9.4	−8.8	−7.9	−7.3	−6.8	−8.02	0.87	0.19	−8.42	−7.62
KA013	−10.3	−9.2	−8.8	−7.8	−6.9	−8.60	0.99	0.22	−9.05	−8.15
KA014	−9.1	−8.8	−8.2	−7.0	−6.6	−7.96	0.91	0.20	−8.37	−7.54
KA015	−9.2	−7.9	−7.6	−6.9	−5.8	−7.40	0.83	0.18	−7.78	−7.03
KA016	−9.4	−8.0	−7.5	−6.7	−6.0	−7.40	0.81	0.18	−7.77	−7.04
KA017	−9.1	−8.0	−7.6	−7.1	−5.7	−7.47	0.84	0.18	−7.85	−7.09
KA018	−9.2	−8.7	−8.3	−7.1	−6.2	−7.98	0.92	0.20	−8.40	−7.56
KA019	−11.0	−8.4	−8.2	−7.8	−7.2	−8.38	1.04	0.23	−8.85	−7.91
KA020	−9.5	−8.1	−7.8	−6.8	−6.0	−7.60	0.94	0.21	−8.03	−7.17
KA021	−9.5	−8.4	−7.9	−7.3	−5.9	−7.82	0.95	0.21	−8.26	−7.39
KA022	−9.2	−8.5	−7.8	−7.0	−5.9	−7.81	0.95	0.21	−8.24	−7.38
KA023	−10.1	−8.4	−7.9	−7.1	−6.4	−7.87	0.98	0.21	−8.31	−7.42
KA024	−8.6	−8.0	−7.3	−6.7	−6.0	−7.28	0.79	0.17	−7.64	−6.92
KA025	−9.4	−7.6	−7.0	−6.6	−5.8	−7.14	0.97	0.21	−7.59	−6.70
KA026	−9.5	−8.3	−7.6	−6.8	−5.9	−7.53	1.01	0.22	−7.99	−7.07

Compound ID	Min	25% Percentile	Med	75% Percentile	Max	Average Value	SD	SEM	95% Confidence Interval of Mean	
									Lower	Upper
KA027	−9.0	−8.4	−7.8	−6.8	−5.8	−7.66	0.90	0.20	−8.07	−7.25
KA028	−8.8	−8.1	−7.5	−6.6	−5.7	−7.42	0.98	0.21	−7.86	−6.97
KA029	−9.2	−8.3	−7.5	−6.9	−6.4	−7.60	0.87	0.19	−8.00	−7.21
KA030	−9.9	−8.3	−8.0	−7.0	−6.4	−7.81	0.92	0.20	−8.23	−7.39
KA031	−8.9	−8.4	−7.5	−6.8	−6.1	−7.56	0.87	0.19	−7.95	−7.16
KA032	−9.3	−8.3	−7.9	−7.1	−6.2	−7.80	0.92	0.20	−8.22	−7.39
KA033	−9.7	−8.0	−7.3	−6.7	−5.9	−7.40	0.99	0.22	−7.85	−6.95
KA034	−8.7	−7.5	−7.2	−6.9	−6.0	−7.23	0.72	0.16	−7.56	−6.90
KA035	−9.2	−8.3	−7.7	−6.9	−6.2	−7.67	0.88	0.19	−8.07	−7.26
KA036	−8.7	−8.2	−7.4	−6.8	−5.8	−7.44	0.86	0.19	−7.83	−7.05
KA037	−8.4	−7.6	−7.2	−6.6	−5.9	−7.13	0.75	0.16	−7.47	−6.79
KA038	−8.8	−8.4	−7.6	−6.7	−5.9	−7.50	0.89	0.19	−7.91	−7.09
KA039	−8.8	−8.1	−7.7	−6.8	−6.5	−7.60	0.73	0.16	−7.93	−7.26
KA040	−9.2	−8.0	−7.1	−6.7	−5.7	−7.37	0.99	0.22	−7.82	−6.92
KA041	−8.6	−8.3	−7.6	−6.9	−6.2	−7.61	0.76	0.17	−7.96	−7.27
KA042	−9.0	−8.1	−7.5	−7.0	−5.8	−7.47	0.86	0.19	−7.86	−7.08
KA043	−8.7	−8.1	−7.4	−6.9	−6.1	−7.48	0.78	0.17	−7.84	−7.12
KA044	−9.2	−8.4	−7.9	−7.0	−6.2	−7.76	0.90	0.20	−8.17	−7.35
KA046	−9.0	−8.2	−7.9	−6.8	−5.8	−7.57	0.88	0.19	−7.97	−7.16
KA047	−9.4	−8.2	−7.7	−7.2	−6.1	−7.64	0.86	0.19	−8.04	−7.25
KA049	−8.7	−8.4	−7.5	−6.5	−5.9	−7.48	0.95	0.21	−7.92	−7.05
KA050	−9.3	−7.4	−7.0	−6.2	−5.3	−6.82	0.93	0.20	−7.25	−6.40
KA051	−8.5	−7.8	−7.4	−6.6	−5.7	−7.31	0.85	0.18	−7.70	−6.93
KA052	−9.2	−8.1	−7.4	−6.5	−5.8	−7.30	1.05	0.23	−7.78	−6.82
KA053	−8.7	−7.8	−7.3	−6.6	−5.4	−7.13	0.97	0.21	−7.57	−6.68
KA054	−8.3	−7.6	−7.0	−6.3	−5.3	−6.90	0.86	0.19	−7.29	−6.51
KA055	−8.6	−8.0	−7.4	−6.8	−6.2	−7.37	0.77	0.17	−7.72	−7.02
KA056	−8.7	−8.1	−7.4	−6.7	−5.6	−7.26	0.95	0.21	−7.69	−6.82
KA057	−8.4	−8.0	−7.5	−6.6	−6.1	−7.31	0.80	0.17	−7.68	−6.95
KA058	−8.6	−7.8	−7.3	−6.4	−5.4	−7.12	0.87	0.19	−7.51	−6.72
KA059	−8.5	−7.7	−7.2	−6.8	−6.0	−7.22	0.77	0.17	−7.57	−6.87

Compound ID	Min	25% Percentile	Med	75% Percentile	Max	Average Value	SD	SEM	95% Confidence Interval of Mean	
									Lower	Upper
KA060	−9.9	−9.2	−8.5	−7.6	−6.8	−8.48	0.90	0.20	−8.89	−8.06
KA061	−8.5	−8.3	−7.4	−6.9	−5.9	−7.50	0.86	0.19	−7.89	−7.10
KA062	−8.5	−7.9	−7.5	−6.7	−5.8	−7.34	0.75	0.16	−7.68	−7.00
KA064	−9.8	−8.8	−8.3	−7.6	−5.8	−8.07	1.15	0.25	−8.59	−7.55
KA065	−10.7	−9.0	−8.6	−7.5	−6.9	−8.46	1.10	0.24	−8.96	−7.96
KA066	−10.3	−8.6	−8.0	−7.2	−6.6	−7.97	0.90	0.20	−8.38	−7.56
KA067	−10.4	−8.9	−8.4	−7.5	−6.4	−8.33	1.03	0.22	−8.80	−7.87
KA068	−8.9	−7.8	−7.1	−6.5	−5.3	−7.08	0.95	0.21	−7.51	−6.64
KA069	−9.6	−8.4	−7.6	−7.1	−6.3	−7.72	0.94	0.20	−8.15	−7.30
KA070	−8.6	−8.1	−7.7	−6.9	−5.8	−7.39	0.84	0.18	−7.77	−7.01
KA071	−9.5	−8.6	−7.8	−7.2	−6.3	−7.88	0.96	0.21	−8.32	−7.44
KA072	−8.9	−7.9	−7.2	−6.7	−5.7	−7.36	0.86	0.19	−7.75	−6.97
KA073	−9.2	−8.2	−7.3	−6.9	−6.0	−7.50	0.93	0.20	−7.92	−7.07
KA074	−9.4	−7.9	−7.3	−6.5	−6.0	−7.34	0.91	0.20	−7.75	−6.92
KA075	−8.5	−7.4	−6.9	−6.5	−5.8	−7.04	0.84	0.18	−7.42	−6.66
KA076	−8.7	−8.1	−7.3	−6.6	−5.5	−7.28	0.82	0.18	−7.66	−6.91
KA077	−8.7	−8.2	−7.1	−6.3	−5.6	−7.17	1.08	0.24	−7.66	−6.68
KA078	−8.7	−7.9	−7.4	−7.0	−5.9	−7.45	0.81	0.18	−7.81	−7.08
KA079	−10.6	−9.4	−8.4	−7.6	−6.6	−8.46	1.05	0.23	−8.94	−7.98
KA080	−8.8	−7.7	−7.4	−6.7	−5.5	−7.24	0.79	0.17	−7.60	−6.88
KA081	−9.3	−8.2	−7.5	−6.6	−5.7	−7.40	0.98	0.21	−7.85	−6.95
KA082	−9.1	−7.7	−7.3	−6.8	−5.7	−7.20	0.93	0.20	−7.63	−6.78
KA083	−8.7	−7.9	−7.4	−6.8	−5.7	−7.36	0.88	0.19	−7.76	−6.96
KA084	−9.1	−7.9	−7.6	−7.0	−6.1	−7.45	0.80	0.18	−7.82	−7.09
KA085	−9.0	−7.6	−7.2	−6.6	−5.8	−7.15	0.80	0.17	−7.52	−6.79
KA086	−10.7	−9.3	−8.3	−7.8	−7.0	−8.52	1.07	0.23	−9.01	−8.03
KA088	−10.9	−8.6	−8.2	−7.3	−6.3	−8.04	1.08	0.23	−8.53	−7.55
KA089	−10.2	−8.6	−8.3	−7.4	−6.3	−7.98	0.93	0.20	−8.40	−7.56
KA090	−12.0	−9.4	−8.4	−8.3	−7.0	−8.66	1.15	0.25	−9.18	−8.14
KA091	−11.6	−9.3	−8.2	−7.3	−6.7	−8.37	1.24	0.27	−8.93	−7.80
KA092	−10.6	−8.9	−8.4	−7.5	−6.7	−8.28	1.03	0.23	−8.75	−7.81

Compound ID	Min	25% Percentile	Med	75% Percentile	Max	Average Value	SD	SEM	95% Confidence Interval of Mean	
									Lower	Upper
KA093	−10.0	−8.6	−8.4	−7.8	−6.9	−8.32	0.88	0.19	−8.72	−7.92
KA094	−10.3	−9.1	−8.1	−7.8	−6.9	−8.29	0.96	0.21	−8.72	−7.85
KA095	−11.3	−9.0	−8.1	−7.4	−6.7	−8.30	1.16	0.25	−8.82	−7.77
KA096	−11.4	−8.5	−8.1	−7.7	−6.4	−8.20	1.08	0.23	−8.69	−7.71
KA097	−10.3	−9.0	−7.9	−7.6	−6.7	−8.16	1.00	0.22	−8.62	−7.70
KA098	−11.3	−9.2	−8.2	−7.3	−6.7	−8.40	1.20	0.26	−8.95	−7.85
KA099	−9.6	−8.3	−8.1	−7.6	−6.7	−8.10	0.81	0.18	−8.46	−7.73
KA100	−11.6	−9.3	−8.6	−7.9	−7.0	−8.62	1.16	0.25	−9.15	−8.09
KA101	−10.8	−9.0	−7.8	−7.2	−6.5	−8.11	1.08	0.23	−8.60	−7.62
KA102	−10.6	−8.8	−8.1	−7.5	−6.5	−8.10	1.07	0.23	−8.59	−7.62
KA103	−9.5	−8.7	−8.3	−7.5	−6.7	−8.08	0.87	0.19	−8.47	−7.68
KA104	−11.1	−8.5	−7.8	−7.4	−5.9	−7.80	1.13	0.25	−8.32	−7.29
KA105	−11.4	−9.2	−8.0	−7.8	−6.7	−8.36	1.12	0.24	−8.87	−7.85
KA106	−11.1	−8.4	−8.1	−7.7	−6.2	−8.10	1.11	0.24	−8.60	−7.60
KA107	−10.3	−9.2	−8.4	−7.6	−6.6	−8.27	1.06	0.23	−8.75	−7.79
KA108	−9.5	−8.3	−7.1	−6.7	−6.0	−7.47	1.03	0.22	−7.94	−7.00
KA109	−9.8	−8.8	−8.0	−7.4	−6.6	−8.14	1.05	0.23	−8.62	−7.67
KA110	−9.7	−8.8	−7.8	−7.0	−6.1	−7.92	1.08	0.24	−8.41	−7.43
KA113	−11.7	−9.7	−8.5	−7.9	−7.2	−8.70	1.20	0.26	−9.25	−8.15
KA114	−8.6	−8.0	−7.6	−7.1	−5.9	−7.46	0.74	0.16	−7.80	−7.13
KA115	−10.1	−8.8	−8.6	−8.1	−7.2	−8.54	0.84	0.18	−8.92	−8.16
KA116	−9.6	−8.4	−7.9	−7.4	−6.1	−7.88	0.95	0.21	−8.31	−7.44
KA120	−8.4	−8.0	−7.4	−6.8	−5.5	−7.36	0.83	0.18	−7.74	−6.98
KA121	−10.2	−8.7	−7.7	−7.2	−6.3	−7.99	0.98	0.21	−8.43	−7.55
KA122	−9.4	−9.0	−7.8	−7.5	−6.6	−8.08	0.91	0.20	−8.49	−7.67
KA126	−8.6	−7.2	−6.8	−6.3	−5.5	−6.77	0.74	0.16	−7.11	−6.43
KA127	−8.9	−7.8	−7.3	−6.5	−5.9	−7.34	0.90	0.20	−7.75	−6.93
KA128	−8.7	−8.0	−7.0	−6.5	−6.0	−7.22	0.83	0.18	−7.59	−6.84
KA129	−8.9	−7.9	−7.0	−6.3	−5.7	−7.16	0.89	0.19	−7.56	−6.75
KA130	−8.6	−8.0	−7.6	−7.0	−6.2	−7.58	0.69	0.15	−7.89	−7.26
KA131	−10.4	−8.9	−8.2	−7.4	−6.5	−8.24	1.07	0.23	−8.72	−7.75

Compound ID	Min	25% Percentile	Med	75% Percentile	Max	Average Value	SD	SEM	95% Confidence Interval of Mean	
									Lower	Upper
KA132	−8.0	−7.8	−7.1	−6.6	−5.9	−7.14	0.67	0.15	−7.44	−6.83
KA133	−9.5	−8.2	−6.8	−5.9	−5.2	−7.10	1.37	0.30	−7.72	−6.47
KA134	−10.3	−8.4	−7.7	−6.5	−4.9	−7.55	1.43	0.31	−8.20	−6.90
KA135	−9.8	−8.2	−7.6	−6.8	−5.7	−7.61	0.96	0.21	−8.05	−7.17
KA136	−9.2	−8.4	−7.5	−7.3	−6.0	−7.66	0.88	0.19	−8.06	−7.25
KA137	−9.0	−8.5	−7.9	−7.2	−6.2	−7.74	0.83	0.18	−8.12	−7.37
KA138	−9.2	−8.2	−7.9	−7.2	−5.9	−7.76	0.81	0.18	−8.13	−7.39
KA139	−10.0	−8.6	−8.3	−7.0	−5.9	−7.90	1.11	0.24	−8.40	−7.39
KA142	−8.9	−8.4	−7.8	−6.8	−6.0	−7.66	0.91	0.20	−8.07	−7.25
KA144	−10.3	−8.8	−8.1	−7.7	−6.7	−8.29	1.05	0.23	−8.77	−7.81
KA145	−10.5	−9.1	−8.1	−7.7	−6.7	−8.38	1.07	0.23	−8.86	−7.89
KA146	−8.8	−8.0	−7.9	−6.7	−6.1	−7.47	0.82	0.18	−7.84	−7.10
KA148	−10.0	−8.7	−8.2	−7.2	−5.9	−7.94	1.12	0.25	−8.45	−7.43
KA149	−10.3	−8.9	−8.2	−7.3	−6.5	−8.16	1.12	0.24	−8.67	−7.65
KA150	−9.2	−8.4	−7.9	−7.1	−6.0	−7.82	0.87	0.19	−8.22	−7.43
KA151	−8.7	−8.1	−7.4	−6.8	−6.0	−7.35	0.88	0.19	−7.75	−6.95
KA152	−8.7	−7.6	−7.2	−6.5	−5.3	−7.09	0.94	0.21	−7.52	−6.66
KA153	−10.1	−8.3	−7.9	−7.4	−6.4	−8.06	0.99	0.22	−8.51	−7.61
KA154	−7.1	−6.8	−6.3	−5.9	−4.8	−6.26	0.66	0.14	−6.56	−5.96
KA155	−8.5	−8.2	−7.7	−6.8	−6.1	−7.45	0.79	0.17	−7.81	−7.09
KA156	−9.0	−8.0	−7.3	−6.9	−5.6	−7.42	0.93	0.20	−7.85	−7.00
KA157	−8.9	−8.3	−7.9	−7.0	−6.6	−7.78	0.75	0.16	−8.12	−7.44
KA158	−9.5	−8.3	−8.0	−7.3	−6.1	−7.78	0.84	0.18	−8.16	−7.39
KA159	−11.1	−8.7	−8.3	−7.2	−6.3	−8.08	1.09	0.24	−8.58	−7.59
KA160	−10.4	−9.3	−8.4	−7.5	−6.6	−8.36	1.13	0.25	−8.87	−7.85
KA161	−9.3	−8.1	−7.6	−7.2	−5.9	−7.56	0.84	0.18	−7.94	−7.18
KA162	−9.6	−7.9	−7.6	−7.0	−5.7	−7.59	0.90	0.20	−7.99	−7.18
KA163	−8.7	−8.2	−7.5	−6.8	−5.4	−7.45	0.84	0.18	−7.84	−7.07
KA164	−8.8	−8.1	−7.5	−6.7	−6.0	−7.48	0.83	0.18	−7.86	−7.10
KA165	−11.4	−10.4	−9.2	−8.8	−7.4	−9.40	1.14	0.25	−9.92	−8.88
KA166	−8.0	−7.5	−7.0	−6.3	−5.5	−6.89	0.78	0.17	−7.24	−6.54

Compound ID	Min	25% Percentile	Med	75% Percentile	Max	Average Value	SD	SEM	95% Confidence Interval of Mean	
									Lower	Upper
KA168	−10.1	−8.6	−8.1	−7.2	−6.5	−8.07	1.04	0.23	−8.54	−7.60
KA169	−9.2	−7.7	−7.3	−6.5	−5.6	−7.24	0.95	0.21	−7.67	−6.80
KA170	−8.9	−8.5	−7.7	−6.9	−6.3	−7.65	0.85	0.19	−8.04	−7.26
KA171	−9.6	−8.1	−7.8	−7.2	−5.9	−7.65	0.79	0.17	−8.01	−7.29
KA172	−9.4	−8.0	−7.5	−7.2	−6.0	−7.53	0.79	0.17	−7.89	−7.17
KA173	−11.4	−9.1	−8.1	−7.8	−6.7	−8.43	1.15	0.25	−8.95	−7.91
KA174	−9.1	−8.2	−7.6	−6.8	−6.0	−7.62	0.99	0.22	−8.08	−7.17
KA175	−10.9	−8.7	−7.8	−7.4	−6.5	−8.09	1.17	0.25	−8.62	−7.55
KA176	−11.4	−8.7	−8.2	−7.5	−6.1	−8.09	1.15	0.25	−8.62	−7.57
KA177	−9.7	−8.1	−7.7	−6.9	−5.8	−7.51	0.94	0.21	−7.94	−7.08
KA178	−9.1	−8.2	−7.6	−6.7	−6.3	−7.56	0.85	0.18	−7.95	−7.18
KA179	−11.4	−9.4	−8.4	−7.8	−6.5	−8.55	1.30	0.28	−9.15	−7.96
KB001	−8.8	−7.5	−6.9	−6.0	−5.4	−6.90	0.96	0.21	−7.34	−6.46
KB002	−8.5	−7.4	−7.1	−6.0	−5.2	−6.90	0.95	0.21	−7.33	−6.46
KB003	−9.8	−8.0	−7.2	−6.3	−5.6	−7.18	1.04	0.23	−7.65	−6.71
KB004	−8.5	−7.9	−6.9	−6.3	−5.2	−6.98	0.95	0.21	−7.41	−6.54
KB005	−9.7	−8.0	−6.8	−6.2	−5.2	−7.03	1.08	0.24	−7.53	−6.54
KB006	−9.4	−7.7	−7.3	−6.9	−5.8	−7.24	0.93	0.20	−7.66	−6.82
KB007	−9.3	−8.1	−7.4	−6.6	−5.7	−7.32	1.00	0.22	−7.78	−6.87
KB008	−8.2	−7.7	−7.0	−6.5	−5.5	−6.99	0.85	0.18	−7.37	−6.60
KB009	−9.4	−7.6	−7.2	−6.5	−5.6	−7.18	0.95	0.21	−7.62	−6.75
KB010	−8.8	−7.4	−7.0	−6.3	−5.4	−6.90	0.89	0.19	−7.30	−6.49
KB013	−8.6	−7.8	−7.1	−6.6	−5.2	−7.11	0.95	0.21	−7.54	−6.68
KB014	−8.8	−7.5	−7.0	−6.2	−5.7	−7.06	0.92	0.20	−7.48	−6.64
KB015	−9.2	−7.8	−7.4	−6.9	−5.8	−7.33	1.00	0.22	−7.78	−6.87
KB016	−9.7	−7.8	−7.4	−6.7	−5.8	−7.40	0.97	0.21	−7.84	−6.95
KB017	−9.1	−7.5	−6.8	−6.1	−5.3	−6.87	0.98	0.21	−7.31	−6.42
KB019	−8.3	−7.5	−7.0	−6.6	−5.3	−6.92	0.82	0.18	−7.29	−6.55
KB020	−9.1	−7.7	−7.3	−6.3	−5.7	−7.23	1.03	0.22	−7.70	−6.76
KB023	−7.8	−6.7	−6.3	−5.9	−4.7	−6.30	0.92	0.20	−6.72	−5.88
KB024	−7.8	−6.9	−6.3	−5.7	−4.9	−6.28	0.92	0.20	−6.70	−5.86

Compound ID	Min	25% Percentile	Med	75% Percentile	Max	Average Value	SD	SEM	95% Confidence Interval of Mean	
									Lower	Upper
KB025	−8.9	−7.6	−7.2	−6.2	−5.3	−6.99	1.01	0.22	−7.44	−6.53
KB026	−8.2	−7.7	−6.8	−5.9	−5.3	−6.86	0.98	0.21	−7.30	−6.41
KB027	−8.4	−6.8	−6.3	−6.1	−5.2	−6.44	0.81	0.18	−6.80	−6.07
KB028	−9.7	−8.0	−7.3	−6.3	−5.4	−7.29	1.09	0.24	−7.78	−6.79
KB029	−9.9	−7.9	−7.2	−6.4	−5.4	−7.19	1.05	0.23	−7.67	−6.71
KB030	−12.3	−10.5	−9.6	−8.3	−7.0	−9.39	1.44	0.31	−10.05	−8.74
KB031	−12.5	−11.0	−10.0	−8.5	−8.0	−9.80	1.38	0.30	−10.42	−9.17
KB032	−11.9	−10.4	−9.2	−8.2	−6.3	−9.26	1.42	0.31	−9.91	−8.61
KB033	−11.3	−10.1	−9.3	−8.7	−7.5	−9.43	1.03	0.22	−9.90	−8.96
KB034	−12.1	−9.7	−8.7	−8.3	−7.7	−9.22	1.26	0.28	−9.79	−8.64
KB035	−11.0	−9.3	−8.4	−7.5	−6.6	−8.50	1.27	0.28	−9.08	−7.93
KB036	−9.1	−7.8	−7.1	−6.1	−5.4	−7.00	1.00	0.22	−7.45	−6.54
KB038	−9.1	−8.0	−7.1	−6.3	−5.5	−7.18	0.97	0.21	−7.62	−6.73
KB039	−8.8	−7.3	−7.0	−6.4	−5.6	−7.00	0.82	0.18	−7.38	−6.63
KB040	−8.1	−7.4	−6.7	−6.0	−5.1	−6.68	0.86	0.19	−7.07	−6.29
KB041	−9.6	−7.9	−7.4	−6.6	−5.3	−7.32	1.02	0.22	−7.78	−6.85
KB042	−8.5	−8.0	−7.6	−6.7	−5.6	−7.39	0.78	0.17	−7.74	−7.03
KB043	−8.9	−7.9	−7.2	−6.4	−5.4	−7.12	0.92	0.20	−7.54	−6.70
KB044	−8.8	−7.8	−7.1	−6.4	−5.3	−7.14	0.82	0.18	−7.51	−6.76
KB045	−8.8	−7.5	−7.0	−6.3	−5.5	−6.99	0.86	0.19	−7.38	−6.59
KB046	−9.1	−7.6	−7.3	−6.5	−5.6	−7.11	0.89	0.19	−7.52	−6.70
KB047	−9.1	−8.0	−7.3	−6.5	−5.4	−7.30	0.94	0.20	−7.73	−6.88
KB050	−9.4	−8.7	−8.3	−7.3	−6.4	−8.08	0.92	0.20	−8.50	−7.66
KC001	−10.1	−9.0	−8.1	−7.2	−6.4	−8.11	1.10	0.24	−8.61	−7.61
KC002	−10.1	−8.4	−7.9	−7.2	−6.5	−7.95	0.99	0.22	−8.41	−7.50
KC003	−10.6	−8.7	−8.1	−7.5	−6.8	−8.30	1.07	0.23	−8.79	−7.81
KC004	−10.0	−9.1	−8.5	−7.5	−6.7	−8.34	0.99	0.22	−8.79	−7.89
KC005	−9.1	−8.3	−7.9	−7.3	−6.3	−7.83	0.78	0.17	−8.18	−7.47
KC006	−8.5	−7.5	−6.7	−6.6	−5.3	−6.94	0.80	0.17	−7.31	−6.58
KC007	−9.9	−8.8	−8.2	−7.5	−6.6	−8.23	0.90	0.20	−8.64	−7.82
KC008	−10.0	−8.8	−8.2	−7.2	−6.5	−8.09	1.00	0.22	−8.54	−7.63

Compound ID	Min	25% Percentile	Med	75% Percentile	Max	Average Value	SD	SEM	95% Confidence Interval of Mean	
									Lower	Upper
KC009	−9.4	−8.0	−7.5	−7.2	−6.5	−7.70	0.79	0.17	−8.06	−7.35
KC010	−8.6	−8.1	−7.4	−6.5	−5.7	−7.35	0.86	0.19	−7.74	−6.96
KD001	−8.5	−7.4	−6.5	−6.2	−5.1	−6.80	1.01	0.22	−7.27	−6.34
KD002	−8.3	−7.2	−6.5	−6.3	−5.1	−6.73	0.88	0.19	−7.13	−6.33
KE001	−9.7	−8.0	−7.6	−7.1	−6.1	−7.53	0.90	0.20	−7.94	−7.13
KE002	−9.3	−8.6	−7.8	−7.6	−6.0	−8.00	0.80	0.18	−8.37	−7.63
KE003	−10.7	−8.5	−7.6	−7.3	−6.3	−7.89	1.05	0.23	−8.37	−7.41
KE004	−10.6	−9.0	−8.2	−7.7	−6.8	−8.39	1.04	0.23	−8.86	−7.91
KE005	−9.8	−8.8	−8.3	−7.3	−6.8	−8.20	0.91	0.20	−8.61	−7.79
KE006	−10.3	−8.4	−7.6	−7.1	−6.2	−7.78	1.03	0.23	−8.25	−7.31
KE007	−10.9	−9.0	−8.3	−8.1	−6.9	−8.60	0.94	0.20	−9.03	−8.18
KE008	−11.4	−9.2	−8.5	−7.9	−6.7	−8.61	1.14	0.25	−9.13	−8.10
KE009	−10.0	−8.9	−8.3	−7.8	−6.5	−8.32	0.91	0.20	−8.73	−7.90
KE010	−10.8	−9.5	−8.1	−7.7	−6.4	−8.36	1.25	0.27	−8.93	−7.79
KE011	−10.3	−8.8	−7.8	−7.4	−6.2	−8.03	1.08	0.24	−8.52	−7.54
KE012	−10.8	−9.3	−8.2	−7.7	−7.0	−8.53	1.04	0.23	−9.00	−8.05
KF001	−6.4	−6.0	−5.7	−5.2	−4.6	−5.61	0.50	0.11	−5.84	−5.38
KF002	−6.9	−6.5	−6.0	−5.6	−4.7	−5.97	0.62	0.13	−6.25	−5.69
KF003	−6.4	−5.8	−5.5	−5.2	−4.5	−5.49	0.48	0.10	−5.71	−5.27
KF004	−7.2	−6.3	−6.0	−5.7	−5.1	−6.04	0.59	0.13	−6.31	−5.77
KF005	−7.2	−6.5	−6.2	−5.8	−5.2	−6.17	0.55	0.12	−6.42	−5.92
KF006	−7.0	−6.3	−6.1	−5.9	−5.2	−6.06	0.44	0.10	−6.26	−5.86
KF007	−8.3	−7.5	−7.0	−6.5	−6.1	−7.11	0.71	0.16	−7.43	−6.79
KG001	−9.8	−8.4	−7.9	−7.5	−6.6	−7.97	0.77	0.17	−8.32	−7.62
KH001	−6.4	−5.5	−5.2	−4.5	−3.8	−5.00	0.79	0.17	−5.36	−4.64
KI001	−7.2	−6.7	−6.2	−5.8	−4.9	−6.18	0.69	0.15	−6.49	−5.86
KJ001	−8.4	−7.2	−6.5	−5.8	−5.4	−6.59	0.90	0.20	−7.00	−6.18
ZA01	−10.6	−8.5	−8.1	−7.4	−6.9	−8.17	0.95	0.21	−8.60	−7.74
ZA02	−10.4	−8.9	−8.3	−7.7	−7.2	−8.37	0.94	0.21	−8.80	−7.94
ZA03	−10.7	−8.9	−8.4	−7.6	−6.7	−8.38	0.98	0.21	−8.82	−7.93
ZA04	−10.7	−9.2	−8.3	−7.6	−7.2	−8.52	1.10	0.24	−9.02	−8.02

Compound ID	Min	25% Percentile	Med	75% Percentile	Max	Average Value	SD	SEM	95% Confidence Interval of Mean	
									Lower	Upper
ZA05	−10.2	−9.2	−8.3	−7.5	−6.6	−8.30	1.00	0.22	−8.76	−7.84
ZA06	−10.6	−9.2	−8.4	−7.6	−7.2	−8.39	0.94	0.20	−8.82	−7.96
ZA07	−10.1	−8.6	−8.1	−7.6	−6.5	−8.20	0.97	0.21	−8.64	−7.76
ZA08	−11.0	−10.5	−9.0	−8.0	−7.1	−9.12	1.30	0.28	−9.72	−8.53
ZA09	−10.5	−9.6	−9.0	−8.0	−7.3	−8.79	0.99	0.22	−9.24	−8.33
ZA11	−9.7	−8.8	−8.2	−7.4	−6.5	−8.17	0.93	0.20	−8.59	−7.74
ZA12	−10.5	−9.2	−8.6	−7.8	−7.4	−8.67	1.00	0.22	−9.12	−8.21
ZA13	−10.1	−8.7	−7.9	−7.5	−6.4	−8.09	1.02	0.22	−8.55	−7.63
ZA14	−9.9	−8.6	−8.4	−7.6	−7.2	−8.34	0.74	0.16	−8.67	−8.00
ZA15	−9.7	−8.9	−8.1	−7.5	−6.9	−8.21	0.82	0.18	−8.59	−7.84
ZA16	−11.2	−9.3	−8.4	−7.8	−6.7	−8.61	1.11	0.24	−9.12	−8.11
ZA17	−10.7	−9.4	−8.8	−7.8	−6.9	−8.66	1.07	0.23	−9.14	−8.17
ZA19	−10.3	−9.2	−8.6	−7.5	−6.4	−8.50	1.23	0.27	−9.06	−7.94
ZA20	−10.2	−8.7	−7.7	−7.2	−6.5	−8.07	1.13	0.25	−8.58	−7.55
ZA21	−9.2	−8.4	−8.0	−7.6	−6.4	−7.90	0.71	0.15	−8.23	−7.58
ZA22	−11.0	−9.2	−8.6	−7.9	−7.2	−8.67	0.94	0.20	−9.09	−8.24
ZA23	−9.4	−8.7	−8.2	−7.8	−6.9	−8.19	0.69	0.15	−8.51	−7.88
ZA24	−10.2	−9.0	−8.6	−7.7	−7.3	−8.43	0.87	0.19	−8.83	−8.04
ZA25	−11.1	−9.2	−8.3	−8.0	−7.2	−8.66	1.22	0.27	−9.22	−8.11
ZA26	−10.8	−9.3	−8.6	−7.6	−7.2	−8.59	1.04	0.23	−9.06	−8.11
ZB01	−8.5	−6.7	−6.1	−5.7	−4.8	−6.25	0.93	0.20	−6.67	−5.82
ZB02	−9.4	−7.6	−7.1	−6.5	−5.4	−7.03	0.97	0.21	−7.47	−6.59
ZB03	−6.4	−5.7	−5.0	−4.5	−4.1	−5.10	0.73	0.16	−5.44	−4.77
ZB04	−6.3	−5.5	−5.0	−4.6	−3.7	−5.01	0.78	0.17	−5.37	−4.65
ZB05	−6.3	−5.2	−4.8	−4.4	−3.9	−4.88	0.60	0.13	−5.15	−4.60
ZB06	−6.3	−5.4	−5.1	−5.0	−3.9	−5.21	0.62	0.13	−5.50	−4.93
ZB07	−6.2	−5.4	−4.7	−4.5	−3.7	−4.90	0.71	0.15	−5.22	−4.57
ZB08	−10.3	−8.2	−7.5	−7.0	−5.8	−7.71	1.18	0.26	−8.25	−7.17
ZB09	−6.3	−5.6	−5.0	−4.6	−3.7	−5.03	0.69	0.15	−5.35	−4.72
ZB10	−10.1	−8.4	−7.8	−6.6	−5.8	−7.73	1.30	0.28	−8.32	−7.14
ZB11	−7.0	−5.9	−5.5	−4.7	−4.2	−5.42	0.82	0.18	−5.79	−5.05

Compound ID	Min	25% Percentile	Med	75% Percentile	Max	Average Value	SD	SEM	95% Confidence Interval of Mean	
									Lower	Upper
ZB12	−6.1	−5.4	−4.9	−4.2	−3.6	−4.85	0.68	0.15	−5.16	−4.54
ZB13	−6.4	−5.6	−5.1	−4.4	−3.6	−4.98	0.81	0.18	−5.35	−4.61
ZB14	−6.5	−5.7	−5.2	−4.6	−3.9	−5.17	0.70	0.15	−5.49	−4.85
ZB15	−6.9	−5.7	−5.2	−5.0	−3.4	−5.15	0.82	0.18	−5.52	−4.78
ZB16	−8.5	−6.7	−6.2	−5.3	−4.8	−6.09	0.92	0.20	−6.50	−5.67
ZB17	−7.6	−5.9	−5.4	−4.8	−4.2	−5.46	0.89	0.19	−5.87	−5.06
ZB18	−6.3	−5.7	−4.9	−4.4	−3.9	−5.01	0.72	0.16	−5.34	−4.68
ZB19	−6.2	−5.3	−4.9	−4.5	−3.5	−4.90	0.73	0.16	−5.23	−4.57
ZB20	−9.6	−7.8	−7.3	−6.1	−5.5	−7.21	1.12	0.25	−7.72	−6.70
ZB21	−10.3	−8.3	−7.9	−7.0	−6.1	−7.82	1.07	0.23	−8.31	−7.34
ZB22	−6.4	−5.5	−5.2	−4.7	−4.3	−5.20	0.59	0.13	−5.47	−4.93
ZB23	−6.7	−6.0	−5.5	−4.9	−3.9	−5.48	0.74	0.16	−5.81	−5.14
ZB24	−6.1	−5.2	−4.9	−4.6	−3.8	−4.92	0.66	0.14	−5.22	−4.62
ZB25	−9.9	−8.6	−7.3	−6.5	−5.7	−7.48	1.22	0.27	−8.04	−6.92
ZB26	−10.0	−9.0	−8.1	−6.8	−6.0	−8.00	1.26	0.27	−8.57	−7.42
ZB27	−13.3	−8.5	−7.8	−6.8	−6.4	−8.04	1.56	0.34	−8.75	−7.33
ZB28	−9.3	−8.2	−7.3	−6.6	−5.7	−7.42	1.04	0.23	−7.90	−6.95
ZC01	−9.2	−8.1	−7.5	−6.4	−5.7	−7.28	1.01	0.22	−7.74	−6.82
ZC02	−8.8	−8.2	−7.4	−6.2	−5.5	−7.18	1.00	0.22	−7.63	−6.73
ZC03	−9.3	−8.2	−7.1	−6.4	−5.8	−7.23	0.93	0.20	−7.65	−6.81
ZC04.	−9.2	−7.9	−7.4	−6.6	−6.0	−7.37	0.90	0.20	−7.78	−6.96
ZC04	−10.0	−7.9	−7.4	−6.6	−6.0	−7.42	1.06	0.23	−7.90	−6.94
ZC05	−10.6	−8.3	−7.7	−6.4	−6.0	−7.60	1.22	0.27	−8.16	−7.04
ZC06	−8.8	−8.1	−7.3	−6.7	−5.5	−7.31	0.95	0.21	−7.75	−6.88
ZC07	−10.0	−8.5	−7.5	−6.8	−5.7	−7.68	1.20	0.26	−8.22	−7.13
ZC08	−8.9	−7.3	−6.9	−6.3	−5.3	−6.86	0.93	0.20	−7.28	−6.43
ZC09	−8.4	−7.5	−7.2	−6.4	−5.3	−6.98	0.86	0.19	−7.37	−6.59
ZC10	−10.3	−8.4	−7.8	−6.8	−5.8	−7.72	1.24	0.27	−8.29	−7.16
ZC11	−9.5	−8.1	−7.6	−7.0	−5.6	−7.55	0.97	0.21	−7.99	−7.11
ZC12	−12.1	−9.6	−9.2	−8.3	−7.2	−9.22	1.50	0.33	−9.91	−8.54
ZC13	−10.2	−8.2	−7.6	−6.6	−5.8	−7.62	1.29	0.28	−8.21	−7.04

Compound ID	Min	25% Percentile	Med	75% Percentile	Max	Average Value	SD	SEM	95% Confidence Interval of Mean	
									Lower	Upper
ZD01	−8.1	−6.7	−6.3	−5.8	−5.0	−6.39	0.77	0.17	−6.74	−6.04
ZD02	−7.3	−6.2	−5.8	−5.5	−4.6	−5.87	0.70	0.15	−6.19	−5.55
ZE01	−9.9	−8.2	−7.2	−6.7	−5.3	−7.51	1.22	0.27	−8.07	−6.96
ZE02	−10.4	−8.6	−8.0	−7.2	−6.4	−7.99	0.91	0.20	−8.40	−7.57
ZF01	−4.4	−3.9	−3.5	−3.4	−3.0	−3.61	0.34	0.07	−3.76	−3.46
ZF02	−5.5	−4.9	−4.6	−4.4	−4.2	−4.71	0.42	0.09	−4.90	−4.52
ZF03	−5.0	−4.5	−4.3	−4.0	−3.4	−4.25	0.41	0.09	−4.43	−4.06
ZF04	−6.7	−6.3	−5.9	−5.3	−4.6	−5.82	0.59	0.13	−6.09	−5.55
ZF05	−5.9	−5.2	−5.1	−4.6	−4.3	−5.05	0.46	0.10	−5.26	−4.84
ZF06	−5.2	−4.7	−4.5	−4.1	−3.7	−4.43	0.45	0.10	−4.63	−4.22
ZF07	−4.7	−4.3	−4.0	−3.8	−3.3	−4.01	0.34	0.07	−4.16	−3.85
ZF08	−5.3	−4.9	−4.7	−4.4	−3.8	−4.67	0.39	0.08	−4.84	−4.49
ZF09	−8.0	−6.9	−6.6	−6.1	−5.0	−6.47	0.71	0.16	−6.80	−6.15
ZF10	−5.8	−5.2	−4.8	−4.5	−3.8	−4.84	0.52	0.11	−5.08	−4.60
ZF11	−5.3	−5.0	−4.5	−4.3	−3.9	−4.62	0.39	0.09	−4.80	−4.44
ZF12	−5.4	−5.0	−4.6	−4.4	−3.7	−4.62	0.46	0.10	−4.83	−4.41
ZF13	−5.3	−4.9	−4.6	−4.2	−4.0	−4.58	0.40	0.09	−4.76	−4.40
ZF14	−6.6	−6.1	−5.8	−5.5	−4.5	−5.76	0.57	0.12	−6.02	−5.50
ZF15	−5.4	−4.7	−4.3	−4.3	−3.9	−4.50	0.39	0.08	−4.67	−4.32
ZF16	−4.7	−4.5	−4.1	−3.9	−3.5	−4.17	0.35	0.08	−4.33	−4.01
ZF17	−5.7	−5.1	−4.7	−4.5	−4.0	−4.82	0.43	0.09	−5.02	−4.63
ZF18	−6.0	−5.4	−5.2	−5.0	−4.6	−5.23	0.33	0.07	−5.38	−5.08
ZG01	−7.5	−6.6	−6.2	−5.8	−5.1	−6.25	0.65	0.14	−6.55	−5.95
ZG02	−7.9	−7.1	−6.8	−6.2	−5.5	−6.67	0.66	0.14	−6.97	−6.37
ZG03	−8.0	−7.2	−6.6	−5.8	−5.3	−6.51	0.79	0.17	−6.87	−6.15
ZG04	−7.5	−6.8	−6.2	−5.8	−5.2	−6.29	0.62	0.14	−6.57	−6.00
ZG05	−7.6	−6.5	−6.1	−5.7	−5.0	−6.18	0.75	0.16	−6.52	−5.84
ZG06	−8.2	−7.3	−6.6	−6.1	−5.4	−6.75	0.77	0.17	−7.10	−6.40
ZH01	−5.7	−5.3	−5.2	−4.7	−4.4	−5.07	0.37	0.08	−5.24	−4.90
ZH02	−6.2	−5.4	−5.2	−4.9	−4.4	−5.16	0.43	0.09	−5.36	−4.97
ZH03	−5.4	−5.0	−4.7	−4.4	−3.9	−4.65	0.38	0.08	−4.82	−4.48

Compound ID	Min	25% Percentile	Med	75% Percentile	Max	Average Value	SD	SEM	95% Confidence Interval of Mean	
									Lower	Upper
ZH04	−5.9	−5.3	−5.1	−4.8	−4.2	−5.06	0.41	0.09	−5.24	−4.87
ZI01	−6.8	−5.9	−5.5	−4.9	−4.2	−5.43	0.74	0.16	−5.76	−5.09
ZI02	−6.2	−5.6	−5.2	−4.7	−3.9	−5.13	0.66	0.14	−5.43	−4.83
ZI03	−6.9	−6.1	−5.4	−5.0	−4.5	−5.54	0.68	0.15	−5.85	−5.23
ZI04	−8.9	−8.0	−7.7	−6.8	−5.8	−7.53	0.85	0.18	−7.91	−7.14
ZJ01	−8.5	−7.4	−6.7	−5.9	−4.6	−6.68	1.01	0.22	−7.14	−6.22
ZJ02	−8.2	−7.0	−6.6	−6.1	−5.2	−6.66	0.76	0.17	−7.00	−6.31
ZJ03	−8.2	−7.4	−6.4	−5.9	−5.4	−6.63	0.82	0.18	−7.01	−6.26

Note: Ave: Average value; Max: Maximum; Med: Median value; Min: Minimum; SD: Standard deviation; SEM: Standard error of mean. Corresponding compound names refer to Supplementary Table S1 online.

References

Cai, W., Xiong, Y., Sheng, Z., Xia, M., & Cui, M. (2013). Determination of eighteen trace elements in Fritillaria from different places by ICP-OES. *Chinese Journal of Modern Applied Pharmacy, 30*(3), 277-280.

Cao, Y., Zhu, X., & Tan, L. (2012). Analysis of chemical composition by GC/MS and antimicrobial activities of essential oil from Fritillaria thunbergii Miq. *Journal of Zhejiang Sci-Tech University, 29*(1), 129-132.

Chen, J. (2017). Determination of trace elements in Angelica sinensis and its processed products. *For all Health, 11*(25), 42-43.

Chen, J., Yang, X., Tang, F., & Wei, Y. (2017). Ultrasonic extraction of matrine and oxymatrine from Sophora Flavescens. *Guangdong Chemical Industry, 44*(343), 62-63. https://doi.org/10.3969/j.issn.1007-1865.2017.05.028

Chen, X., Li, X., Mao, X., Huang, H., Miao, J., & Gao, W. (2016). Study on the effects of different drying methods on physicochemical properties, structure, and in vitro digestibility of Fritillaria thunbergii Miq. (Zhebeimu) flours. *Food and Bioproducts Processing, 98,* 266-274. https://doi.org/10.1016/j.fbp.2016.01.008

Chen, Y., & Wang, S. (2012). β-D-glu 4-1β-D-gal and sucrose determination of different Fritillarias by HPLC-ELSD. *Lishizhen Medicine and Materia Medica Research, 23*(7), 1605-1606.

Chen, Y., Yang, H., Xu, H., Ma, H., He, L., Zhao, X., Gu, F., Gao, Y., & Shan, J. (2018). Isolation, physicochemical properties and immunomodulating effects of polysaccharides from Sophorae Flavescentis Radix. *Journal of International Pharmaceutical Research, 45*(8), 611-618. https://doi.org/10.13220/j.cnki.jipr.2018.08.009

Cheng, X., Xiao, X., Zhang, N., & Ma, S. (2008). Determination the contents of peimine and peiminine in Thumberg Fritillary Bulb by HPLC-MS. *China Pharmacist, 11*(7), 770-772.

Cui, M., Zhang, J., Chen, S., Jiang, H., Zhou, H., & Ling, Q. (2016). Identification of alkaloids and flavonoids in all parts of Fritillaria thunbergii using LC-LTQ-Qrbitrap MS^n. *Zhongguo Zhong Yao Za Zhi, 41*(11), 2124-2130.

Cui, Q., Li, Y., & Liu, X. (2017). Measurement and health risk assessment of microelement of Angelica sinensis. *Bulletin of Disease Control & Prevention (China), 32*(4), 65-67. https://doi.org/10.13215/j.cnki.jbyfkztb.1703009

Ding, Q. (2016). The study on the correlation of the microstructure and chemical composition of Radix Salviae miltiorrhizae, Radix Stellariae and Radix Angelicae SInensis [Master's thesis, Guangzhou University of Chinese Medicine]. China National Knowledge Infrastructure.

Du, W., Zhang, H., Yue, X., Zhu, T., & Ge, W. (2018). The analysis on volatile components of Zhejiang Fritillary slices with different primary processing methods. *Lishizhen Medicine and Materia Medica Research, 29*(1), 73-76.

Duan, B., Huang, L., & Chen, S. (2012). Study on the destructive effect to inherent quality of Fritillaria thunbergii Miq. (Zhebeimu) by sulfur-fumigated process using chromatographic fingerprinting analysis. *Phytomedicine, 19*(6), 562-568. https://doi.org/10.1016/j.phymed.2011.12.010

Filipiak-Szok, A., Kurzawa, M., & Szyk, E. (2016). Simultaneous determination of selected anti-nutritional components in Asiatic plants using ion chromatography. *European Food Research and Technology, 242*(9), 1515-1521. https://doi.org/10.1007/s00217-016-2652-x

Gao, C. (2016). Determination of components in Angelica sinensis and its preparations. *World Latest Medicine Information, 3*(16), 207-208. https://doi.org/10.3969/j.issn.1671-3141.2016.3.154

Gong, W., Zhou, Y., Li, X., Gao, X., Tian, J., Qin, X., & Du, G. (2016). Neuroprotective and cytotoxic phthalides from Angelicae Sinensis Radix. *Molecules, 21*(5), 549-549. https://doi.org/10.3390/molecules21050549

Guan, X., Mao, J., Yan, B., Wang, D., Zhang, X., & Zhou, H. (2018). A comparative study among ferulic acid, caffeic acid content and axtioxidation in different extracts of Angelica sinesis. *Natural Product Research and Development, 12*(30), 2033-2038. https://doi.org/10.16333/j.1001-6880.2018.12.001

Guan, Z., Wu, S., Guo, B., Wei, H., Wang, Y., & Yang, X. (2015). Comparative study on active

ingredient content in different parts of Sophora flavescens. *Modern Chinese Medicine, 17*(12), 1272-1274. https://doi.org/10.13313/j.issn.1673-4890.2015.12.010

Guo, J. (2007). *Compariative research on the pharmaceutic and pharmacodynamics among different breed Bulbus Fritillaria Thunbergii* [Master's thesis, Heilongjiang University of Chinese Medicine]. China National Knowledge Infrastructure.

Hao, W., Li, D., & Yang, C. (2017). Determination of matrine and oxymatrine in Sophora Flavescens by reverse phase liquid chromatography. *Chemical Analysis and Meterage, 26*(4), 68-70. https://doi.org/10.3969/j.issn.1008-6145.2017.04.017

He, X., Fang, J., Huang, L., Wang, J., & Huang, X. (2015). Sophora flavescens Ait.: Traditional usage, phytochemistry and pharmacology of an important traditional Chinese medicine. *Journal of Ethnopharmacology, 172*(2015), 10-29. https://doi.org/10.1016/j.jep.2015.06.010

Huang, C. (2016). Determination of the contents of peimine A and peimine B in Fritillaria thunbergii by ELSD *China Health Standard Management, 7*(18), 139-141.

Huang, Q., Xu, L., Wen-Shan, Q., Zhao-Hong, Y., Wen-Yu, H., Long-Yuan, L., Jin-Feng, L., Shao, L., & Hong-Yan, M. (2017). TLC bioautography-guided isolation of antioxidant activity components of extracts from Sophora flavescens Ait. *European Food Research and Technology, 243*(7), 1127-1136. https://doi.org/10.1007/s00217-016-2820-z

Huang, R. (2017). Determination of active compounds from different medical parts of Angelica sinensis. *Nei Mongol Journal of Traditional Chinese Medicine, 18*(36), 117-118. https://doi.org/10.3969/j.issn.1006-0979.2017.18.117

Huang, R., Liu, Y., Zhao, L. L., Chen, X. X., Wang, F., Cai, W., & Chen, L. (2017). A new flavonoid from Sophora flavescens Ait. *Natural Product Research, 31*(19), 2228-2232. https://doi.org/10.1080/14786419.2017.1297992

Ji, M., Zeng, K., & Wang, H. (2017). Simultaneous determination of seven alkaloid constituents in Sophora Flavescens. *China Pharmacist, 20*(1), 60-63. https://doi.org/10.3969/j.issn.1008-049X.2017.01.016

Jiang, H., Zhao, X., & Lin, W. (2019). Qualitative analysis of multiple cumarins in Angelicae Sinensis Radix based on HPLC-Q-TOF-MS/MS. *Chinese Journal of Experimental Traditional Medical Formulae, 25*(13), 157-162. https://doi.org/10.13422/j.cnki.syfjx.20190918

Jin, X., Xu, D., Xu, Y., Cui, D., Xiao, X., Tian, Z., Lu, Y., & Zhen, Q. (1993). The structure identification of zhepiresionol. *Acta Pharmacologica Sinica, 28*(3), 212-215.

Kim, E. J., Yoon, Y. P., Woo, K. W., Kim, J. H., Min, S. Y., Lee, H. J., Lee, S. K., Hong, J. H., Lee, K. R., & Lee, C. J. (2016). Verticine, ebeiedine and suchengbeisine isolated from the bulbs of Fritillaria thunbergii Miq. inhibited the gene expression and production of MUC5AC mucin from human airway epithelial cells. *Phytomedicine, 23*(2), 95-104. https://doi.org/10.1016/j.phymed.2015.12.016

Kim, J. H., Cho, C. W., Kim, H. Y., Kim, K. T., Choi, G. S., Kim, H. H., Cho, I. S., Kwon, S. J., Choi, S. K., Yoon, J. Y., Yang, S. Y., Kang, J. S., & Kim, Y. H. (2017). α-Glucosidase inhibition by prenylated and lavandulyl compounds from Sophora flavescens roots and in silico analysis [Article]. *International Journal of Biological Macromolecules, 102*, 960-969. https://doi.org/10.1016/j.ijbiomac.2017.04.092

Kim, J. H., Cho, I. S., So, Y. K., Kim, H. H., & Kim, Y. H. (2018). Kushenol A and 8-prenylkaempferol, tyrosinase inhibitors, derived from Sophora flavescens [Article]. *Journal of Enzyme Inhibition and Medicinal Chemistry, 33*(1), 1048-1054. https://doi.org/10.1080/14756366.2018.1477776

Kitajima, J., Komori, T., & Kawasaki, T. (1982). Studies on the constituents of the crude drug "Fritillariae bulbus." III. On the diterpenoid constituents of fresh bulbs of Fritillaria thunbergii Miq. *Chemical and Pharmaceutical Bulletin, 30*(11), 3912-3921. https://doi.org/10.1248/cpb.30.3912

Kitajima, J., Noda, N., Ida, Y., Komori, T., & Kawasaki, T. (1982). Studies on the constituents of the crude drug "Fritillariae bulbus." IV. On the diterpenoid constituents of the crudr "Fritillariae bulbus". *Chemical and Pharmaceutical Bulletin, 30*(11), 3922-3931. https://doi.org/10.1248/cpb.30.3922

Lee, J., Jung, J., Son, S. H., Kim, H. B., Noh, Y. H., Min, S. R., Park, K. H., Kim, D. S., Park, S. U., Lee, H. S., Kim, C. Y., Kim, H. S., Lee, H. K., & Kim, H. (2018). Profiling of the major phenolic compounds and their biosynthesis genes in Sophora flavescens Aiton. *Scientific World Journal*, *2018*, e6218430, Article 6218430. https://doi.org/10.1155/2018/6218430

Li, J., Wei, Y., Qin, X., & Li, Z. (2017). Chemical comparison on different parts of Angelica sinensis Radix based on NMR metabolomics. *Chinese Traditional and Herbal Drugs, 48*(7), 1409-1415. https://doi.org/10.7501/j.issn.0253-2670.2017.07.025

Li, X., Dong, X., Bao, B., Hang, L., Liu, B., & Zhang, P. (2019). Rapid analysis and identification of chemical components of Sophora Flavescens based on Q-exactive high resolution mass spectrometry. *Journal of Chinese Medicinal Materials, 42*(1), 103-109. https://doi.org/10.13863/j.issn1001-4454.2019.01.023

Li, Z., Gu, H., Kang, L., Zhang, Y., & Song, h. (2016). Identification of key aroma-active compounds in Radix Angelica sinensis and study on its variation during boiling process. *Science and Technology of Food Industry, 37*(9), 311-316. https://doi.org/10.13386/j.issn.1002-0306.2016.09.052

Liu, J., Wang, X., Xiang, Z., & Li, Y. (2008). Study on the principal component analysis method to determination of trace elements of Fritillaria thunbergii Miq. *Guangdong Trace Elements Science, 15*(12), 31-35.

Liu, L., Yang, J., & Hong, J. (2018). Determination and evaluation of heavy metals in Radix Sophora flavescens Ait. in different origins. *Guangdong Chemical Industry, 20*(45), 34-36. https://doi.org/10.3969/j.issn.1007-1865.2018.20.016

Liu, L., Yue, M., Li, W., Peng, C., & Xiong, L. (2016). Differences from chemical components andprotective effect on acute cerebral injury of mice between the oils from Angelica sinensis and Ligusticum Chuanxiong. *Pharmacology and Clinics of Chinese Materia Medica, 32*(6), 105-108. https://doi.org/10.13412/j.cnki.zyyl.2016.06.029

Liu, Q. (2018). Study on the structure identification of lactones in Angelica sinensis. *For all Health, 6*(12), 118-119. https://doi.org/10.3969/j.issn.1009-6019(z).2018.06.135

Lou, Y., Cai, H., Liu, X., Tu, S., Pei, K., Zhao, Y., Cao, G., Li, S., Qin, K., & Cai, B. (2014). Element analysis and characteristic identification of non-fumigated and sulfur-fumigated Fritillaria thunbergii Miq. using microwave digestion-inductively coupled plasma atomic emission spectrometry combined with Fourier transform infrared spectrometry. *Pharmacognosy Magazine, 10*(Suppl 1), S30-36. https://doi.org/10.4103/0973-1296.127337

Luo, L., Ma, L., Liu, S., & Zhu, M. (2017). Determination of the 1-(3',4'-Dihydroxycinnamoyl)-cyclopentane-2,3-diol in Angelicae Sinensis Radix by HPLC. *Chinese Journal of Modern Applied Pharmacy, 34*(9), 1296-1299. https://doi.org/10.13748/j.cnki.issn1007-7693.2017.09.017

Ma, J., Wei, W., Guo, T., Li, S., Wang, Y., & Jin, L. (2016). Study on chemical constituents of n-butanol extracts from Angelica sinensis. *China Food, 2016*(3), 62-64. https://doi.org/10.3969/j.issn.1006-6195.2016.03.032

Ma, J. Y., Zhao, D. R., Yang, T., Liu, D., Li, R. T., & Li, H. M. (2019). Prenylflavanones isolated from Sophora flavescens. *Phytochemistry Letters, 29*, 138-141. https://doi.org/10.1016/j.phytol.2018.11.025

Ma, W., Rong, J., Sheng, Z., & Hu, J. (2008). Study on HPLC specific chromatogram of Fritillaric thunbergii. *Chinese Journal of Hospital Pharmacy, 28*(17), 1458-1460.

Ma, Y., Wen, Q., Tong, X., Fan, B., & Sun, J. (2014). Study on serum pharmacochemistry of Fritillaria thunbergii Miq. by LC-MS in rats. *Chinese Journal of Pharmacovigilance, 11*(12), 714-720.

Ma, Y., Xu, S., Liao, H., He, F., Zhong, F., Wen, W., & Wu, D. (2017). HPLC simultaneous determination of seven components in SFE-CO2 extract from Chuanxiong Rhizoma and Angelicae Sinensis Radix and stability investigation of coniferyl ferulate. *Chinese Journal of Experimental Traditional Medical Formulae, 23*(9), 24-29. https://doi.org/10.13422 /j.cnki.syfjx.2017090024

Morimoto, H., & Kimata, S. (1960). Studies on the components of Fritillaria Thunbergii Miq. I. isolation of peimine and its new glycoside. *Chemical and Pharmaceutical Bulletin, 8*(4), 302-

307. https://doi.org/10.1248/cpb.8.302
National Comprehensive Cancer Network. (2019). *NCCN clinical practice guidelines in oncology-prostate cancer (version 4).* Retrieved 1 Jan 2020 from https://www.nccn.org/professionals/physician_gls/pdf/prostate.pdf
Pan, Z., Wan, Z., Zeng, H., Yang, S., & Cai, W. (2017). Using solid ionic liquid at room temperature as extraction agent for the separation and determination of ferulic acid in Angelica sinensis. In Chinese Chemical Society (Eds.). *The 21st National Chromatography Academic Conference and Instrument Exhibition* (pp. 412-413). Chinese Chemical Society.
Pei, J., He, Y., Yao, H., Wang, Y., Feng, X., Ge, C., & Huang, L. (2017). Analysis of the volatile constituents in different parts of Angelica sinensis by GC-MS and orthogonal projection resolution. *Chinese Journal of Pharmaceutical Analysis, 37*(5), 826-831. https://doi.org/10.16155/j.0254-1793.2017.05.12
Qin, S., Zhao, X., Geng, L., Zhou, H., Liu, M., Li, F., & Zhao, C. (2017). Determination of 6 elements in Angelica sinensis by microwave digestion-AAS. *Guangzhou Chemical Industry, 44*(1), 97-98. https://doi.org/0.3969/j.issn.1001-9677.2017.01.033
Qin, Z. (2017). Determination of copper in Angelica sinensis. *Seed Scicence & Technology, 3*(35), 106, 109. https://doi.org/10.3969/j.issn.1005-2690.2017.03.075
Rong, Y., & Zhang, Y. (2017). Determination of four elements (copper, cadmium, mercury, arsenic) in Angelicae Sinensis Radix by microwave digestion with ICP-MS. *Strait Pharmaceutical Journal, 29*(1), 63-64. https://doi.org/10.3969/j.issn.1674-7860.2017.13.021
Su, B. (2019). Study on effective chemical constituents in different parts of Angelica sinensis. *China Journal of Pharmaceutical Economics, 1*(14), 34-37. https://doi.org/10.12010/j.issn.1673-5846.2019.01.007
Suh, W. S., Lee, S. Y., Park, J. E., Kim, D. H., Kim, S., & Lee, K. R. (2018). Two new steroidal alkaloids from the bulbs of fritillaria thunbergii. *Heterocycles, 96*(5), 921-930. https://doi.org/10.3987/COM-18-13891
Sun, L., Guo, J., Yan, Y., Yu, H., Li, W., Zhang, L., & Song, X. (2017). Study on the chemical constituents and antibacterial activity of Radix Sophora Flavescens. *Journal of Liaoning University of Traditional Chinese Medicine, 19*(11), 49-53. https://doi.org/10.13194/j.issn.1673-842x.2017.11.014
Sun, X., Li, B., Huang, W., Wang, N., Song, Z., & Tang, Z. (2019). Determination of ferulic acid from the alcohol extract of Angelica sinensis and the investigation of its antioxidant activity. *Shanxi journal of Agricultural Sciences, 65*(1), 30-32.
Takahashi, T., Ishino, A., Arai, T., Hamada, C., Nakazawa, Y., Iwabuchi, T., & Tajima, M. (2016). Improvement of androgenetic alopecia with topical Sophora flavescens Aiton extract, and identification of the two active compounds in the extract that stimulate proliferation of human hair keratinocytes. *Clinical and Experimental Dermatology, 41*(3), 302-307. https://doi.org/10.1111/ced.12753
Wang, B., Li, X., & Zhang, X. (2018). Determination of Matrine by GC and HPLC. *Agrochemicals, 57*(7), 497-499. https://doi.org/10.16820/j.cnki.1006-0413.2018.07.008
Wang, C., Liu, H., Wang, X., Tong, X., & Hu, S. (2017). Determination of alkaloids in Sophora Flavescens by high performance capillary electrophoresis. *Chemical Engineering Design Communications, 43*(2), 115, 118. https://doi.org/10.3969/j.issn.1003-6490.2017.02.092
Wang, H., Dong, Q., Wu, F., Tan, J., Li, P., Liu, J., & Lin, H. (2019). Simultaneous determination of 12 essential trace elements and 5 heavy metal elements in Angelica Sinensis (Oliv.) Diels by ICP-MS. *Special Wild Economic Animal and Plant Research, 2*(41), 69-73. https://doi.org/10.16720/j.cnki.tcyj.2019.02.017
Wang, J., Ge, B., Li, Z., Guan, F., & Li, F. (2016). Structural analysis and immunoregulation activity comparison of five polysaccharides from Angelica sinensis. *Carbohydrate Polymers, 140*, 6-12. https://doi.org/10.1016/j.carbpol.2015.12.050
Wang, L., Fan, S., Xu, F., Liu, G., & Shang, M. (2019). Analysis of polarity components of Angelicae Sinensis Radix and its metabolites in rats by HPLC-MSn. *Chinese Journal of Chinese Materia Medica, 2019*(22), 1-14. https://doi.org/10.19540/j.cnki.cjcmm.20190522.503
Wang, L., Huang, S., Chen, B., Zang, X.-Y., Su, D., Liang, J., Xu, F., Liu, G.-X., Shang, M.-Y., &

Cai, S.-Q. (2016). Characterization of the anticoagulative constituents of Angelicae Sinensis Radix and their metabolites in rats by HPLC-DAD-ESI-IT-TOF-MSn. *Planta Medica, 82*(4), 362-370. https://doi.org/10.1055/s-0035-1558309

Wang, N., Zhang, Q., Xin, H., Shou, D., & Qin, L. (2017). Osteoblast cell membrane chromatography coupled with liquid chromatography and time-of-flight mass spectrometry for screening specific active components from traditional Chinese medicines. *Journal of Separation Science, 40*(22), 4311-4319. https://doi.org/10.1002/jssc.201700688

Wang, Q., Xu, J., Li, X., Zhang, D., Han, Y., & Zhang, X. (2017). Comprehensive two-dimensional PC-3 prostate cancer cell membrane chromatography for screening anti-tumor components from Radix Sophorae flavescentis. *Journal of Separation Science*, *40*(13), 2688-2693. https://doi.org/10.1002/jssc.201700208

Wang, X., Zhao, Z., Zhang, Y., Shi, X., Cheng, F., Guo, M., & Wen, X. (2018). Comparative analysis of volatile compounds in Angelica Sinensis by different acquisition methods with GC-MS technique. *Journal of Traditional Chinese Veterinary Medicine, 1*(37), 61-65. https://doi.org/10.13823/j.cnki.jtcvm.2018.01.019

Wang, Y., Qin, L., Li, P., & Li, Y. (2016). Effect of ethanol at different concentrations on liver protection active components in Angelica Sinensis extracted by ultrasonic wave. *China Pharmacist*, *19*(8), 1479-1482. https://doi.org/10.3969/j.issn.1008-049X.2016.08.015

Wei, W. (2016). *Studies on chemical constituents from n-buOH extraction of Angelica sinensis* [Master's thesis, Lanzhou University of Technology]. China National Knowledge Infrastructure.

Wei, W., Zeng, R., Gu, C., Qu, Y., & Huang, L. (2016). Angelica sinensis in China-A review of botanical profile, ethnopharmacology, phytochemistry and chemical analysis. *Journal of Ethnopharmacology, 190*(2016), 116-141. https://doi.org/10.1016/j.jep.2016.05.023

Weng, Z., Zeng, F., Zhu, Z., Qian, D., Guo, S., Wang, H., & Duan, J. A. (2018). Comparative analysis of sixteen flavonoids from different parts of Sophora flavescens Ait. by ultra high-performance liquid chromatography–tandem mass spectrometry. *Journal of Pharmaceutical and Biomedical Analysis*, *156*, 214-220. https://doi.org/10.1016/j.jpba.2018.04.046

Wu, C., Wu, M., & Chen, Y. (2018). Selective extraction of n-butylidenephthalide from Angelica sinensis (Danggui) by using functionalized iron oxide magnetic nanoparticles as trapping probes. *Analytical Methods*, *10*(14), 1593-1601. https://doi.org/10.1039/c8ay00065d

Wu, D., Wang, H., & Shen, P. (1998). Determination of selenium with different valences in Zhebeimu by spectrophotometry. *Guangdong Trace Elements Science*, *5*(7), 66-68.

Wu, G., Yang, X., Deng, Y., Yang, Z., & Yang, Y. (2018). Fingerprints and component analysis of aqueous decoction from different medical parts of Angelica sinensis. *Chinese Traditional Patent Medicine*, *40*(4), 890-894. https://doi.org/10.3969/j.issn.1001-1528.2018.04.025

Wu, X., Chan, S. W., Ma, J., Li, P., Shaw, P. C., & Lin, G. (2018). Investigation of association of chemical profiles with the tracheobronchial relaxant activity of Chinese medicinal herb Beimu derived from various Fritillaria species. *Journal of Ethnopharmacology, 210*, 39-46. https://doi.org/10.1016/j.jep.2017.08.027

Wu, X., Zhang, S., Cai, Y., & Ma, K. (2017). Study on using capillary electrophoresis to separate three alkaloids from Sophora flavescens. *Journal of Changzhi University, 34*(5), 497-499. https://doi.org/10.3969/j.issn.1673-2014.2017.05.004

Wu, Y. (2016). Study on the separation and pharmacodynamical research of effective components for Sophora Flavescens Ait in Tranquilizing mind [Master's thesis, Zhejiang Chinese Medical University]. China National Knowledge Infrastructure.

Wu, Y., Ma, M., & Nian, H. (2016). Isolation of chemical composition of Angelica and identification of lactone component structure. *World Journal of Integrated Traditional and Western Medicine*, *11*(5), 649-652. https://doi.org/10.13915/j.cnki.sjzx.160514

Wu, Z., & Zheng, S. (1997). Grey relational analysis and determination of elements in Fritillaria Thunbergii Bulb and Fritillariae Cirrhosae Bulb. *Journal of Chinese Medicinal Materials, 20*(6), 291-293.

Xu, C. (2016). Isolation and determination of the chemical compounds from Angelica sinensis. *Asia-Pacific Traditional Medicine*, *12*(24), 42-43. https://doi.org/10.11954/ytctyy.201624015

Xue, Y., & Gu, H. (2005). Determination of peimine and peiminine in Fritillaria thunbergii by HPLC-ELSD. *Acta Pharmacologica Sinica, 40*(6), 550-552.

Yang, Y., Tang, L., Yao, Q., Shang, Q., Liu, D., & Xu, X. (2016). Determination of matrine and oxymatrine in Radix Sophorae Flavescentis by TLC scanning. *Journal of Traditional Chinese Veterinary Medicine, 1*(35), 26-28. https://doi.org/10.13823/j.cnki.jtcvm.2016.01.007

Yang, Z., Li, X., Liu, M., Zhang, W., Shan, B., Song, C., & Deng, W. z. (2016). Isolation of antibacterial activity constituents of total flavonoids from Sophora Flavescens and simultaneous determination of seven flavonoids by HPLC. *Chinese Pharmaceutical Journal, 24*(51), 2169-2174. https://doi.org/10.11669/cpj.2016.24.024

Yu, R., Cheng, Z., & Gong, X. (2007). Determination and comparison of trace elements in four sort of Bulbus Fritillariae by microwave digestion-atomic absorption spectrometry. *Spectroscopy and Spectral Analysis, 27*(12), 2591-2594.

Yuan, X., Shi, J., Yang, Y., Luan, J., Gao, J., & Wang, Y. (2010). Establishment of element fingerprint and multielement analysis of Fritillaria thunbergii by inductively coupled plasma optical emission spectrometry. *Biological Trace Element Research, 135*(1-3), 304-313. https://doi.org/10.1007/s12011-010-8627-z

Zhang, C., Sun, L., Chen, R., Su, J. H., Zhang, H. F., Gu, B. R., Xing, Y. W., & Xue, M. (2016). Multiple analytical methods for identification and quality evaluation of Fritillariae Thunbergii Bulbus based on biological single molecules by high-performance liquid chromatography. *Journal of Separation Science, 39*(18), 3536-3543. https://doi.org/10.1002/jssc.201600356

Zhang, J., Lao, A., Huang, H., Ma, G., & Xu, R. (1992). Studies on chemical constituents of Fritillaria thunbergii Miq. III. isolation and identification of Zhebeinone. *Acta Pharmacologica Sinica, 27*(6), 472-475.

Zhang, J., Lao, A., Ma, G., & Xu, R. (1991). Studies on chemical constituents of Fritillaria thunbergii Miq. II. *Acta Pharmacologica Sinica, 33*(12), 923-926.

Zhang, J., Lao, A., & Xu, R. (1993a). Studies on chemical constituents of Fritillaria thunbergii Miq. IV. *Chinese Bulletin of Botany, 35*(3), 238-241.

Zhang, J., Lao, A., & Xu, R. (1993b). Studies on the chemical constituents of fresh bulbs of Fritillaria thunbergii Miq. *China Journal of Chinese Materia Medica, 18*(6), 354-355.

Zhang, Q., Yu, J., Wang, Y., & Su, W. (2016). Selective Extraction of Flavonoids from Sophora flavescens Ait. by Mechanochemistry. *Molecules, 21*(8), 989-989. https://doi.org/10.3390/molecules21080989

Zhang, Q., Yu, J., Zhang, L., Hu, M., Xu, Y., & Su, W. (2016). Extraction, characterization, and biological activity of polysaccharides from Sophora flavescens Ait. *International Journal of Biological Macromolecules, 93*, 459-467. https://doi.org/10.1016/j.ijbiomac.2016.08.052

Zhang, R., Hu, S., Chen, X., & Bai, X. (2016). Dispersive liquid-liquid microextraction combined with high-performance liquid chromatography for the simultaneous analysis of matrine alkaloids in traditional Chinese medicine. *Journal of Chromatographic Science, 54*(9), 1687-1693. https://doi.org/10.1093/chromsci/bmw114

Zhang, W., Zhu, D., Fan, H., Liu, X., Wan, Q., Wu, X., Liu, P., & Tang, J. Z. (2015). Simultaneous extraction and purification of alkaloids from Sophora flavescens Ait. by microwave-assisted aqueous two-phase extraction with ethanol/ammonia sulfate system. *Separation and Purification Technology, 141*, 113-123. https://doi.org/10.1016/j.seppur.2014.11.014

Zhang, Y. B., Zhan, L. Q., Li, G. Q., Wang, F., Wang, Y., Li, Y. L., Ye, W. C., & Wang, G. C. (2016). Dimeric matrine-type alkaloids from the roots of Sophora flavescens and their anti-hepatitis B virus activities. *Journal of Organic Chemistry, 81*(15), 6273-6280. https://doi.org/10.1021/acs.joc.6b00804

Zhao, D., Xue, Y., Chen, X., Liu, D., Li, R., & Li, H. (2018). A study on the quinolizidine alkaloids of Sophora flavescens. *Journal of Kunming University of Science and Technology (Natural Science), 43*(4), 93-97. https://doi.org/10.16112/j.cnki.53-1223/n.2018.04.013

Zhao, F. (2015). *Simultaneous analysis of chemical components and contents in various organs of Sophora Flavescens Ait.* [Master's thesis, Institute of Chinese Materia Medica China Academy of Chinese Medical Sciences]. China National Knowledge Infrastructure.

Zhao, M., Yang, S., Sun, Y., Li, F., Zhang, S., & Jiao, J. (2018). Gas chromatography-mass

spectrometry analysis of volatile oil of Angelica sinensis root dry chemical composition. *Chemical World*, *59*(4), 231-234. https://doi.org/10.19500/j.cnki.0367-6358.20170615

Zhou, J. L., Xin, G. Z., Shi, Z. Q., Ren, M. T., Qi, L. W., Li, H. J., & Li, P. (2010). Characterization and identification of steroidal alkaloids in Fritillaria species using liquid chromatography coupled with electrospray ionization quadrupole time-of-flight tandem mass spectrometry. *Journal of Chromatography A*, *1217*(45), 7109-7122. https://doi.org/10.1016/j.chroma.2010.09.019

Zhou, M., Ma, X., Ding, G., Wang, Z., Liu, D., Tong, Y., Zhou, H., Gao, J., Hou, Y., Jiang, M., & Bai, G. (2017). Comparison and evaluation of antimuscarinic and anti-inflammatory effects of five Bulbus fritillariae species based on UPLC-Q/TOF integrated dual-luciferase reporter assay, PCA and ANN analysis. *Journal of Chromatography B*, *1041-1042*, 60-69. https://doi.org/10.1016/j.jchromb.2016.12.012

Zhou, Y., Gao, X., Wu, C., & Wu, Y. (2014). Bioaccessibility and safety assessment of trace elements from decoction of "Zhebawei" herbal medicines by in vitro digestion method. *Journal of Trace Elements in Medicine and Biology*, *28*(2), 173-178. https://doi.org/10.1016/j.jtemb.2013.12.007

Zhu, H., Yang, Y.-N., Xu, K., Xie, J., Feng, Z.-M., Jiang, J.-S., & Zhang, P.-C. (2017). Sophopterocarpan A, a novel pterocarpine derivative with a benzotetrahydrofuran-fused bicyclo [3.3.1] nonane from Sophora flavescens. *Organic & Biomolecular Chemistry*, *15*(26), 5480-5483. https://doi.org/10.1039/c7ob01261f

Zhu, S. (2018). Screening of effective parts and separation analysis of chemical composition based on blood nourishing, blood circulation and laxative of Angelica Sinensis [Master's thesis, Gansu University of Chinese Medicine]. China National Knowledge Infrastructure.

Zhu, S., Guo, S., Duan, J. A., Qian, D., Yan, H., Sha, X., & Zhu, Z. (2017). UHPLC-TQ-MS coupled with multivariate statistical analysis to characterize nucleosides, nucleobases and amino acids in Angelicae Sinensis Radix obtained by different drying methods. *Molecules*, *22*(6), 918-918. https://doi.org/10.3390/molecules22060918
